# Conceptual Progress for Explaining and Predicting Self-Organization on Anodized Aluminum Surfaces

**DOI:** 10.3390/nano11092271

**Published:** 2021-08-31

**Authors:** Mikhail Pashchanka

**Affiliations:** Department of Chemistry, Eduard-Zintl-Institute, Technical University of Darmstadt, Alarich-Weiss-Straße 12, 64287 Darmstadt, Germany; mikhail.pashchanka@gmail.com

**Keywords:** porous anodic alumina (PAA), chaos and self-organization theory, electroconvection, colloidal gel model, anion exchange, DLVO theory, fluid mechanics, surface chemistry, surface energy reduction, electrochemistry

## Abstract

Over the past few years, researchers have made numerous breakthroughs in the field of aluminum anodizing and faced the problem of the lack of adequate theoretical models for the interpretation of some new experimental findings. For instance, spontaneously formed anodic alumina nanofibers and petal-like patterns, flower-like structures observed under AC anodizing conditions, and hierarchical pores whose diameters range from several nanometers to sub-millimeters could be explained neither by the classical field-assisted dissolution theory nor by the plastic flow model. In addition, difficulties arose in explaining the basic indicators of porous film growth, such as the nonlinear current–voltage characteristics of electrochemical cells or the evolution of hexagonal pore patterns at the early stages of anodizing experiments. Such a conceptual crisis resulted in new multidisciplinary investigations and the development of novel theoretical models, whose evolution is discussed at length in this review work. The particular focus of this paper is on the recently developed electroconvection-based theories that allowed making truly remarkable advances in understanding the porous anodic alumina formation process in the last 15 years. Some explanation of the synergy between electrode reactions and transport processes leading to self-organization is provided. Finally, future prospects for the synthesis of novel anodic architectures are discussed.

## 1. Introduction

**Figure 1:**
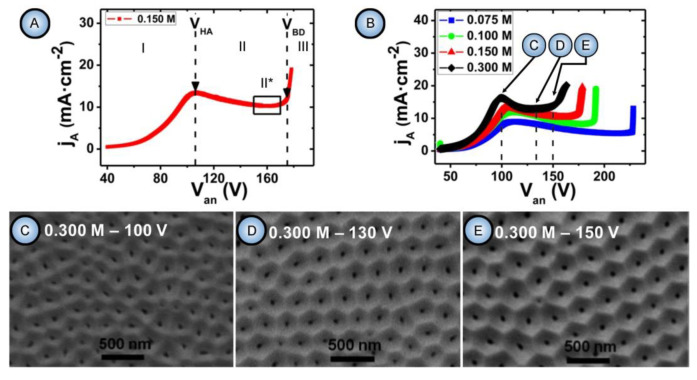
(**A**) A typical nonlinear *j*−∆*U* curve for the formation of PAA divided into four stages labeled as I (“mild anodizing” regime, MA), II (“hard anodizing” regime, HA), II* (current plateau region which corresponds to the HA self-ordering voltages and is preceded by an intermediate “chaotic” state), III (dielectric breakdown), V_HA_ (the beginning of II), and V_BD_ (the beginning of III); (**B**) *j*–∆*U* curves obtained for 0.075–0.3 M oxalic acid electrolyte solutions containing 5–10 vol % of ethanol; (**C**–**E**) SEM images demonstrating different degrees of pore ordering obtained at 100–150 V anodizing voltages using a 0.3 M oxalic acid electrolyte solution (corresponds to the black *j*–∆*U* curve in (**B**), on which the tested voltages are shown by arrows and dashed lines). Adapted with permission from [5]. Copyright 2015 American Chemical Society.

It was the novelist Grace McCleen who once said: “Miracles do not have to be big, and they can happen in the unlikeliest places.” This quote obviously did not have anything to do with exact sciences or, specifically, with the stochastic nature of self-organization in non-living matter. However, it certainly has a great metaphorical value that may help to explain some basic principles of such complex phenomena in a simpler manner. Indeed, for the miracle called “spontaneous self-organization” to occur, a system must be stochastic. That is to say that such a system must always include different random, probabilistic processes because instabilities and accidental fluctuations are the necessary factors leading to evolutionary patterns in the real-world systems. One vivid example of such “chaotic” evolution is the well-known sequence of “mild anodizing” (MA) [1,2] and “hard anodizing” (HA) [3,4] self-ordering regimes observed during honeycomb-like porous anodic alumina (PAA) formation under gradually increased anodizing voltages (Figure 1A). The recent study by Vega et al. showed that the MA and the HA voltage ranges in which self-ordering occurs are typically separated by a rather broad interval (that is recognizable on a recorded linear sweep voltammogram) in which no formation of well-ordered pore arrays can be observed (see Figure 1A–E) [5]. This experimental investigation confirms that the new stable porous structure with a larger self-ordering periodicity which is formed under the HA regime does not evolve from the previous array of smaller MA-generated close-packed hexagonal cells after their continuous uniform expansion upon the gradual linear transition from “mild” to “hard” anodizing voltages. Instead, the new well-ordered textures emerge from an intermediate chaotic state. Thus, the entire evolution process where chaos plays its constructive role includes not only gradual quantitative changes but also qualitative transitions and sharp quantitative changes in between due to the complex interplay of different involved factors. This observation leads us to the next important property of self-organizing systems—namely, their nonlinearity. Indeed, as demonstrated by voltammetry measurements in the above-mentioned work by Vega et al. (Figure 1A,B), there is a nonlinear relationship between electrical current density *j* and applied potential difference ∆*U* in a typical anodizing cell designed for PAA growth (in other words, Ohm’s law is not met) [5]. The MA self-ordering interval (where current rises exponentially; see stage I in Figure 1A) is separated from the HA self-ordering interval by a local maximum and a short plateau region (within which, as discussed previously, disordered PAA is formed; see Figure 1C) due to some current-limiting mechanisms.

**Figure 2:**
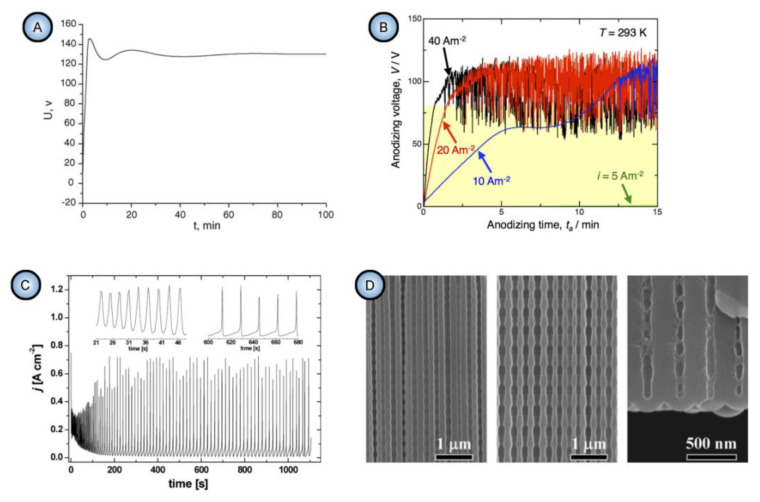
Oscillatory kinetic behavior observed during galvanostatic (**A**,**B**) and potentiostatic (**C**,**D**) anodic oxidation of aluminum. (**A**) A voltage–time curve obtained at 1.0 A dm^−2^ and 12 ± 1 °C using a mixed electrolyte of phosphoric acid and organic acid and cerium salt (Wang and Wang, [10]); (**B**) Unstable oscillations observed during galvanostatic anodizing of Al at 5–40 A m^−2^ using a pyrophosphoric acid solution at 20 °C (Kikuchi et al. [11]); (**C**) Spontaneous current oscillations during potentiostatic anodizing of aluminum at 160 V using a 0.3 M oxalic acid solution at 5 °C (Lee et al. [12]); (**D**) Cross-sectional SEM micrographs demonstrating the nanochannel diameter modulation resulting from the temporal oscillations of the electric current density shown in C [12]. Reproduced with permissions. Copyrights 2006 Elsevier, 2010 Wiley, 2014 Nature.

In general, the nonlinearity in such dynamic self-organizing systems (that is, in which spontaneous self-organization of matter into patterns occurs under a certain set of conditions) means that the response of such a system has to be out of proportion to the external influence (in our case, to the externally controlled gradual increase in the applied voltage ∆*U*). Such rapid exponential amplification of the electrical current followed by its inhibition within the transitional interval can also indirectly suggest the possible presence of another necessary kinetic condition for spontaneous self-organization—namely, so-called positive and negative feedback loops that alternately dominate the dynamic process. Actually, if a dynamic system exhibits temporal oscillatory behavior, this typically indicates that there exist properly operating (fast positive and slow negative) feedback loops [6]. Such oscillations usually appear upon the increase in the corresponding operating parameters (e.g., temperature *T* or applied voltage ∆*U*) to the values required to bring a system out of its trivial stable steady-state toward a so-called “unstable steady-state”, where self-organization occurs [7]. In this connection, it must be noted that self-organized honeycomb-like patterns on anodized aluminum commonly arise at voltages one or two orders of magnitude higher than typically required for the classical electrochemical method (e.g., 25–160 V for achieving 50–420 nm interpore distances [8], or even 260–450 V for increasing the cell diameter in PAA up to 1.1 µm [9]). Moreover, there is also multiple experimental evidence of the oscillatory behavior of different electrochemical systems where PAA is formed. For instance, damped oscillations of the ∆*U*–*t* (voltage against time) curve have been reported for the galvanostatic anodizing experiments conducted in a solution containing a mixed electrolyte of phosphoric acid and organic acid and cerium salt (Figure 2A) [10]. Unstable oscillations have also been observed during experiments performed using a concentrated pyrophosphoric acid solution (Figure 2B) [11]. Another striking example of oscillatory kinetic behavior during Al anodizing has been reported by Lee et al., who observed spontaneous electrical current oscillations during potentiostatic HA performed at 140–200 V using unstirred 0.3 M oxalic acid electrolyte (Figure 2C) [12]. The observed consequence of such oscillatory behavior was the growth of modulated pore structures where periodic changes of nanochannel diameters corresponded to the amplitude and the period of electrical current oscillations (Figure 2D). Here, it is worth remarking that PAA formation takes place directly at the metal/alumina interface (that is, at the side of a growing PAA lamina which is frequently earmarked in the literature as the “bottom side”) where the geometrical parameters of cells and pores are defined by the current electrolysis conditions. This two-dimensional cellular pattern is continuously translated into the direction normal to the metal substrate surface, forming a three-dimensional system of aligned high-aspect-ratio tubular channels. Thus, every accidental perturbation or intentional modification of anodizing conditions during the growth of PAA will be “recorded” in situ through a corresponding instant readjustment of geometrical parameters of these responsive channels. In other words, a clean and detailed cross-sectional view of a PAA specimen underneath a microscope allows obtaining information about the growth of channels ex situ by “reading” their morphological features in the reverse direction, from top to bottom, and to some extent reconstructing a history of an anodizing process. The pore size modulation synchronized with spontaneous current oscillations demonstrated by Lee et al. is a particularly good example of such a behavior–morphology relationship [12].

**Figure 3:**
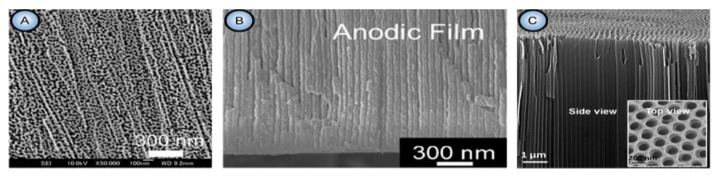
SEM micrographs of the top surfaces and the cross-sections of porous layers obtained by anodic oxidation of different “valve metals”, such as iron (**A**,**B**) and titanium (**C**). Fe was anodized in ethylene glycol solution containing 0.1 mol L^−1^ NH_4_F and 0.5 mol L^−1^ H_2_O at 50 A m^−2^ and 20 °C (Konno et al. [18]); Ti was anodized in ethylene glycol containing 0.3 wt % NH_4_F and 2 vol % H_2_O at 50 V (Xue et al. [19]). Reproduced with permissions. Copyrights 2012 Springer, American Chemical Society.

To sum up, the morphological features of PAA in all three dimensions are evidently related to nonlinear dependencies between various operating parameters of anodizing (∆*U*, *j*, *T*, etc.) and feedback loops. The spontaneous formation of such type of honeycomb-like anodic textures is commonly considered in different theoretical models to arise from a complex mix of opposing factors and a synergistic effect of their interaction (for instance, simultaneous competing processes of oxide growth and dissolution [13,14,15] or emergence of oppositely directed ionic flows due to different driving forces [16,17]). It should be noted that PAA is not the only example of self-organized patterns on anodized metals. Self-organization on the basis of analogous principles and mechanisms has been demonstrated for the entire range of so-called “valve metals” (e.g., iron [18] or titanium [19]; see Figure 3) on which porous layers with different degrees of orderliness arise upon anodizing under appropriately selected conditions. This inspires researchers to experiment further with different metals and alloys (especially Al-based) in attempts to extend the range of suitable working electrode materials [20].

Self-organized PAA architectures have recently received growing interest in many innovative research areas, such as nano-optics and nanophotonics [21,22,23,24], surface-sensitive optical spectroscopy [25,26,27,28,29,30], biological and chemical sensors [31,32], nanofluidic devices [33], nanoreactors [34], biotechnology and biomedicine [35,36,37,38,39], or environmental decontamination [40]. They also frequently serve as templates for nanotechnology, e.g., for the synthesis of highly uniform rod-like or planar porous nanomaterials that cannot be achieved directly by the electrochemical method (readers who need a quick familiarization with the morphology and composition of PAA templates that have uniform cylindrical pores may turn to Figure S1 in the Supplementary Materials file) [1,41,42,43]. Moreover, the fabrication of self-organized PAA with controllable size and arrangement of pores in many instances offers a cost-effective alternative to conventional nanolitographic approaches. Thus, the new demands in different frontier areas of science and technology have noticeably changed the conversation in the contemporary preparative electrochemistry within the recent decades. New ambitious objectives have inspired scientists for fundamental and application-oriented research that would enable the rational design and synthesis of novel PAA types with tailored morphological and unique functional properties [44,45]. Dealing with such self-organizing patterns often pushes chemists and material engineers with classical academic background to a new and unfamiliar territory. It should be reminded that the classical electrochemical systems are typically characterized by a low applied voltage, moderate concentrations of electro-active substances (down to 10^−3^–10^−5^ M), and a high excess of non-reactive supporting (auxiliary) electrolyte. In such systems, the electrical current arises predominantly due to the migration of ions under the applied potential gradient (the relative contribution from diffusion can be often neglected) [6]. They demonstrate invariable electrical conductivity *σ* (or specific resistance *ρ*) upon arising fluid motion and a linear response of the electrical current to the applied potential difference (or, in other words, obey Ohm’s law). In self-organizing dissipative electrochemical systems, on the contrary, the role of the fluid motion, as well as its impact on the electrical conductivity of the electrolyte solution and the morphology of deposited materials noticeably increases. The solution-specific resistance *ρ* does not relate the arising current linearly to the applied voltage in the case with nonlinear dynamic electrochemical systems any more. Instead, complex “current-time” (*I*–*t*, or *j*–*t*) and “current–applied voltage” (*j*–∆*U*) curves of different shapes can be observed. Analysis and comparison of these curves allows for valuable conclusions about the established self-organization regime, the suitability of a particular selected electrolyte, possible improvements of the experimental parameters (required changes in applied voltage, concentration, pH, adding substances for viscosity modulation, etc.), and it also helps comprehend the prevailing transport mechanisms or even provides new insights into the driving forces causing the self-ordering under certain conditions. In such self-organizing electrochemical systems (e.g., dealing with electrolysis or electrodeposition), the electroconvective ion transport mechanism prevails and helps exceed the diffusive transport limitations. It has been demonstrated that increasing the applied voltage ∆*U* above a certain critical limit gives rise to convective ion transport, which helps to overcome the achieved transport saturation and leads to so-called “overlimiting current” [46]. Along with this, convection (which can be defined as a macroscopic collective flow of fluid particles) also frequently serves as the source of self-organization in such systems [6].

The present work focuses on the progress made toward the elucidation of self-ordered anodic alumina structures’ formation mechanism. First, some basic principles of dissipative self-organization are briefly discussed in Section 2 (such as the “constant energy flow” principle which dictates the design rules for open anodizing cells). Then, the role of the various contributing factors, including temperature regime, types of electrolytes, pH, anode material properties, and the nature of produced oxide–hydroxide is thoroughly analyzed in Section 3, Section 4 and Section 5. In particular, Section 5 includes sections on the mechanism of formation of polycrystalline interpore walls and on the incorporation and distribution of electrolyte impurities in PAA through the anion-exchange process. After that, the focus is directed toward the evolution of the theories explaining PAA formation that have been developed over recent decades. The part comprising Section 6 and Section 7 begins with the analysis and discussion of the early models where the self-organization was governed by the conventional principles of classical low-voltage electrochemistry, and then, it proceeds to the more recent attempts of reconsideration using some basic concepts of chaos and self-organization theory and elements of synergetics [47,48,49].

The primary purpose was to provide a comprehensive up-to-date review that would transfer the accumulated systematic knowledge of this topic in a concise format, as well as logically unite extensive scattered empirical observations and identify common principles. The acceptance of numerous experimental facts, along with found relationships between them, has led to a growing consensus in the research community that some types of self-organizing electroconvective phenomena are partially responsible for the PAA growth mechanism. Therefore, particular attention will be given in this paper to the convective models. The author hopes that this work may be useful for those scientists who want to familiarize themselves with the self-ordered anodic alumina research and for those who have been already actively involved into the research in this area and would like to reconsider their old practical problems from new perspectives.

## 2. Electrochemical Cell Construction According to Some Fundamental Principles of Dissipative Thermodynamics

The nonlinearity and the presence of feedback loops discussed in the previous section belong to the kinetic requirements for the occurrence of self-organization. However, there is also a necessary thermodynamic factor to be taken into account when designing and constructing a properly functioning two-electrode electrochemical cell for PAA synthesis.

At first glance, self-ordering must contradict the second law of thermodynamics because it implies the decrease in entropy. The second law of thermodynamics states that only those processes can spontaneously occur in an isolated system that lead to the increase in the system’s disorder, that is, to the increase in entropy (since the increment in randomness is characterized by the entropy factor *T*∆*S*, where *T* is the absolute temperature, and ∆*S* is the change in entropy). However, we should remember that self-organization always occurs in open systems with heat elimination. Thus, the atmosphere should also be considered as a part of our system. The dissipation of energy in the form of heat results in the increase in environmental entropy so that the total entropy also rises. Therefore, there is no contradiction to the second law of thermodynamics. That is to say, a self-organization process must be always accompanied by a parallel dissipative process overcompensating the decrease in entropy due to pattern formation [7]. This principle is generally implemented during the construction of a conventional electrochemical cell for the synthesis of well-ordered PAA. As a rule, such systems are equipped with a powerful thermostat and are vigorously agitated with a mixer or a magnetic stir bar to facilitate heat elimination from the working electrode.

In order to maintain the long-term stability of a dissipative self-organization process, the constant flow of energy through an open system must be supported. For achieving a stable anodizing regime, the input of energy into the electrochemical cell is performed through the application of the electric field, which gives rise to the ionic current. A certain part of this input energy is converted into the useful work for orderliness creation on the anode surface. The output of energy takes place through the active cooling using an external device or through direct heat exchange (the latter is true if the electrolytic bath temperature exceeds the ambient temperature) and dissipation into the atmosphere.

In some cases of extremely intense heat generation at the anode (e.g., while conducting experiments in H_3_PO_4_ electrolyte solutions at a typically high applied voltage between 160 and 195 V), powerful refrigerating circulators with high cooling capacity, as well as customized electrochemical cells with constant reconditioning of the electrolyte solution are employed to control the temperature and to prevent the so-called “aluminum burning effect” (that is, local concentration of the electrical current and accelerated dissolution of metallic substrate, which damages the anodic layer and disturbs the pore ordering) [8,50,51]. In addition, the anode surface area where the heat is produced is kept as small as possible (typically from several cm^2^ down to 0.5 cm^2^) [50,52]. Customized thermally isolated electrochemical cells usually have a small volume (down to 1 cm^3^) and incorporate a copper plate with good heat conductivity serving as the anode holder (Al anodes are mounted on this plate and exposed to the acid solution). During anodizing, the electrolyte can be either stirred or recycled using a pump system that pumps a large excess of cooled solution (typically at 0 °C) through the cell [8].

Until now, we have considered anodizing experiments in which thermostatic devices keep the bath temperature lower than the ambient temperature. However, successful experiments where self-ordering on electrochemically etched aluminum surface occurs at a temperature higher than the ambient have been also reported. For instance, Stępniowski et al. have demonstrated that controlled PAA growth can be achieved using 0.3 M oxalic acid solution without the critical “aluminum burning” effect, even at temperatures as high as 35–50 °C [53]. Later, hierarchical self-ordered textures with cell size variation from the nanometer to sub-millimeter range were demonstrated upon aluminum anodizing using a 0.3 M H_2_SO_4_ solution at 40 °C [54]. Thus, it would be logical to ask whether such processes in which a thermostat is set to increase the bath temperature above the temperature of the environment (instead of heat elimination and cooling) contradict the previously discussed thermodynamic principle which implies a constant energy flow through the system. It should be mentioned that the heat exchange is a function of the temperature gradient. Therefore, an increase in temperature difference between a thermostatically controlled open bath and the atmosphere also increases the heat dissipation from the bath. Thus, the basic principle is preserved: pattern formation is still assisted by a dissipative process.

Successful self-ordered anodic structure synthesis under conditions close to adiabatic (that is, where the energy exchange between the electrochemical cell and the ambient atmosphere is not allowed) would constitute a valid refutation of the “constant energy flow” principle. In practice, this would mean conducting an experiment in an insulated vessel where all generated heat is accumulated and stored during the entire electrosynthesis process. However, to the extent of the author’s knowledge, there are no reports about such successful experiments at the time of this writing (some typical examples of the electrochemical cells employed for PAA synthesis are demonstrated in Figure S2 in the Supplementary Materials document).

## 3. General Features and Classification of Suitable Electrolyte Solutions

In order to improve the versatility of the anodizing method and to create PAA honeycombs with customized morphologies for diverse practical applications, understanding the influence of different experimental parameters on the self-ordering process is of crucial importance. One of the most essential parameters that opens a facile access to a broad spectrum of pore and cell dimensions is the employed electrolyte type. It should be stressed that water is the only electro-active substance during the electrochemical generation of PAA. Dissolved electrolyte species (that is, acid anions) are normally not consumed in the RedOx processes at the anode, and their concentrations remain unchanged throughout the entire aluminum anodizing process (here, we neglect the loss of little amounts of electrolyte due to its incorporation into alumina as anionic admixture [55,56,57]). Water molecules are electro-neutral, and the concentration of OH^−^ ions that can migrate toward the positively charged anode is less than 10^−7^ mol L^−1^ (here, we must bear in mind that [H^+^][OH^−^] = *K*_w_ ≈ 10^−14^ mol^2^ L^−2^ at a typical anodizing temperature, while experiments are conducted in acidic medium, i.e., [H^+^] >> [OH^−^]). Hence, the presence of negatively charged electrolyte anions (i.e., an auxiliary substance that remains non-reactive in a wide range of applied voltages) is necessary for supporting sufficiently high migration current and for evoking the accompanying flow of entrained water (the electro-active component) through the PAA layer toward the metallic working electrode where the RedOx reactions occur. Hydrated anions migrate toward the aluminum substrate surface where water is oxidized and yields mixed oxide–hydroxide (boehmite *γ*-AlOOH):

Al + 2H_2_O → Al(O)OH +3H^+^ + 3e^−^. (1)

The water content in 1 L of a diluted electrolyte solution (this parameter can be also defined as “concentration of water in aqueous solution” and expressed in mol L^−1^) within the typical anodizing temperature range is about 55.5 mol (here, it is assumed that the volume changes caused by the dissolution of moderate amounts of electrolytes can be safely neglected), whereas concentrations of supporting electrolytes rarely exceed 0.1–0.3 M in most conventional compositions. In some exceptional cases, the supporting electrolyte concentration can be as high as 2.25 M (which corresponds to 20 wt % of H_2_SO_4_) [58]. Even more concentrated 8–9.4 M solutions were employed for the experiments performed at extraordinarily high temperatures (40–60 °C) [59]. However, even under such rather “exotic” experimental conditions, the amount of water (the reactive component of the mixture) still significantly exceeds the amounts of added supporting electrolytes. It is worth mentioning here that solutions used in the classical electrodeposition method are, on the contrary, typically characterized by a comparatively low concentration of the electro-active substance (from 10^−5^ to 10^−3^ M) and a significant excess of the added inert auxiliary electrolyte (usually between 0.1 and 0.5 M). This distinction constitutes a key difference between the solutions used for the synthesis of self-organized PAA and solutions prepared for the classical low-voltage electrochemical systems.

All electrolytes that have been to date successfully employed for PAA fabrication are often roughly categorized by researchers as inorganic and organic acids [60]. More specifically, they can be also grouped into the following three main categories:

- Organic carboxylic acids. As a rule, these are polyprotic acids [1,9,17,60,61,62,63,64,65], but the applicability of monoprotic acids has been also demonstrated on the example of formic acid HCOOH [16];
- Oxoacids with a chalcogen central atom (sulfuric or selenic) [2,66,67,68,69]. The derivatives with a substituted oxo-group (e.g., thiosulfuric) or peroxoacids are excluded because they are instable either thermally, or in diluted water solutions, or can be even explosive;
- Oxoacids containing phosphorus (such as phosphoric or phosphonic) [8,50,70]. Experiments with etidronic acid CH_3_C(OH)[PO(OH)_2_]_2_ have demonstrated that this range can be also widened due to the electrolytes that have a structural relationship to phosphoric acid [71,72]. However, this group cannot so far be expanded to include acids containing other pnictogens (e.g., nitric acid [73]) because their ability to produce cellular alumina nanostructures has not been experimentally confirmed.

Apart from these three groups, chromic acid H_2_CrO_4_ still remains beyond the classification. Thus, it can be also included into a broader class of simply inorganic electrolytes.

Table 1 lists some examples of suitable electrolyte solutions as well as the appropriate anodizing conditions and morphological parameters of the resulting PAA structures.

The required solution properties for the optimized stable PAA growth (pH, electric conductivity, viscosity, etc.) can be also achieved by mixing different types of electrolytes, e.g., inorganic sulfuric and organic oxalic acids [77,79]. Thus, it does not necessarily have to be a single-solute composition, and the specific chemical nature of electrolyte as such seems to have only a minor importance for pore ordering from a dynamic perspective (when compared to other underlying factors, such as the magnitudes of dissociation constants, ionic mobility and so on, although they can be, of course, ultimately also considered as related to the chemical nature). The electrolyte solution may also contain different other non-reactive additives (e.g., salts, monoatomic and polyatomic alcohols or polymers) for the modulation of fundamentally important electrochemical and hydrodynamic properties (for instance, such characteristics as dielectric constants, viscosity, and ion velocities may be targeted) [10,52,80,81,82,83,84]. Some other parameters of electrolytes frequently varied by experimentalists when searching for new PAA fabrication conditions (concentration, temperature, etc.) [85] also influence the key dynamic properties of fluids (for example, the dependence between viscosity and temperature is known to be established by Stokes–Einstein relation).

**Figure 4:**
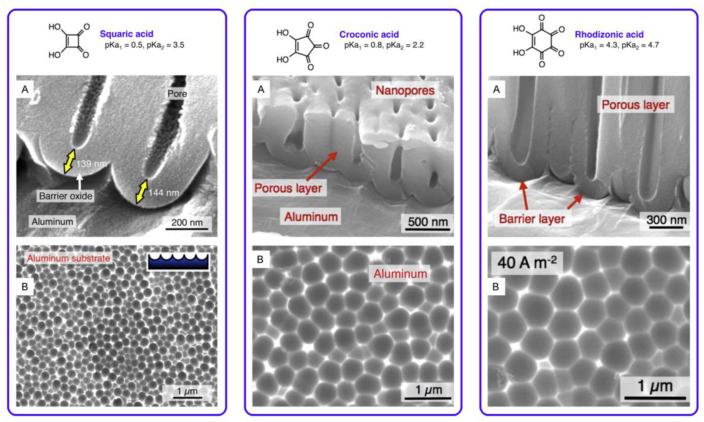
Examples of PAA laminas synthesized using cyclic oxocarbon acids (Kikuchi et al. [60,86]). From left to right: SEM images demonstrating the morphological features of PAA formed in squaric acid (via potentiostatic anodizing at 100–120 V), croconic acid, and rhodizonic acid solutions (via galvanostatic anodizing at 20–40 A m^−2^). In each case, cross-sectional views of PAA are labeled as (**A**), and the micrographs showing arrays of shallow dimples remaining on the metal surface after the selective removal of PAA using a CrO_3_/H_3_PO_4_ solution are labeled as (**B**). Adapted with permissions. Copyright 2014 Elsevier.

Still, it must be noted that all acids that have a confirmed ability to facilitate PAA formation have one important characteristic in common also from the chemical standpoint: they all contain oxygen atoms in their composition. As discussed previously in this section, acid anions play the role of the auxiliary electrolyte for the transportation of coordinated and entrained water through the PAA to the metallic substrate. The ability of acid residuals to transport water in their outer coordination spheres substantially depends on the contained oxygen atoms due to the increased number of hydrogen bonds. Indeed, all the acids that are able to support the PAA growth are oxoacids but not hydrogen acids such as HCl. One prominent example that confirms the importance of such water coordination is the successful use of cyclic oxocarbon acids: several oxygen atoms act as coordination sites for water, and the large size of bulky carbocyclic molecules reduces the steric difficulties during the ligand arrangement (see Figure 4) [60,86].

Of course, the dissociation constants *K*_a_ are also among the most important parameters of acid electrolytes. For instance, *K*_a_ largely determines the ionic conductivity of a solution. It needs to be sufficiently high to support compatible values of the electrical current density *j* during the electrochemical process. The value of *j* is also associated with the velocity of RedOx reactions at the interface between the anode and the liquid solution (such RedOx reactions are necessary because these two parts of the electrical circuit are characterized by different types of conductivity, electronic and ionic, respectively). Thus, the value of *j* that results from a combination of these two factors (i.e., the ionic conductivity of a solution and the velocity of charge carriers conversion at the solution/electrode interface due to RedOx reactions) also reflects the efficiency of a particular electrolyte in flattening natural defects on rough aluminum surface (a slow process akin to electropolishing) followed by etching of the well-ordered hexagonal concaves.

**Figure 5:**
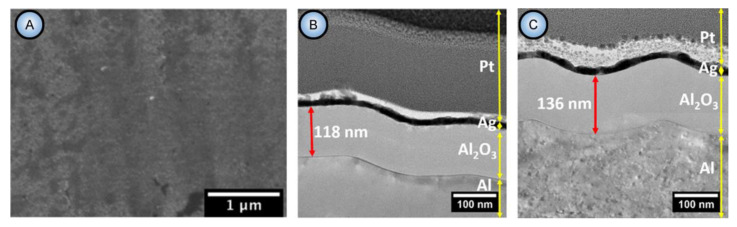
An SEM image (**A**) and a cross-sectional TEM (FIB-TEM) image (**B**) of a barrier-type anodic alumina layer prepared by anodizing in 0.3 M periodic acid (H_5_JO_6_) solution (pH = 1.1) at a constant applied voltage of 80 V. For comparison, an FIB-TEM image of a barrier-type film prepared in a citrate buffer solution (pH = 5.8) at 100 V is shown in (**C**). Reproduced with permission from [88]. Copyright 2020 American Chemical Society.

It was also long noted that electrolytes in which the formed alumina is slightly soluble (pH < 4) can produce PAA, whereas those in which alumina is insoluble (e.g., boric acid, ammonium borate or tartrate, neutral or slightly acidic solutions that have pH values around 5–7) produce non-porous (so-called “barrier type”) layers [87]. However, there are some evident exceptions to this rule. For example, 0.3 M solution of periodic acid H_5_JO_6_ has a pH value close to 1.1, and 1.0 M solution has pH < 1.0 (measurements of pH values close to 1.0 usually involve some practical difficulties because a glass electrode shows noticeably overestimated results in this so-called “acidic error range”). Although periodic acid is a weak acid with positive p*K*_a_ in Brønsted’s terminology (p*K*_a1_ = 1.6, p*K*_a2_ = 7.0 at 25 °C), the acids with 0 < p*K*_a_ < 4.5 are sometimes also considered as moderately strong (for comparison, widely used for PAA synthesis oxalic acid has p*K*_a1_ = 1.25, p*K*_a2_ = 4.27). Given these considerations, such oxoacid with the central halogen atom should be able to assist PAA formation. Nevertheless, it has been recently demonstrated that both 0.3 M and 1.0 M H_5_JO_6_ solutions yield barrier-type alumina layers at temperatures 0…1 °C and ∆*U* between 80 and 200 V (Figure 5) [88]. Thus, the usefulness of every new electrolyte has to be tested experimentally because different existing theoretical criteria are still not completely reliable for making predictions.

Since dissociation constants *K*_a_ are functions of temperature, variation of the solution temperature should change the degree of dissociation of auxiliary electrolytes and consequently also influence (although possibly only very slightly) the necessary parameters for a stable PAA growth, such as pH, electrical conductivity, etc. The dissociation of acids in water solution is an exothermic process due to the comparatively high enthalpy of hydration of the proton. Therefore, lowering the solution temperature must shift the equilibrium toward the dissociated forms according to Le Chatelier’s principle. That is to say, the new dissociation constants and the corresponding pH values calculated for an experiment conducted at the typical anodizing temperature of 0 °C should differ (though possibly only moderately) from the reference data usually determined at 25 °C. In order to understand the significance of this effect for a specific case of anodizing in a refrigerated solution, it can be quantified by performing calculations using the van’t Hoff equation. Below, such calculations are demonstrated on examples of two of the most frequently used electrolytes—namely, 0.3 M sulfuric and 0.3 M oxalic acids.

**Calculations using van’t Hoff equation performed for 0.3 M sulfuric acid solution.** The dissociation constants of sulfuric acid at 25 °C (298.15 K) are *K*_a1_ = 1 × 10^3^ (p*K*_a1_ = −3; H_2_SO_4_ is a very strong acid and p*K*_a1_ << 0) and *K*_a2_ = 1.15 × 10^−2^ (p*K*_a2_ = 1.94). Lower temperatures will promote the dissociation (the constants *K*_a_ will increase) because of the well-known exothermic effect of H_2_SO_4_ dissolution in water. The enthalpy of dissociation ∆_dis_*H*°_m_ (m = “molar”) can be found as the difference of the enthalpies of formation ∆_f_*H*° of sulfuric acid and all corresponding ions in water at the infinite dilution. After that, the dissociation constants can be adjusted to 0 °C (273.15 K) using the obtained ∆_dis_*H*°_m_ of both dissociation stages and the van’t Hoff equation:

(2) $$\ln\left(\frac{K_{a}\left(298.15\right)}{K_{a}\left(273.15\right)}\right)=-\frac{\Delta_{\mathrm{dis}}H{{}^{\circ}}_{m}}{R}\left(\frac{1}{298.15}-\frac{1}{273.15}\right)$$

where ∆_dis_*H*°_m_ is expressed in J mol^−1^, and *R* = 8.314 J mol^−1^ K^−1^ (*R* is the universal gas constant; note that the expression on the right side of the equation yields a dimensionless number, which meets the requirements for the logarithmic expression on the left).

The enthalpies of formation of liquid (l) H_2_SO_4_ and the hydrated ions at infinite dilution are listed below:

H_2_SO_4_ (l): ∆_f_*H*°(298.15, l) = −814.0 kJ mol^−1^

H^+^: ∆_f_*H*°(298.15, aq) = 0 kJ mol^−1^ (is set equal to zero by convention [89])

HSO_4_^−^: ∆_f_*H*°(298.15, aq) = −889.2 kJ mol^−1^

SO_4_^2−^: ∆_f_*H*°(298.15, aq) = −911.0 kJ mol^−1^

Thus, the enthalpies of dissociation can be expressed as follows:

First stage (H_2_SO_4_ ↔ H^+^ + HSO_4_^−^)

∆_dis_*H*°_m_ = −889.2 − (−814.0) = −75.2 kJ mol^−1^

Second stage (HSO_4_^−^ ↔ H^+^ + SO_4_^2−^)

∆_dis_*H*°_m_ = −911.0 − (−889.2) = −21.8 kJ mol^−1^.

The corresponding dissociation constants at 0 °C (273.15 K) calculated using the Equation (2) are *K*_a1_ = 1.5 × 10^4^ (p*K*_a1_ = −4.2) and *K*_a2_ = 2.5 × 10^−2^ (p*K*_a1_ = 1.6).

Now, we can calculate and compare the pH values of 0.3 M H_2_SO_4_ solutions at two different temperatures. The methods of calculating H^+^ concentrations in solutions of strong and weak electrolytes can be found elsewhere and are not discussed in detail in this work. We obtain pH = 0.51 for 25 °C and pH = 0.49 for 0 °C. Thus, there is only a negligible difference in pH values for 0.3 M H_2_SO_4_ solution due to such temperature changes.

**Calculations using van’t Hoff equation performed for 0.3 M oxalic acid solution.** The dissociation constants at 25 °C (298.15 K) and the corresponding calculated pH value are: *K*_a1_ = 5.6 × 10^−2^ (p*K*_a1_ = 1.25), *K*_a2_ = 5.4 × 10^−5^ (p*K*_a1_ = 4.27), pH = 0.98.

The enthalpies of formation of ions at the infinite dilution are as follows:

HC_2_O_4_^−^: ∆_f_*H*°(298.15 aq) = −818.18 kJ mol^−1^

H^+^: ∆_f_*H*°(298.15 aq) = 0 kJ mol^−1^

H_2_C_2_O_4_: ∆_f_*H*°(298.15 aq, non-dissociated) = −815.04 kJ mol^−1^.

The enthalpy of dissociation for the first stage of deprotonation (H_2_C_2_O_4_ ↔ HC_2_O_4_^−^ + H^+^) equals ∆_dis_*H*°_m_(1 stage) = −818.18 − (−815.04) = −3.14 kJ mol^−1^. Now, using Equation (2), the first dissociation constant *K*_a1_ can be adjusted to 0 °C (273.15 K):

$$\ln\left(\frac{0.056}{K_{a1}\left(273.15\right)}\right)=-\frac{-3140}{8.314}\left(\frac{1}{298.15}-\frac{1}{273.15}\right);\;K_{a1}\left(273.15\right)=0.063.$$

Thus, *K*_a1_ at 0 °C equals 6.3 × 10^−2^, and p*K*_a1_ = 1.2. For oxalic acid, the second dissociation constant *K*_a2_ can be calculated by an alternative method without using Equation (2). The variation of *K*_a2_ with absolute temperature, *T*, is represented by [90].

$$pK_{a2}=\frac{1423.8}{T}-6.5007+0.020095T$$

Using this empirical relationship, we can obtain p*K*_a2_(273.15) = 4.2 and *K*_a2_(273.15) = 6.3 × 10^−5^. Consequently, the pH value for a 0.3 M oxalic acid solution is equal to 0.96, and thus, the change upon the decrease in temperature from 25 to 0 °C is also rather negligible.

As we can see from both foregoing examples, the effect of low temperature on the dissociation of acids and the changes of pH is insignificant. Thus, low temperature seems to make its major impact on the rates of the involved electrochemical processes, dynamic viscosity, ionic mobility, etc.

Performing analogous calculations using the van’t Hoff equation for H_2_SO_4_ at the elevated temperature of 60 °C (or *T* = 333.15 K) shows that dissociation is suppressed: *K*_a1_(333.15) = 40 (p*K*_a1_(333.15) = −1.6), *K*_a2_(333.15) = 4.5 × 10^−3^ (p*K*_a2_(333.15) = 2.34). These new constants yield pH = 0.52 for a 0.3 M H_2_SO_4_ solution at 60 °C, which is still close to the pH value at 25 °C (regardless of the 25-fold decrease in *K*_a1_). Thus, the successful utilization of sulfuric acid solutions at extraordinarily high 40–60 °C anodizing temperatures without observing the critical “burning” effect (i.e., the intense local dissolution of Al) still does not lend itself to a simple explanation by using this calculation approach. Nevertheless, it must be noted that the high 9.4 M concentration of H_2_SO_4_ employed by Masuda et al. in the high-temperature experiments practically means mixing pure acid with water at a volume ratio of 1:1 [59]. Under such conditions, the considerations that we normally apply to moderately concentrated electrolyte solutions may be no more applicable (even the amount of water may be not sufficient for a complete solvation of sulfate ions).

Another circumstance that may also add complexity to the process is that anodizing experiments are conducted in water as a solvent. Water plays a crucial role in dynamics and electrode reactions. It will be no exaggeration to say that H_2_O is one of the simplest and one of the most complex substances in nature at the same time. The existence of aggregates composed of different numbers of water molecules (which can be also considered as short-range ordered fragments of different modifications of ice), complex behavior of its physical properties depending on temperature, as well as self-ionization make such water-based systems rather complicated (e.g., the self-ionization constant *K*_w_ of boiling water is approximately equal to 10^−12^, which yields the neutral pH value equal to 6, but not 7 as we used to consider for 25 °C).

## 4. The Role of Anode Material Morphology and Microstructure

There are a number of useful functional properties of PAA (primarily optical and photonic), which are determined by the regularity in hexagonal pore arrangement and formation of a two-dimensional lattice-type structure on nanometer or sub-micrometer length scale [21,30]. Thus, one of the most important morphological hallmarks of PAA frequently analyzed in the literature is the average size of the long-range ordered domains, which are comprised of six-fold coordinated cells [30,81,91,92,93]. It should be mentioned that the ideal defect-free ordering of a spontaneously formed PAA structure (that is, formed without the help of artificial pre-structuring methods where pore nucleation sites are defined via lithography or nano-embossing [69,94]) has never been achieved on the entire anodized metallic surface, and the dimensions of the best reported self-ordered domains are typically limited to several micrometers. Therefore, understanding the main controlling factors for the formation of such coherent domains is one of clear priorities in contemporary PAA research. Some researchers consider that the improvement of the hexagonal pore arrangement is related to the crystallographic parameters and size and orientation of grains in the metallic substrate. In this section, the main progress that has been made along this line of investigation will be discussed.

### 4.1. Search for a Relationship between the Nanopore Arrangement in PAA and Morphology, Microstructure, and Orientation of Al Crystallites

Beck and Petrikowski searched for correlation between the regularity in nanopore arrangement obtained in a two-step anodizing process and Al substrate preparation procedures—namely, mechanical polishing and annealing [95]. In this study, the influence of the employed pre-treatment methods onto Al microstructure was first of all implied. Metallic substrates with different purity (99.5–99.999%) as well as AlMg1 alloy were investigated. The main findings of this study are summarized as follows:

- The most important factor contributing to orderliness enhancement in PAA is the implementation of the two-step anodizing method (first introduced by Masuda and Fukuda [1]) and the corresponding pre-texturing of the Al surface before the main oxidation step;
- PAA formed on a rough (rolled) Al surface is mainly disordered, and mechanical polishing is able to improve the regularity only to a minor degree;
- Neither the grain size and shape nor the dislocations density in a polycrystalline substrate can significantly affect the nanopore regularity in PAA. The authors have been inclined to the view that the pore ordering is influenced by the orientation of aluminum crystals and the fraction of crystals with the cube texture component (that is, with (100) facet exposed to the surface);
- The authors have also detected a correlation between the performed annealing procedure (typically up to 500 °C, except for the magnesium-containing alloy, which was heated up to 410 °C) and the orientation effects in the FCC (face-centered cubic) aluminum phase (a large fraction of enlarged crystallites with (100) facets oriented toward the surface);
- In the case with highly pure Al substrates (99.999%), annealing and recrystallization do not improve the pore arrangement (the same good pore regularity was observed on both the annealed and untreated samples). However, the pore regularity does improve after annealing in the case with lower substrate material purity (≤99.5%). The average ordered domain size in PAA formed on both mechanically polished and unpolished 99.5% Al foils has increased from ≤200 × 200 nm to about 500 × 500 nm after their thermal treatment (however, these figures still remain below the 1 × 1 µm domain sizes observed on rough and polished 99.999% Al substrates).

**Figure 6:**
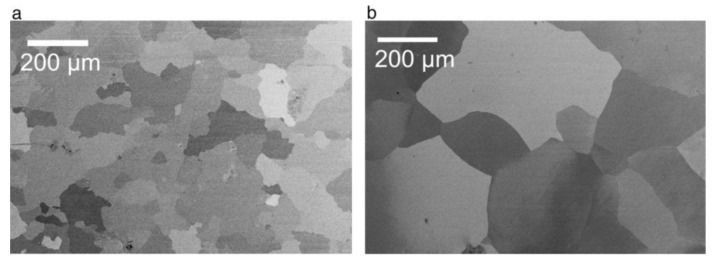
SEM micrographs of highly pure 99.999% Al sheets prior to thermal treatment (**a**) and after annealing at 500 °C for 3 days (**b**). Reproduced with permission from [95]. Copyright 2008 Elsevier.

The latter two observations deserve more detailed consideration. In general, all the observed metamorphoses in aluminum substrates upon annealing are based on the commonly known mechanisms of the surface energy reduction in solids. The enlargement of Al crystallites results from their sintering, which is the merging of smaller crystallites into larger ones with reduced overall surface area. The product of sintering is a polycrystalline material where individual crystallites are reshaped and densely packed without gaps in between. Significantly enlarged grains with a lower density of grain boundaries were also observed in the study reported by Beck and Petrikowski after the thermal treatment of highly pure Al sheets (Figure 6). Sintering generally becomes important when a material is heated up to approximately 70% of its melting point. This “thumb rule” is in full agreement with the study under consideration because the samples were annealed at 500 °C while pure Al melts at 660.3 °C [96].

**Figure 7:**
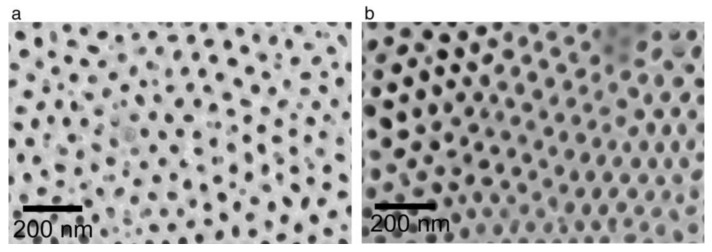
Different degrees of hexagonal pore ordering achieved in a two-step anodizing process using untreated 99.5% Al sheets (**a**) and the same sheets prepared by thermal treatment at 500 °C for 3 days (**b**). Reproduced with permission from [95]. Copyright 2008 Elsevier.

Another well-known mechanism of surface energy reduction perfectly explains why the annealing of highly pure Al substrates (99.999%) has almost no visible impact on the pore regularity, whereas PAA layers formed on Al foils of lower purity (≤99.5%) demonstrate a considerable improvement of orderliness after substrate annealing prior to anodizing (see Figure 7). This presumably happens due to the phase segregation and enrichment of impurity on the surface of small crystallites. That is to say, the grains in Al tend to form a nearly perfect crystal structure and repel admixtures and defects from the interior to the surface (a commonly known effect for nanostructured materials that creates difficulties in their doping) [96]. Then, the admixture concentrated on the surface of annealed metal foils is removed after the first (preliminary) oxidation in a two-step anodizing process (in the study of Beck and Petrikowski, PAA layers obtained after the first anodizing step had a thickness of approximately 35 µm, which means that given the known volume expansion coefficient during the conversion of Al to PAA ≈ 1.2–1.3 [92], a 27–29 µm thick contaminated surface layer of metallic substrate is removed after the first stage of anodizing). Therefore, the quality of PAA layers is very likely improved after contaminated substrate annealing and pre-oxidation (see Figure 7a,b) not because of the changed grain size or microstructure but primarily because the working electrode material gets purified from the initially contained disturbing admixture.

In a follow-up study, Beck and Bretzler have considered the thermodynamic aspects of pore etching on (111), (110), and (100) facets of Al single crystals, which are characterized by different values of surface energy [97]. The surface energy *γ* on various facets of a crystallite can be roughly estimated using the following equation:

(3) $$\gamma =\frac{1}{2}N_{b}\varepsilon \rho_{a}$$

where *N*_b_ is equal to the number of bonds for each atom on a singular surface that appear broken in comparison with the atoms in the bulk, *ε* is the half-strength of the bond, and *ρ*_a_ is the surface atomic density. Based on the comparison of the atomic densities *ρ*_a_ on different Al facets, the authors have obtained the following sequence of systematically increased distributed surface energies: γ_(111)_ < γ_(100)_ < γ_(110)_. However, before we can proceed with the analysis of this work, one remark should be made. For an elemental crystal with FCC structure (which corresponds to the prevailing phase in aluminum, according to the preceding work of Beck and Petrikowski [95]), every surface atom on (100) facets has *N*_b_ = 4, on (110)—5, and on (111)—3. Accordingly, the surface energies calculated using Equation (3) are distributed as follows: γ_(111)_ < γ_(110)_ < γ_(100)_ (i.e., there is a systematic correlation between the surface energy and Miller indices; the interested readers are encouraged to refer to the cited monograph by G. Cao for more details on the calculation parameters) [96]. Thus, these computed parameters may also need further verification.

**Figure 8:**
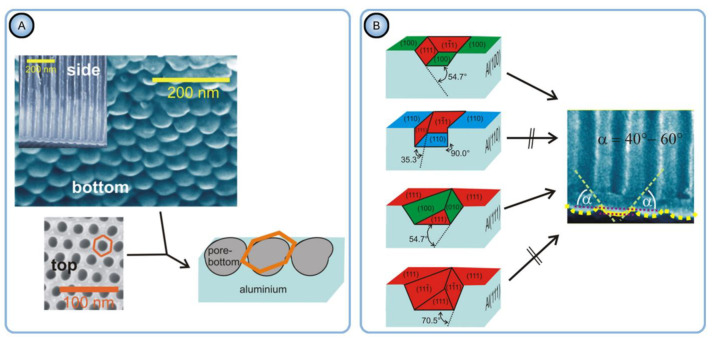
(**A**) SEM images demonstrating the bottom (sealed with the barrier layer), the lateral, and the top (porous) sides of PAA, as well as a schematic sketch of the hemispherical concaves on the Al surface underlying the pore bottoms; (**B**) A comparison of indentations on (111), (110), and (100) Al faces in terms of the resulting interface energy reduction and the correspondence of the angles between different planes to the microscopically observed pore tip geometry. Reproduced with permission from [97]. Copyright 2011 Elsevier.

The X-ray diffraction analysis in Ref [97] has confirmed the X-ray amorphous nature of the grown PAA laminas, and thus, the orientation of nanopores due to the epitaxial growth mechanism could be safely excluded from consideration. However, the authors found the nanopore arrangement on the (100) Al facet to be the most well-organized, and they suggested an explanation based on the analysis of the total Gibbs energy change ∆*G* in the course of the reactions accompanying the PAA growth. In simplistic terms, the total ∆*G* is made up of two components—namely, the Gibbs energy change corresponding to the occurring chemical processes (oxidation of Al and dissolution of PAA), and the overall change of the surface energy. The latter is comprised of the specific surface/interface energies *γ_i_* (per unit area) multiplied by the increments/decrements of the corresponding areas of phase boundaries *dA_i_*. Since a larger negative value of ∆*G* (i.e., a larger driving force of a reaction) means a stronger shift of the equilibrium toward higher concentrations of the electrode reaction products (∆*_r_G*° = −*RT*ln*K*, where ∆*_r_G*° is the standard free Gibbs energy for a reaction, and *K* is the equilibrium constant), a reduction of the interface energy term during the formation of a nanotextured (“waved”) Al/PAA boundary should facilitate metal dissolution and etching of pores. After some thermodynamic and geometric analysis (symmetry of concave nanopore bottoms should match the angles between considered facets in Al crystals), the authors came to the conclusion that the etching of pores in form of hexagons is most favorable in terms of energy and configuration on (100) facets of aluminum, whereas the formation of such porous patterns on (110) or (111) facets leads either to prohibited nanopore geometry or to the increase in the interface energy (see Figure 8).

### 4.2. Applicability of the Concepts of Classical Thermodynamics to Evolution of Self-Ordered PAA Structures and Investigation of the Effect of Anodizing Time

**Figure 9:**
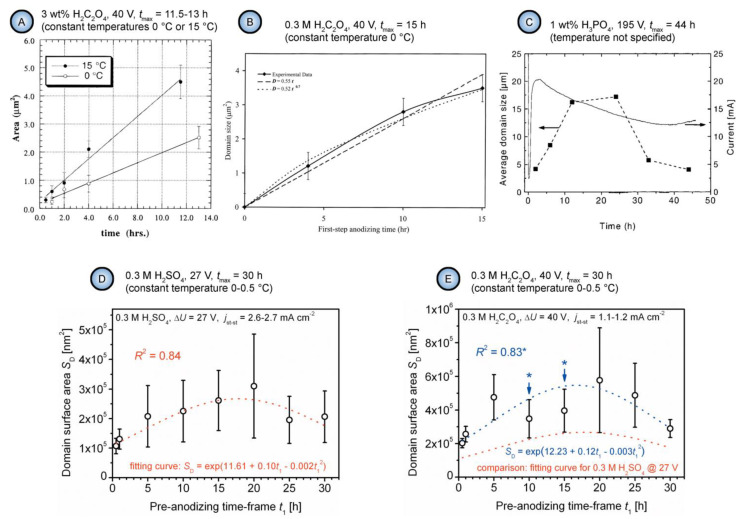
Several examples illustrating the time evolution of the average size of hexagonally ordered pore domains during long-term potentiostatic anodizing of aluminum in different electrolyte solutions. (**A**) A linear relationship reported by Li et al. for the 11.5–13 h long experiments performed in 3 wt % H_2_C_2_O_4_ at 40 V [91]; (**B**) The slope of the curve obtained for H_2_C_2_O_4_ solution visibly begins to decrease if the experiment duration is prolonged to 15 h [100]; (**C**) Anodizing over an extended 48 h time period using 1 wt % H_3_PO_4_ reveals the presence of a critical point in time after which the well-ordered domain size begins to systematically decrease [92]; (**D**,**E**) A combined study of H_2_C_2_O_4_ and H_2_SO_4_ electrolyte solutions has demonstrated that the evolutionary trend reverses after reaching some critical point in time (at approximately 20 h) irrespective of the employed type of electrolyte [30]. Reproduced with permissions. Copyright 1998, 2002 and 2020 American Chemical Society, 2006 Elsevier.

Consideration of the free Gibbs energy in the work of Beck and Bretzler [97] as a measure characterizing the natural tendency to form a hexagonal pattern on aluminum surface is an example of accurate application of the classical thermodynamics principles to explain irreversible processes leading to self-organization. Such explanations attract researchers by a comprehensible manner of mathematical formulation (and the author of this review is also no exception), but they often have limited capacity to reflect the entire picture and may also leave some experimental facts unexplained. While classical thermodynamics studies idealized systems that are either at equilibrium or in states close to equilibrium, dynamic self-organized (or dissipative) systems are usually far from an equilibrium state. Such systems are considered in the framework of so-called nonlinear thermodynamics of irreversible processes, which is an extension beyond the classical thermodynamics concentrated on the problems of non-equilibrium, chaos, and self-organization (Nobel Prizes in Chemistry awarded to Lars Onsager and Ilya Prigogine). A similar way to rationalize the hexagonal shape of PAA cells in a traditional way using the concept of thermodynamic stability can be also found, for example, in the very comprehensive and detailed monograph by Runge [98] (the creator of the pore formation model based on lateral growth of the oxide from preferential nuclei on Al surface driven by the mechanical stress and the electric field effects [99]). The mechanistic explanation contained in this book is that the outward uniform growth from the regularly spaced initial nuclei produces the naturally occurring geometric pattern with the most thermodynamic stability—the hexagon. However, such a classical thermodynamic approach must be very cautiously applied to non-equilibrium processes where orderliness is created from chaos, and entropy is locally decreased (the second law of thermodynamics does not favor such creation of order without specific reservations). An important distinction between the classical thermodynamics and the nonlinear thermodynamics of irreversible processes is that the latter includes time as a parameter. In this context, it is pertinent to quote the words of Ilya Prigogine (the creator of self-organization theory), who wrote: “Matter at equilibrium, with no arrow of time, is “blind”, but with the arrow of time it begins to see.” [47] By the “arrow of time”, he meant entropy because it is an essential element in the evolutionary description. Entropy deals specifically with irreversible, time-oriented processes (irreversible processes produce entropy, i.e., the overall entropy change of the system and its surroundings will be positive and non-zero). Although Prigogine extensively studied the microscopic world associated with thermodynamics, he also formulated the laws of nature that can be applied for macroscopic physics, chemistry, and biology (it is usually assumed that macroscopic properties acquire a physical meaning for objects containing at least 10^10^ atoms; i.e., such objects can be still relatively small, given the value of Avogadro’s number). He wrote, “The primordial role of the direction of time is evident in the processes we study at the macroscopic level, such as chemical reactions and transport processes” [47]. Going back to the study of self-organized PAA layers, there is also a number of important works investigating pore arrangement as a function of time [30,91,92,93,100]. While the galvanostatic anodizing regime is often employed to study the pore nucleation process, the long-term potentiostatic oxidation is usually the necessary condition to investigate the time evolution of the pore ordering. Li et al. conducted 11.5–13 h long anodizing experiments using 3 wt % oxalic acid electrolyte solution at a constant applied voltage of 40 V and reported a strict linear relationship between the average well-ordered domain size in PAA and anodizing duration (Figure 9A) [91]. Later, Ghorbani et al. performed a similar study in which the anodizing duration was increased to 15 h and reported a quasi-linear domain enlargement with a slow-down after approximately 10 h of the experiment (Figure 9B) [100]. Nielsch et al. experimented with 1 wt % H_3_PO_4_ solution within extended periods of time up to 48 h and reported a decrease in the ordered domain sizes after a certain critical time of anodic oxidation (Figure 9C) [92]. Thus, it was demonstrated in this study that the domain enlargement is not persistent, and there is an optimum anodizing duration after which the average domain size reaches its largest possible value. Another very recent combined research has shown that such pattern of domain size evolution is universal across different electrolyte types [30]. Similar to the study carried out by Nielsch et al. for phosphoric acid, two distinctive periods separated by a critical point in time were also determined for sulfuric and oxalic acid electrolytes (Figure 9D,E). These periods corresponded to the improvement of morphological order and the following decrease in order. The second period typically sets in after approximately 20 h of anodizing under mild conditions irrespective of the employed electrolyte type (in this work, variable time-frames between 0.5 and 30 h were tested). Thus, there is now sufficient accumulated experimental data indicating that the well-ordered domains undergo continuing resizing and reshaping in spite of the practically unchanged morphological and crystallographic parameters of metallic substrates throughout the entire anodizing process. This circumstance is another reason why skepticism regarding the relationship between these characteristics may arise.

### 4.3. The Pseudo-Epitaxial Growth of Self-Organized PAA Structures and the Introduction of the Concept of “In-Plane Orientational Order”

**Figure 10:**
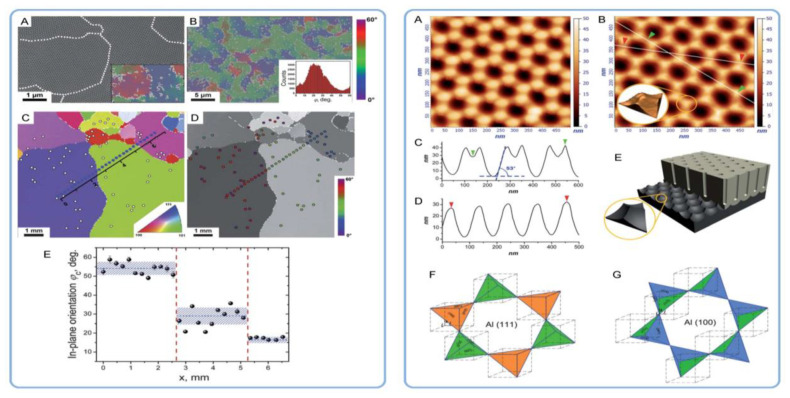
**Left side**: (**A**) An HRSEM image of PAA showing the boundaries between highly ordered pore domains (marked with dotted lines); (**B**) A lower magnification image in which pores are color-coded with respect to their average angles to the nearest neighbors; (**C**,**D**) Electron Backscatter Diffraction (EBSD) orientational distribution maps showing crystallographic orientations of Al grains, the colored points show the spots where SEM analysis of PAA was performed, the inset in (**C**) explains the color-coding of the crystallographic orientation of Al grains, the inset in (**D**) explains the color-coding of the detected average preferential pore orientation in PAA; (**E**) The spread of the preferential orientations of the porous structure measured along the blue dotted line in (**C**). **Right side**: (**A**,**B**) AFM images obtained from the bare metal surface after the removal of PAA; (**C**,**D**) AFM height profiles measured along the white lines in (**B**); (**E**) A schematic of the Al/PAA structure (the inset shows an individual pyramid between three neighboring pores, around which, according to Napolskii et al., the rotation of a PAA lattice is supposed to occur until the optimal orientation with respect to the crystallographic direction in Al is achieved); (**F**,**G**) Crystallographic orientation of the sides of pyramids for (111) and (100) single crystal Al substrates. Adapted with permission from [101]. Copyright 2012 The Royal Society of Chemistry.

Napolskii et al. have noted the large mismatch between the typical size of Al grains, as well as their microstructure, and geometric parameters of PAA lattices [101]. The most obvious fundamental discrepancy is that the defect-free pore ordering in PAA never extends beyond several micrometers, whereas crystallite dimensions in aluminum can be as large as several millimeters. Moreover, the self-ordering periodicity in PAA is approximately 10^3^ times larger than the lattice constants in Al crystallites. Along with the fact that PAA laminas have amorphous structure, this makes the oriented PAA growth due to crystallographic effects rather implausible. In an attempt to overcome these fundamental limitations, the authors have introduced the new concept of orientational correlations of pore positions (or “in-plane orientational order”) which can extend over macroscopic distances larger than several millimeters (see Figure 10). This parameter is based on the preferential orientation of the majority of individual domains over a large surface area. Thus, the new criterion can help to bypass the limitation of a single domain size in PAA and to bring the estimation results for the size of an ordered area in conformity with the distances between grain boundaries in an Al substrate. The authors have detected that the average in-plane orientation of the domains (which in fact compose a mosaic structure; see Figure 10B on the left-hand side) has one preferential direction within the boundaries of a single Al grain. The preferred ordering of rows of pores in PAA was also observed along the (011) crystallographic direction in Al. Such pseudo-epitaxial growth was reasoned by different etching rates in various crystallographic directions. The mechanism of orientational correlation was explained in the following way: the primary hexagonal pattern that determines the positions of etched pores on the Al substrate is rotated around a “vertical” pyramid at a place where three pores are adjacent (Figure 10E on the right-hand side), and this rotation proceeds until the moment when the PAA lattice is aligned with the crystallographic direction in Al, along which the pore etching results in the most efficient minimization of the surface energy. The proposed model predicted the ultimate ordering on Al(111) single crystals (compare with the conclusion made by Beck and Petrikowski, who determined that the best pore ordering occurs on the (100) facet of Al [95]).

In the follow-up study, Roslyakov et al. studied the correlation between the transverse and longitudinal orientations of cylindrical pores in PAA [102]. It has been demonstrated that domains with different in-plane orientation contain channels with different growth directions. The deviation of the channel growth directions from the direction perpendicular to the substrate was ascribed to the influence of the substrate crystal structure, but it did not exceed 1 degree.

### 4.4. Addressing the Density of Dislocations in Al Substrates as the Key Factor Influencing the Hexagonal Pore Ordering in PAA

**Figure 11:**
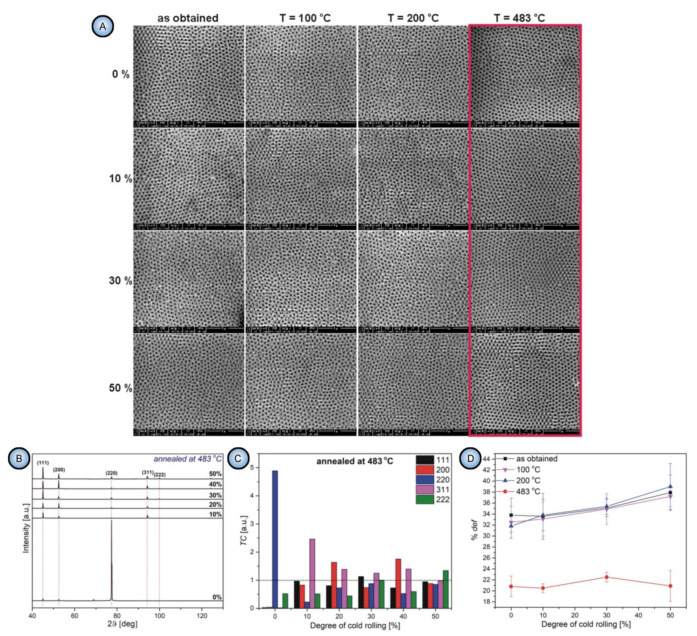
(**A**) SEM micrographs demonstrating different degrees of pore ordering in PAA laminas formed on Al substrates prepared by mechanical deformation (cold rolling to 10–50% thickness reduction) combined with thermal treatment (the temperatures of 100 and 200 °C correspond to the lattice strain relaxation, 483 °C is the temperature of Al recrystallization). (**B**) X-ray diffraction patterns of Al substrates annealed at the recrystallization temperature after different degrees of mechanical treatment. (**C**) A comparison of the preferential grain orientation in different Al samples annealed at 483 °C quantified by using the texture coefficient (TC) along every diffraction plane (a higher value of TC*_(hkl)_* > 1 indicates a higher preference of orientation along the corresponding plane). (**D**) The relationships between the degrees of mechanical deformation and the resulting percentages of defects in PAA pore arrangements obtained for Al substrates annealed at different temperatures. Adapted with permission from [103]. Copyright 2019 Elsevier.

In a recent extensive study, Norek and Szamyer investigated the effect of various degrees of mechanical treatment (cold rolling) and heating to different temperatures (corresponding to lattice strain relaxation at 100–200 °C and recrystallization of aluminum at 483 °C) on the percentage of defects in PAA hexagonal arrangement [103]. The best pore ordering was observed on Al substrates annealed at the recrystallization temperature irrespective of the preceding mechanical treatment, whereas heating to lower temperatures (influencing only the lattice strain parameter) was insufficient for the pore ordering improvement (see Figure 11A,D). On the substrates annealed below the recrystallization temperature, the observed number of defects in PAA was directly proportional to the degree of applied cold rolling (Figure 11D). The most important conclusions of this investigation are summarized as follows:

- All parameters of morphology (grain size, circularity, and elongation) and microstructure (lattice strain and surface texturing) are irrelevant to the pore formation mechanism. First, the substrates with only small percentages of mechanical deformation (0–10%) exhibited no changes in the aspect ratios of grains upon annealing at 483 °C. At the same time, they demonstrated a significant improvement of the ordered PAA structure. Second, the improvement of pore ordering upon such annealing appeared almost identical for the substrates that demonstrated a five-fold enlargement of grains and for the substrates where the grain size did not undergo such dramatic changes;
- The correlation between the pore ordering and the fraction of the (100) orientated crystals postulated in the earlier works of Beck et al [95,97] was not confirmed (Figure 11B,C);
- The experimental observations from this study suggested that the density of dislocations is the only parameter of an Al substrate responsible for the pore ordering. While PAA laminas grown on polycrystalline substrates with a relatively large number of dislocations are chaotic, removing those dislocations from the substrate leads to the formation of highly ordered PAA. The dislocation density can be reduced by recrystallization upon achieving the temperature of 483 °C. Such identification of the key role of dislocations density also disagrees with the earlier results reported by Beck and Petrikowski, who found this parameter to be insignificant [95].

The relationship between the density of dislocations in Al and the pore arrangement in PAA established by Norek and Szamyer can be rationalized using the idea proposed by Stępniowski et al. [104]. Electrostatics dictates that the electric field just outside a perfect conductor is perpendicular to the surface. Stępniowski et al. have assumed that the areas with high concentrations of dislocations function as electron scattering centers, which affects the distribution of the electric field lines outside an Al substrate. The authors referred to the electroconvective model of PAA formation where the growth of pores is assisted by the Coulomb forces that are responsible for the motion of electrolyte anions and entrained fluids toward the anode surface [16,17]. The electrical properties of the metallic substrate are changed by the introduction of a significant number of dislocations. Hence, nanochannels formed in PAA can also deviate randomly from their alignment, which interferes with their hexagonal arrangement as well (such coupling of electric fields and fluid mechanics/chemistry is common in microfluidics and may also be manifested when a system is downsized to nanoscale) [105].

### 4.5. A Mismatch between the Grain Boundaries in Al and the Boundaries of Coherent Domains in PAA and Attributing the Hexagonal Ordering to Repulsion between Neighboring Pores

**Figure 12:**
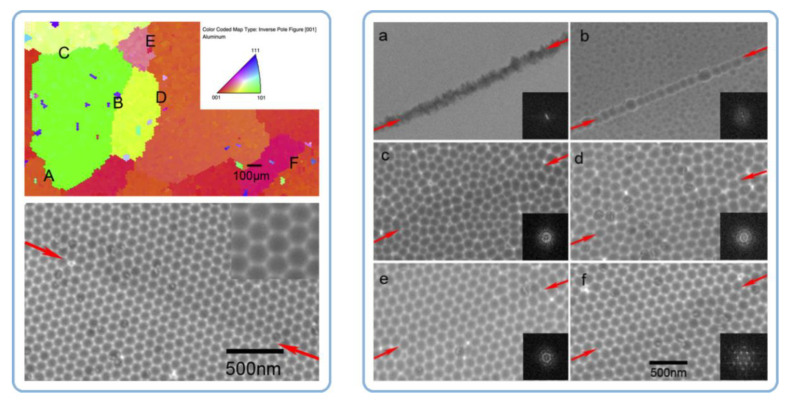
**Top left**: an orientation map of an Al substrate (obtained using the Electron Backscatter Diffraction method, EBSD) on which different crystallographic directions aligned with the axis normal to the sample surface are coded with different colors (see the inset). **Below left**: an SEM micrograph of hexagonally ordered concaves on Al surface visible after the removal of PAA by wet etching (the boundary between two Al grains with different orientation is shown with red arrows). **Right**: the evolution of the Al substrate surface texture above a boundary between two Al grains (marked with red arrows) upon anodizing for 0 min (**a**), 1 min (**b**), 5 min (**c**), 25 min (**d**), 125 min (**e**), and 40 h (**f**). Reproduced with permission from [106]. Copyright 2008 Elsevier.

Fan et al. have investigated the ordered domains in PAA with respect to the grain boundaries in Al substrate using the Electron Backscatter Diffraction (EBSD) method (see the top left image in Figure 12) [106]. The study found a mismatch between the boundaries of Al grains with different crystallographic orientations and the boundaries of coherent domains in PAA. Well-ordered domains of hexagonal pores did not terminate at metal grain boundaries but extended across them instead (see the SEM micrographs in Figure 12). The authors came to the conclusion that the formation of the self-ordered structure depends on the repulsive forces between neighboring pores but not on the orientation of a crystal in the aluminum substrate. It must be noted that the conclusion made by Fan et al. is in full conformity with the known common principles of self-organization in electrochemical systems: different sites of a system can “communicate” with each other, and the appearance of couplings between them gives rise to well-ordered dissipative patterns [6].

In summary, accumulated empirical data in this area still create much controversy. After multiple studies, there is still no conclusive evidence linking morphological and microstructure properties of the Al substrate to the mechanism of self-organization of PAA. The emergence of hexagonal patterns with well-defined geometric parameters on anodized aluminum is most likely primarily caused by other factors. However, it seems plausible that such patterns can undergo a minor shift or rotation relative to the substrate surface to move its individual cells to positions where the etching of pores in metal becomes more energetically favorable (see, for instance, the adjustment mechanism proposed by Napolskii et al [101]).

Another important conclusion is that aspiring perfection in PAA morphology by the deliberate elimination of all possible factors that could lead to defects (e.g., by using perfectly smooth single-crystalline substrates) may be also pointless from an evolutionary standpoint. Once we have dissipative structures, we can speak of self-organization [47]. Dissipative structures arise beyond the bifurcation points, which are characteristic for chaotic systems where instabilities and fluctuations play a crucial role. In other words, just as mutations are the necessary instrument for evolution in biological systems, random (stochastic) events are essential to temporal and spatial evolution in physics and chemistry. Thus, the spontaneous emergence of morphological imperfections in porous structures that restrict the well-ordered domain size to several micrometers is presumably the unavoidable inherent natural property of the PAA system evolving from chaos to order under the appropriate anodizing regime. Therefore, ideally ordered PAA textures are unlikely to be obtained in a spontaneous self-organization process, and thus, they are also unlikely to be achieved solely by optimizing anodizing conditions without using artificial pre-patterning methods.

## 5. Structure and Composition of PAA Laminas

The very essence of self-organization is that molecules and other mobile particles demonstrate their ability to participate in cooperative (coherent) motions, which are responsible for the formation of dissipative structures. This ability of small particles to move coherently weakens upon approaching an equilibrium state in which dissipative structures disintegrate. The existence of such spatiotemporal structures over an extended period of time requires maintaining a so-called “unstable steady-state” in an open system which is far from equilibrium, e.g., in a chemical reactor (the subject of chaos control and stabilization of steady-states in electrochemical systems is treated in detail in the monograph by M. Orlik [6]).

When looking specifically at the growth of PAA, it is logical to raise a question about the possible nature of such particles that participate in cooperative motions and sustain the formation of a stable porous structure. Basically, these particles should meet two key requirements: they must exist in a highly labile condition in the vicinity of the anode where PAA cells are shaped, and they must also undergo rapid aggregation into solid alumina once honeycombs are finally formed (i.e., upon reaching a certain height above the metal surface during the “vertical” translation of a “horizontal” hexagonal pattern, so that a stable three-dimensional solid porous structure can be continuously formed). In other words, the cooperative movement of loose (that is, stabilized against aggregation) (sub-)nanometer “building blocks” is necessary for the continuing creation of the interpore walls with well-defined self-organizing periodicity and other geometric parameters. Once pore walls are shaped, the rapid aggregation of these “building blocks” should provide mechanical stability and integrity of PAA without an external support of a steady-state (i.e., the formation of solid aggregates prevents the disintegration of PAA ex situ without stabilizing these dissipative structures by the corresponding driving forces).

Also worth mentioning is the distinction between self-organization of particles and another widely spread phenomenon leading to the spontaneous formation of ordered arrays of monomer units, which is termed self-assembly (the confusion regarding these two phenomena can be occasionally encountered in the literature [107], and such wording as “self-assembly of pores” can be found even in a paper co-authored by renowned scholars in the field of anodizing [108]). Self-assembly occurs owing to the presence of anchoring sites of amphiphilic molecules that determine their arrangement within an aggregate (this can be exemplified by the formation of micelles from surfactants that contain hydrophilic head groups and hydrophobic tails). However, self-organization is based on a different principle, and aggregated particles can be highly symmetrical without any distinctive anchoring sites.

All of the above considerations point to the conclusion that pore walls are most likely composed of colloidal alumina particles which were initially stabilized against aggregation (generally, by surface charging [109]) and then aggregated into a typical gel-like hydrated alumina structure (pristine PAA laminas, as obtained from the electrolytic bath, usually consist of amorphous boehmite *γ*-AlOOH with a large amount of absorbed water and anionic impurity). Such particles can be set in coherent motion via different possible transport mechanisms (e.g., migration or convection) and participate in the self-organization process.

### 5.1. Theoretical Models That Provide Insight into the Physical Properties and Composition of PAA by Considering Its Colloidal Morphology

**Figure 13:**
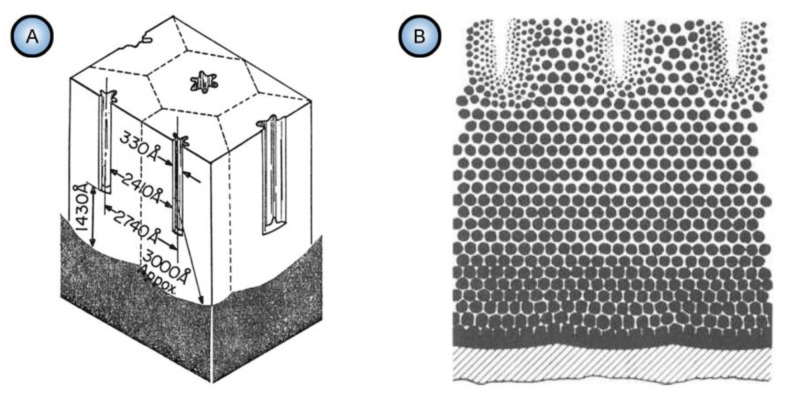
A comparison of two PAA structures assumed by the geometric model proposed by Keller et al. (**A**) and by the colloidal gel model proposed by Murphy and Michelson (**B**). In the colloidal gel model, electrolyte oxyanions adsorb to the surface of the particles and then facilitate the formation of a complex hydrogen-bonded water-containing system through which ionic conduction occurs. Reproduced from [111] with permission from The Electrochemical Society (1968).

In the late 1960s, Michelson has studied some dynamic current–voltage characteristics exhibited by PAA in an electrolytic cell and found his experimental observations to be inconsistent with the conventional view of that time: that PAA was formed due to the pore base dissolution mechanism (the model proposed by Keller et al.; see Figure 13A) [110,111].

Nevertheless, the obtained results could be successfully explained by using the colloidal gel model of PAA structure earlier proposed by Murphy and Michelson (Figure 13B) [112]. The basic postulates of this PAA growth model are as follows:

- The innermost “barrier layer” represents almost anhydrous electrolyte-contaminated alumina. Al^3+^ ions are ejected from the metallic substrate under the influence of the electromagnetic field and react with oxygen-containing species within this barrier layer to form new oxide;
- The electrolyte anions and OH^−^ move toward the oxide layer, and then, they penetrate it at selected points and break it up to form an agglomeration of discrete colloidal particles;
- The outermost layer of the anodic films represents agglomerated nanocrystalline anhydrous oxide particles with gaps in between filled by a complex hydrogen-bonded system of water, electrolyte anions, OH^−^, and H^+^. The mechanisms of the oxide formation and of the appearance of an array of cylindrical pores in PAA were viewed by the authors as separate. The importance of these microscopically observable cylindrical pores in the mechanism of oxide layer formation was held to be less than in competing hypotheses;
- Oxyanions of electrolyte adsorb to the surface of colloidal alumina particles and stabilize them after separation from the barrier layer. After that, electrolyte anions unite with H_2_O, OH^−^, and H^+^ into one complex hydrogen-bonded system through which the ionic conduction within PAA occurs;
- The formation of PAA takes place in the vicinity of the barrier layer/porous layer interface (i.e., at a certain distance from the metal substrate surface) approached by cations and anions from the opposite directions under the influence of the electromagnetic field.

The colloidal gel model proposed by Murphy and Michelson [111,112] considered such important factors as the type of electrolyte and the chemical nature of anodic oxide in the PAA growth process. It emphasized that electrolyte anions have a constructive (the formation of hydrated and hydrogen-bonded conducting surface) rather than destructive (promoting the complete dissolution of the oxide at the pore base) role in PAA formation.

**Figure 14:**
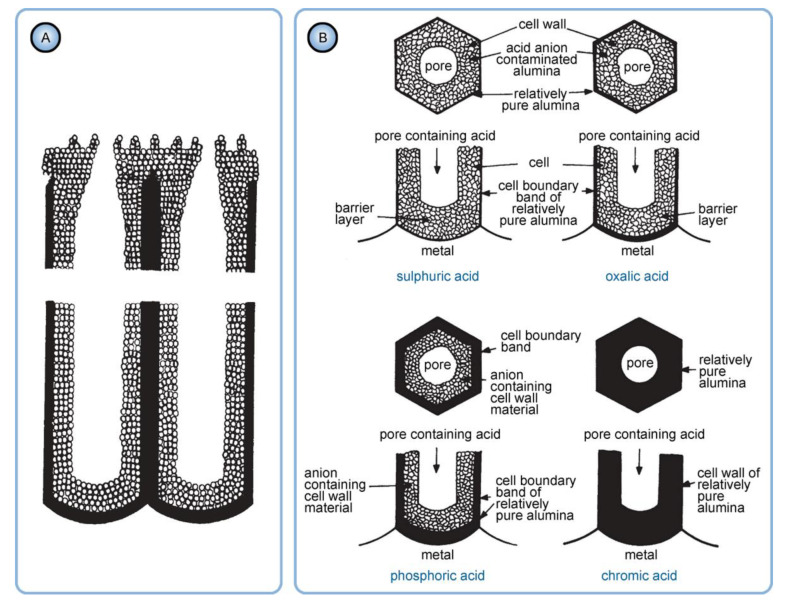
(**A**) A schematic depiction of PAA cells composed of relatively pure alumina and nanocrystalline material containing incorporated electrolyte anions (the morphology suggested by Thompson et al.). (**B**) The use of the model proposed by Thompson et al. for the explanation of different anionic contamination levels in PAA formed in the major used electrolytes (by considering different thicknesses of the polycrystalline anion-contaminated regions adjacent to the pore interiors). The assumed size of crystallites is <2.5 nm. Reproduced with permissions from [113,114]. Copyright 1978 and 1981 Nature.

A decade later, Thompson et al. proposed an updated model for initial PAA growth which was based on two simultaneous processes: the formation of new alumina at the metal/oxide interface due to the migration of mobile ions (A^3+^, OH^−^, O^2−^) through the existing coating layer, and the growth of a differently textured material at the film/solution interface due to the deprotonation of [Al(OH_2_)_6_]^3+^ ions followed by the aggregation and thence precipitation of colloidal hydrated alumina particles [113]. The rate of such precipitation was presumably controlled by electrolyte anions, which could stabilize the suspension of fine colloidal particles and thus delay the precipitation of impure, hydrous alumina. It was assumed that a suspension of negatively charged colloidal particles stabilized by adsorbed electrolyte anions is deposited on top of the electrolyte-free inner layer under the applied field to form an outer microcrystalline contaminated layer (see Figure 14A). While the innermost region of the growing PAA was represented in this model as compact alumina, the outermost region was represented as a gel-like material that loses colloidal particles to the aggressive electrolyte solution.

Thompson and Wood have also adhered to the model presented in Ref [113] in their later work dedicated to the characterization of anionic impurity content and distribution within the walls of PAA cells formed in different electrolytes (Figure 14B) [114]. In that work, the electrolyte-free cell-boundary bands were characterized as having a glassy microcrystalline structure, whereas the outer anion-contaminated region was described as consisting of a gel-like material deposited from initially colloidal alumina.

The formation of stabilized colloidal alumina suspension that later coagulates into solid interpore walls is also a cornerstone of the recently proposed PAA formation model based on analogy with another self-organization phenomenon known from fluid dynamics—namely, the formation of hexagonal Reyleigh–Bénard cells [16]. In this model, electroconvection is responsible for the coherent motion of colloidal particles, which serve as “building blocks” for PAA cell construction.

### 5.2. The Mechanism of Polycrystalline PAA Formation from Electrochemically Generated Colloidal Dispersion Assisted by DLVO Interaction Forces

**Figure 15:**
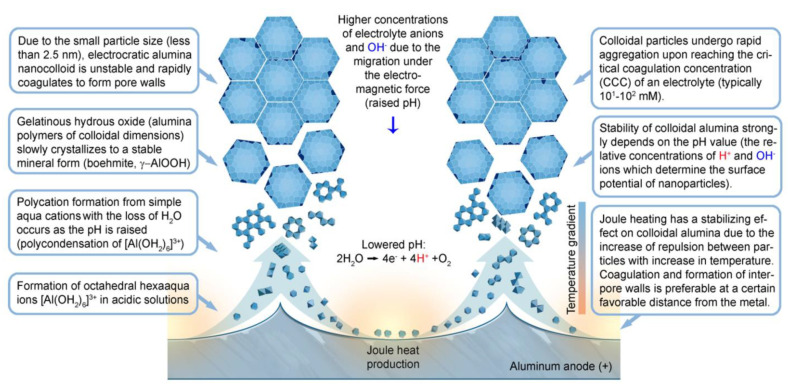
Schematic representation of the mechanism of polycrystalline PAA growth and explanation of the factors associated with the stability of colloidal alumina dispersion. The formation of simple [Al(H_2_O)_6_]^3+^ aqua cations is followed by their polycondensation into polyoxo compounds (with elimination of H_2_O molecules) and then the nucleation of hydrated polymeric alumina particles. These colloidal particles are capable of coagulation and forming the interpore walls. The stability of particles toward aggregation is determined by a synergistic effect of multiple opposing factors: the increased temperature (due to Joule heating) and the decreased pH value in the proximity of the anode surface are likely to stabilize the dispersion, whereas the small particle size and the increased concentration of the indifferent electrolyte anions (due to their Coulomb attraction to the anode) should cause rapid coagulation.

Today, the colloidal morphology of PAA layers with an average particle size not larger than a few nanometers is an acknowledged experimental fact in spite of the different terminology sometimes used by different researchers (micro- or polycrystalline [113,114], amorphous [56], X-ray amorphous [97], etc.). However, the issue of the rapid aggregation mechanism of initially stabilized colloidal alumina at locations where cell walls’ growth takes place remains open. More precisely, we should speak of a rapid coagulation process—in contrast to flocculation (another type of aggregation) of colloids, which results in loose flocks that can be disrupted by stirring [109]. An aqueous dispersion of alumina particles can be most likely treated as an “electrocratic” colloid whose stability to aggregation depends on the interplay between van der Waals attraction and electrostatic repulsion. The stability behavior of such colloids is described by DLVO theory (named after Derjaguin and Landau, and Verwey and Overbeek, who created this approach in the Soviet Union in the 1930s and in the Netherlands in the 1940s, respectively). DLVO theory is usually considered to be the starting point for the description of both the purely electrocratic sols (what many colloids, in fact, are) and more complex systems [109]. Some considerations derived from DLVO theory that can contribute to a better understanding of the aggregation behavior of fine alumina dispersion near the anode surface are as follows (see also Figure 15):

- The reason for the rapid coagulation of hydrated alumina particles shortly after their nucleation can be their small size. In order to aggregate, colloidal particles have to overcome a potential energy barrier (a local maximum of the DLVO curve, which shows the interaction potential of two closely located spherical particles as a function of the distance between them). This barrier can be overcome due to the energy of Brownian motion whose average intensity approximates *kT*. This energy of a particle moving by Brownian motion should be independent of particle size, whereas the barrier to coagulation should decrease with the decrease in particle radius (this follows from the analysis of the mathematical expression for the interaction potential). This is the reason why electrocratic nano-colloids are generally difficult to stabilize [109] and also why alumina nanoparticles must be prone to rapid coagulation after performing their role in the self-organization process (probably, alumina is delivered from pore centers to the interpore walls with a moving viscous fluid not as already formed colloidal particles but in form of their precursors, i.e., intermediate polynuclear complexes resulting from the polycondensation of [Al(OH_2_)_6_]^3+^ ions in large concentrations; therefore, self-organization has sufficient time before the final coagulation occurs);
- Increased temperature should produce increases in the repulsion between particles, which could contribute to colloid stabilization near the anode where Joule heat is produced. Since temperature is included into the corresponding mathematical expression as absolute temperature in K, this dependency under anodizing conditions is possibly not very pronounced [109]. However, numerous experiments show that the increase in anodizing temperature leads to a noticeable increase in PAA pore size, whereas the cell size remains practically unchanged and seems to be primarily voltage-dependent [53,115,116]. This effect can be attributed either to the enhanced chemical dissolution of alumina at a higher temperature, or to a higher loss of stabilized colloidal alumina particles, which leads to formation of thinner interpore walls and therefore to the increased porosity of PAA. Of course, the combination of both of those factors is also possible. In extreme cases, when a high temperature of the electrolyte solution is combined with the additional intense heat production at the anode, the etching of a hexagonal pattern on anodized aluminum surface without the deposition of any analytically detectable amount of alumina on top of it can be also observed [54];
- The stability of an electrocratic dispersion very strongly depends on the indifferent electrolyte concentration and valence. The potential barrier decreases sharply with any increase in electrolyte concentration due to compression and the following collapse of the double layer [109]. The barrier goes to zero, and the colloid undergoes rapid aggregation upon reaching the so-called critical coagulation concentration (CCC) typically ranging from a few tens to a few hundreds of mM. These concentrations are very close to those used in the anodic oxidation of Al (0.3 M for the most classical electrolyte compositions), which should facilitate the aggregation of nanoparticles into polycrystalline pore walls. The dependence of the CCC on the valence of an electrolyte can be more or less pronounced depending on the specific value of the Stern potential for given particles, but for both the high and the low values of the Stern potential, the CCC either steeply or moderately decreases at higher electrolyte valences. This can be the possible explanation for why PAA synthesis is usually more successful when using moderately concentrated polyprotic acids (H_2_C_2_O_4_, H_2_SO_4_, H_3_PO_4_ etc.), and only negligible amounts of alumina can be deposited onto the waved surface of metallic substrates when using monoprotic electrolytes of comparable concentrations (HCOOH, [16] HIO_3_, HIO_4_ [88]);
- The effect of the Stern potential on the stability of colloids is also very significant: very low potentials cannot produce a sufficient barrier to coagulation and consequently also cannot assure reasonable stability. The magnitude of the Stern potential can be controlled by varying the surface potential of a nanoparticle, which depends on the relative concentration of the potential determining ions (for oxides, the pH) [109]. Different pH regions (or strata) can also exist in close proximity to the anodized aluminum surface due to oxidation of water and generation of H^+^, as well as due to the attraction of negatively charged OH^−^ ions toward the positively charged anode (these regions corresponding to low and high pH values should come into balance at a certain height above the substrate surface; see the detailed graphical explanation in Figure 23 reproduced from Ref [16] where the coagulation of colloidal alumina was rationalized on the basis of a similar principle).

### 5.3. The Incorporation of Electrolyte Impurity into PAA through a Process of Anion Exchange

Another problem that puzzled researchers for many years is a relatively large amount of anionic impurity in PAA (variable admixture content from 4.92 to 11.1 wt % was reported for different MA conditions) [55,56,117]. In order to better understand the possible mechanisms of the incorporation of acid residues into PAA, it is helpful to look at the common stages of nonstoichiometric amphoteric amorphous alumina formation in acidic environment. At the beginning, [Al(OH_2_)_6_]^3+^ ions (which are also generated and transferred into the electrolyte solution during aluminum anodizing process: atomic absorption spectroscopy measurements have revealed that only 77% of all produced [Al(OH_2_)_6_]^3+^ ions are consumed for PAA formation, while 23% still remain dissolved in the aqueous phase after the completion of the electrosynthesis [92]) condense into complex polycations with fractional nominal charges on aluminum atoms (e.g., [AlO_4_(Al(OH_2_))_12_]^7+^). After that, the resulting hydrated polymeric oxide forms colloidal particles that slowly consolidate into a stable mineral phase [118]. The positive charge of such polymeric frameworks consisting of polynuclear cations is balanced by adsorbed labile counterions. These counterions can be replaced by other ions from the solution that bear charges of the same sign (for example, OH^−^ can be replaced by sulfate, phosphate, or oxalate anions). This phenomenon is widely known as ion exchange. Aluminum hydroxide gels belong to the most practically important inorganic synthetic anion exchangers; however, these are destroyed by the acid or alkali treatment (actually, hydrated alumina exhibits amphoteric properties and acts as an anion-exchanger only at low pH values) [119]. The grain boundary diffusion of counterions is usually considered as the slowest (rate-limiting) stage of ion exchange. The velocity of this process can be increased by decreasing the grain size or by increasing the temperature. Taking into account such factors as the nanocrystalline composition of gel-like pore interiors in PAA (the presumable grain size is ≤2.5 nm [113]) and heat release from the anode surface during anodizing (in some early hypotheses, the anode temperature was estimated as high as 125 °C [120]), the conditions for the incorporation of electrolyte species into PAA via such mechanisms can be considered as favorable. Interestingly, the anion exchange capacity of hydrated alumina increases as pH decreases, and the value obtained for commercial chromatographic alumina exceeds 2.0 mequiv g^−1^ upon reaching pH = 4.0 [121]. This corresponds either to 9.6 × 10^−2^ g of SO_4_^2−^ ions or to 8.8 × 10^−2^ g of C_2_O_4_^2−^ ions per 1 g of alumina, which correlates well with the typically reported percentages of anionic admixture in PAA (up to approximately 10 wt %) [55,56]. Given the high contact area of nanocrystalline PAA and aqueous electrolyte, the limit value for the exchange capacity (which can be possibly achieved under certain optimal conditions for ion diffusion and adsorption) can be even significantly higher than known for the micrometer-sized crystals of chromatographic alumina.

**Figure 16:**
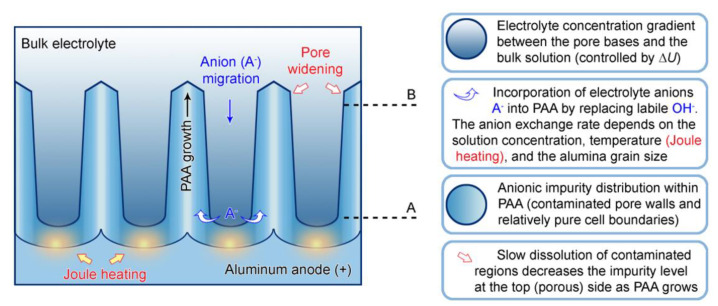
Schematic representation of non-uniform electrolyte impurity distribution within PAA controlled by two key processes: ion exchange (i.e., replacement of labile OH^−^ counterions by electrolyte anions from the solution) and slow chemical dissolution of the anion-contaminated regions of pore walls. Since the rate of ion exchange and diffusion within PAA depends on the electrolyte concentration, temperature, and the grain size, the main fraction of electrolyte anions is incorporated into pore walls at the pore bases (position A). As the pore walls grow higher, a slow densification of PAA to a stable mineral form occurs, and the anion exchange rate decreases (position B). The slow dissolution of contaminated pore interiors results in a gradually increased ratio of pure alumina to contaminated alumina. Therefore, there is a contamination level gradient along the pore growth direction.

In the light of the arguments outlined above, the following factors should make the major impact on the resulting anionic impurity content in PAA:

- A period of time, during which a particular segment of a growing cylindrical nanochannel stays in contact with the electrolyte solution, and the corresponding exchange of ions between the liquid and the solid phase takes place;
- The concentration of electrolyte in the aqueous phase within PAA nanochannels (because the difference in concentrations is the driving force of counterion diffusion toward the sites within the solid phase where ion exchange occurs). It should be noted that the overall concentration of an electrolyte in the bath and the local concentration within PAA nanochannels are not the same. Due to the migration of anions to the anode under the electromagnetic force, a concentration gradient is established (electrolyte species are accumulated near the anode surface but are not consumed in RedOx reactions). Some early investigations pointed to the electrolyte concentration at the pore base three times higher than the bulk electrolyte concentration during anodizing [120]. Thus, higher anionic impurity content can be expected in PAA if a larger potential difference is applied between the electrodes. This has been experimentally confirmed by the compositional analysis of PAA laminas formed under HA conditions, which demonstrated an increase in the impurity level by 88% in comparison with PAA layers prepared under MA conditions [122]);
- Since the rate of grain boundary diffusion depends on temperature, higher impurity contents can be expected if anodizing is performed either in solutions at high temperatures or under high voltages and current densities when Joule heating of the anode becomes important (this additionally explains why PAA samples synthesized under HA conditions have higher contamination levels);
- As mentioned above, hydrated alumina is noticeably soluble in acidic environment (at pH < 4). It is well-known that PAA cells consist of two layers: the relatively pure cell boundaries and the electrolyte-contaminated pore walls (i.e., polycrystalline regions directly around the pores where adsorbed counterions are most prominently contained) [56]. As the tubular nanochannels grow and propagate further in the direction of the metallic substrate, the earlier formed sections of contaminated walls can be again moderately dissolved in the aggressive electrolyte medium (because of such chemical dissolution, the pore openings in PAA acquire the well-known “trumpet shape” [13]). Slow etching of the electrolyte-contaminated region results in the increase in the fraction of relatively pure alumina that remains protected from a direct contact with the etchant. Thus, chemical dissolution is the only involved competing process which increases the purity of a PAA nanochannel segment upon a longer exposure to the aqueous electrolyte solution (see Figure 16).

**Figure 17:**
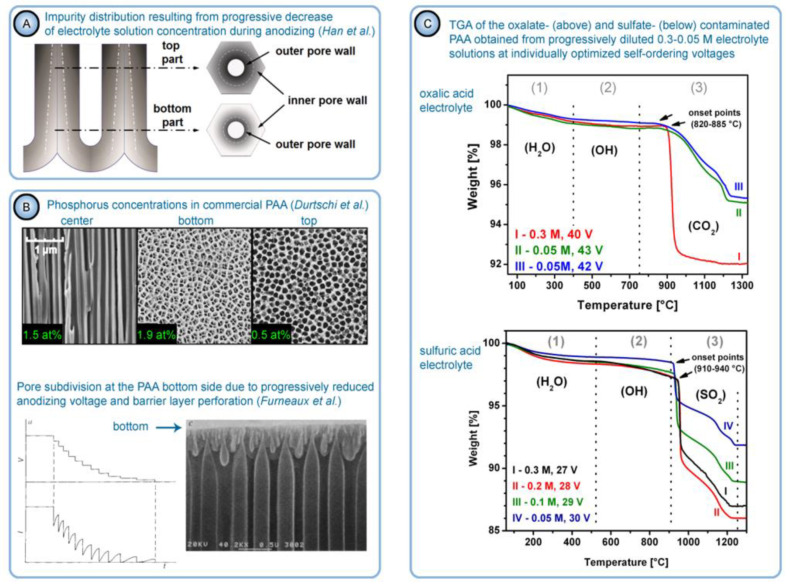
(**A**) The character of non-uniform anionic impurity distribution (shown as grayscale) in PAA attributed to the progressive decrease in electrolyte solution concentration during anodizing (Han et al.). (**B**) Another type of contamination distribution found in commercial phosphate-containing PAA membranes with the highest concentration at the pore bottom (the top image in (**B**)), and the explanation of different topography of the top and the bottom surfaces of commercial PAA membranes observed in a scanning electron microscope (the bottom image in (**B**)). (**C**) The results of thermogravimetric analysis (TGA) confirming the successful low-temperature in situ reduction of oxalate and sulfate contamination (approximately by a half) in PAA laminas synthesized using extremely diluted electrolyte solutions (down to 0.05 M) and mild anodizing voltages. Adapted with permissions from [57,123,124,125]. Copyright 2013 and 2020 American Chemical Society, 2005 Elsevier, and 1989 Nature.

Of course, the combined effect of all these factors and the resulting overall contamination level, as well as the gradient in the impurity concentration along the nanochannel growth direction, depends on multiple anodizing parameters (the type and concentration of an electrolyte, pH, applied voltage, temperature, anodizing process duration, etc.) and thus is not readily predictable a priori. For instance, Han et al. have investigated the anodizing process using H_2_C_2_O_4_ solution in a cell where the electrolyte concentration was decreased during anodizing over an extended period of time [123]. This decrease in the dissolved acid concentration also resulted in the decrease in the thickness of the anion-contaminated regions with respect to the thickness of the pure regions along lines that pass between adjacent PAA cells. The detected contamination level at the top (porous) side was higher than at the bottom (sealed with the barrier layer) side (Figure 17A). Durtschi et al. examined commercial PAA membranes synthesized in H_3_PO_4_ and also reported a non-uniform distribution of the impurity-related elements in the thickness of PAA [124]. The concentrations of phosphorus at the opposite surfaces of PAA and in the middle of the film cross-section varied by a factor of 3−4 (see the top image in Figure 17B). In contrast to the results reported by Han et al., Durtschi et al. have found the lowest contamination level on the top side and the highest at the bottom (0.5 and 1.9 wt %, respectively). The sides of commercially prepared PAA membranes (with a removed barrier layer) can be correctly identified from the size and the shape of pore openings. The difference in their appearance underneath an electron microscope results from a progressive voltage decrease at the end of the anodizing process to reduce the barrier layer thickness (see the bottom image in Figure 17B) [125]. After such preparation, a thin barrier layer can be completely removed by wet etching, while the relatively thick interpore walls remain undamaged. The impurity percentages obtained by Durtschi et al. are comparatively low, which can be also explained by the utilization of the wet etching method to open commercial membranes through: while the contaminated region close to the pore interior was partially removed by an etchant, the relatively pure alumina located at the cell boundaries stayed intact. Another very recent experimental investigation found that the key to controlling and reducing undesirable anionic contamination in PAA lies in selection of self-ordering regimes that combine the lowest possible electrolyte concentrations and applied anodizing voltages [57]. In two series of experiments conducted using different electrolytes (H_2_C_2_O_4_ and H_2_SO_4_), the anionic admixture content in hexagonally arranged PAA layers was reduced by 40–50% by diluting the electrolyte solutions from conventional 0.3 M down to 0.05 M concentration while keeping the applied voltages within the ranges corresponding to mild anodizing (MA) conditions (Figure 17C). This work has demonstrated a complex synergistic effect of electrolyte concentrations and applied voltages on the resulting admixture content. It provided the basis for a new low-temperature purification method that involves no annealing procedure and thus can be very helpful for the preservation of an amorphous PAA structure, which is degradable in acidic or alkaline solutions, as well as for the elimination of an anionic admixture that exhibits exceptional thermal stability (e.g., phosphates that remain incorporated into alumina matrix even after annealing at 1400 °C [55]).

In conclusion, one can say that the conventionally used definition “anodic aluminum oxide” is arbitrary because PAA consists of mixed nonstoichiometric oxide-hydroxide (boehmite, *γ*-AlOOH) discrete colloidal particles with a considerable amount of absorbed water and anionic impurity. All these components have their constructive roles in the PAA formation mechanism and are inherent parts of the resulting self-organized structure.

## 6. Theoretical Models Explaining PAA Formation

### 6.1. The PAA Growth Models Based on Ionic Migration and Diffusion through a Solid “Barrier Layer” and Electric-Field-Assisted Oxide Dissolution

**Figure 18:**
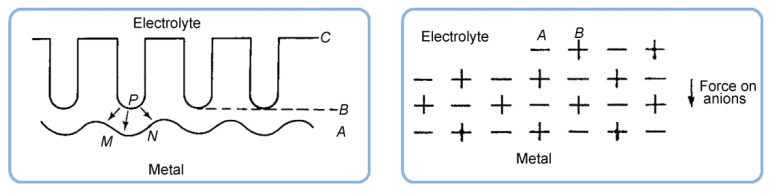
The mechanism for PAA formation in an acid bath proposed by Hoar and Mott. Left: the arrows *PM* and *PN* demonstrate the suggested paths of oxygen-containing species through PAA, Al^3+^ ions move from *MN* toward *P*, where they can neutralize O^2−^ and be transferred into the solution, OH^−^ move from *P* toward *MN* where they neutralize Al^3+^ formed from the metal. Right: a schematic representation of PAA as a crystalline material in contact with the electrolyte solution. Reproduced with permission from [126]. Copyright 1959 Elsevier (Pergamon Press).

The first attempts to provide theoretical insight into the formation of PAA trace back to the mid-1950s when these amazing structures were first observed underneath an electron microscope by Keller et al., and the new phenomenon urgently needed an explanation [110]. A lot of top minds of that time worked to address this issue, including Sir Nevill Mott (the 1977 Nobel Prize winner in physics). Hoar and Mott proposed their mechanism for PAA formation in an acid bath in 1959 [126]. According to this model, PAA growth starts with the initial formation of a compact oxide film on the metal surface, which is analogous to that formed in non-acid solutions. After that, anodic oxide continues to grow as a much thicker porous layer on top of this compact layer of a constant thickness. At that time, there were three well-established metal oxidation theories that explained the formation of compact films. According to Cabrera–Mott theory, the oxide growth was governed by the high-field conduction mechanism in which the cation transfer across the metal/oxide interface was considered to be the rate-determining step [127]. Verwey (whose theory preceded Cabrera and Mott) assumed that the growth rate was limited by the propagation of Al^3+^ ions through the oxide bulk (or the pre-formed FCC lattice of oxygen ions) due to the high field [128]. Dewald theory (the extension to Cabrera–Mott theory which included space charge effects) treated the transfer across the oxide/electrolyte interface as the rate-determining step [129,130]. However, Hoar and Mott noted that in all those different theories, Al^3+^ ions were supposed to pass through the entire oxide film to reach the oxide/electrolyte interface and neutralize oxide anions there [126]. In the case with compact films, their growth was supposed to stop when the field in the oxide film (which causes the ion transport mediated by interstices) drops to a certain critically small value. Increased oxide film thickness means a longer distance that ions need to overcome, hence the limitation of the growth rate and the typical logarithmic growth law. In an attempt to explain the PAA growth mechanism in which the separation between the pore base and the metal (earlier defined by Keller et al. as the “barrier layer” [110]) remains unchanged during the entire anodizing process, Hoar and Mott have added the following details to the previous theory of the compact film:

- In addition to the movement of Al^3+^ ions toward the oxide/electrolyte interface at the pore base, some oxygen must also pass through the film in the opposite direction and neutralize Al^3+^ ions near the metal/oxide interface, increasing the separation between pore bottoms and etched concavities on the metal (Figure 18, left);
- The acid must dissolve the oxide at the pore base, keeping the compact (“barrier”) layer thickness constant;
- Both of the foregoing conditions are satisfied if the following reaction between protons from the electrolyte and O^2−^ ions from the oxide lattice (Figure 18, right) takes place at the pore bottom: O^2−^ + H^+^ ↔ OH^−^. First, hydroxyls are more mobile than O^2−^ under the influence of the field and thus can move more easily toward the metal and deliver oxygen more efficiently. Second, Al^3+^ cations can be ejected from the oxide surface into the electrolyte solution upon such removal of anions from the lattice, which agrees with the experimental data;
- For a certain compact layer thickness, the rate of Al^3+^ transfer (which reacts with O^2−^ at the oxide/electrolyte interface to form new oxide) will be equal to the rate of OH^−^ transfer (whose loss is compensated by the ejection of Al^3+^ from the lattice into the electrolyte solution). That is, the increase in film thickness will come into balance with the decrease;
- The model by Hoar and Mott also implied a self-adjustment mechanism for the preservation of the porous structure: if the curvature of a pore bottom becomes too great (i.e., a flattening process starts), the field, the oxide dissolution rate, and the ion transfer rates will increase at this location and promote pore deepening.

Thus, Hoar–Mott theory rested on the concept of equilibrium established between “barrier-type” oxide formation and its field-assisted dissolution. In the light of the empirical data available at the time of creation, such a model certainly seemed viable. It was very well received by the anodizing community and implemented into the mainstream PAA research. It can fairly be said that despite some differences in opinions (e.g., regarding the role of temperature and electrolyte concentration gradient between the pore bases and the remainder of the electrolyte [110,126,131]), Hoar–Mott theory was virtually considered by many researchers of the day to be the framework into which all their further developments must fit. In the early 1960s, Murphy and Michelson proposed a colloidal gel model of PAA formation that substantially differed from the conventionally accepted pore base dissolution mechanism (this theory is treated in detail in Section 5.1 of this review; see Figure 13) [112]. Later, this model was also experimentally confirmed by Michelson via current–voltage characteristics measurements, as well as by some other researchers on the basis of spectroscopic and microscopic investigations (Csocán noted that Murphy–Michelson theory provided a better explanation for the microscopically observed laminar PAA morphology than the conventional theory, which presumably needed a fundamental re-examination; Dorsey suggested that porous oxide may arise due to the transformation of a transition region above the “barrier layer” which has an open structure that is permeable to the electrolyte) [111,132,133]. Shortly after the publication by Michelson, the colloidal gel model was criticized by O’Sullivan and Wood, who expressed the view that it was incompletely supported by direct empirical observation [13]. In their work (communicated by N. F. Mott), the authors concluded that there was no convincing evidence provided for the alternative theory by Murphy and Michelson, and they presented their special experiments based on electrolyte concentration and temperature variation in support of the model by Hoar and Mott.

Even now, six decades later, modified Cabrera–Mott theory is still frequently employed to analyze relationships between parameters describing the growth of nanoporous oxide films (e.g., PAA or anodic titania nanotubes) [15]. In contemporary literature, the formation of ordered pores is often considered to be closely related to the electric field strength impressed in the barrier oxide layer, which determines the ionic current density *j* [134]. The transport of charged ions through the barrier layer (that is, *j*) is supposed to be a function of temperature-dependent material-related constants and the effective electric field *E* = ∆*U*/*t*_b_, where *t*_b_ is the barrier layer thickness (the parameter that remains constant throughout the entire anodizing process).

**Figure 19:**
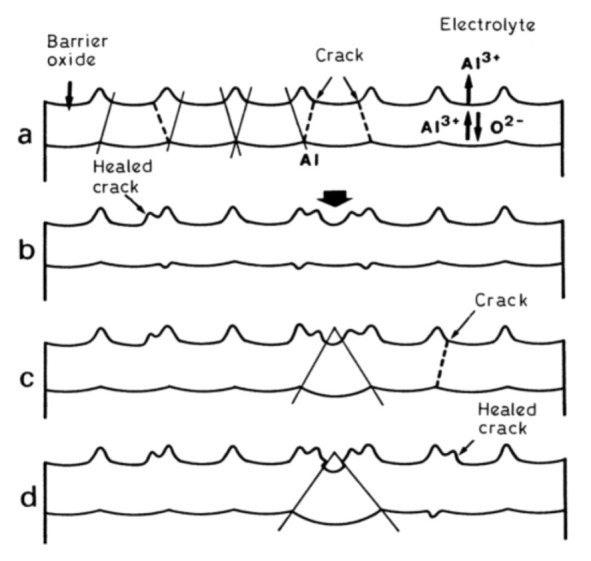
A schematic diagram showing the stages of pore development during anodizing (as proposed in the model by Thompson et al.). Reproduced with permission from [136]. Copyright 1997 Elsevier.

Thompson et al. developed a rather comprehensive PAA formation theory between 1978 and 1997 [113,135,136]. On the basis of the experiments conducted in H_3_PO_4_ solutions, a conclusion was made that the barrier layer thickness must be a function of the applied voltage. The observed flattening of the natural defects on initially rough Al substrates was attributed to more rapid formation of the barrier-type oxide at the ridges on metal. The model proposed by Thompson et al. included two simultaneous processes (Figure 19a):

- Diffusion of Al^3+^ from metal and O^2−^/OH^−^ from the side of electrolyte solution toward the metal/oxide interface where new oxide is formed;
- Ejection of hydrated Al^3+^ ions to the electrolyte solution.

These two parallel processes result in two alumina layers: the electrolyte-free inner layer is covered by an outer layer of fine anion-contaminated microcrystallites, which is formed via the deprotonation of hydrated Al^3+^ ions and their aggregation into a stabilized colloidal suspension followed by precipitation under the electric field (the role of electrolyte anions in this proposed mechanism is discussed in detail in Section 5.1 of this review; see Figure 14). The following stages of the pore nucleation mechanism were postulated [135]:

- A scalloped native barrier-type oxide grows over the also slightly scalloped Al surface left by the electropolishing procedure;
- In the beginning of the anodizing, pores initiate at cracks and surface imperfections, leaving an electric field concentrated below the regions where the oxide film is thinner, hence the local dissolution of oxide (Figure 19b);
- The pore bottom deepening leads to the formation of a major pore at the expense of the former shallow pores (Figure 19c,d).

It was supposed that the average field across the barrier layer controls the film growth rate at the metal/oxide interface, while the local field at the pore bottom determines the dissolution rate at the oxide/electrolyte interface. The film growth rate is constant, but the dissolution rate increases as the pore curvature radius decreases (compare with Hoar–Mott theory, where the oxide dissolution rate was assumed to locally increase upon the enlargement of the curvature of a pore bottom [126]). Thus, the film dissolution rate enlarges the undersized pores and slows down if the pore radius becomes oversized. These two competing processes were supposed to keep the pore radius constant. In Thompson’s theory, the essentially important condition for the porous film formation is that the Al^3+^ ions remain outwardly mobile at high current efficiencies and can be ejected directly into the electrolyte solution from the oxide/electrolyte interface [136]. Otherwise, such Al^3+^ ions would “heal” the embryo pores at the initial stage of their development.

**Figure 20:**
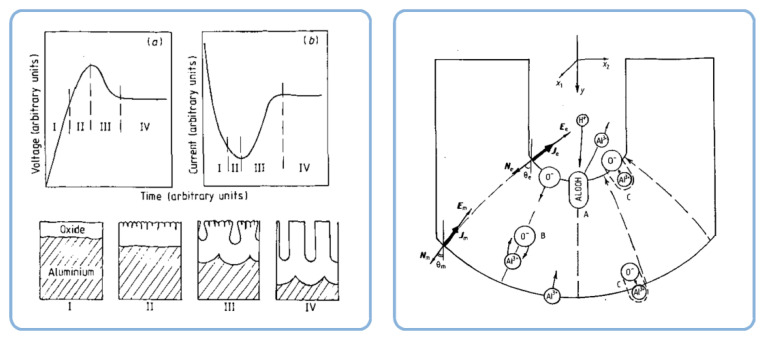
Left: a Δ*U*–*t* (*a*) and an *I*–*t* (*b*) curve shape considered in Parkhutik–Shershulsky theory as typical for a galvanostatic and a potentiostatic anodizing process, respectively (above), and the corresponding stages of pore formation suggested in the model (below). Right: the elementary processes involved into the pore formation—namely, the field-assisted dissolution with the participation of positively charged H^+^ ions (A), the movement of Al^3+^ and O^−^ through the “barrier layer” (B), and the oxide growth at both film interfaces (C). Reproduced with permission from [137]. Copyright 1992 IOP Publishing.

Some underlying concepts of the theory proposed by the Manchester group (Thompson et al.) were also inherited by later developed models. One of such principal points was the inhomogeneous electromagnetic field distribution on the oxide surface: a pore bottom was considered to be the place where the thickness and the electric resistance of alumina are smaller, and therefore, there must be a peak electromagnetic potential. This peak potential leads to the dramatic ionic current increase and intense local dissolution of alumina; hence, pores propagate deeper into the oxide layer at the preferential positions (Figure 19b–d). Parkhutik and Shershulsky have elaborated a theoretical model (Figure 20) to derive a system of equations describing a steady-state single-pore growth [137]. The final analytical expressions that related the resulting radii (or curvatures) of pores and cells to anodizing parameters (such as ∆*U* or pH) were obtained for the modeled system in which the pore bottom and the cell bottom were represented as two concentric hemispheres of radii *R*_e_ and *R*_m_ (the subscripts stand for the interfaces adjacent to electrolyte and metal, respectively). Thus, the assumed barrier oxide thickness *L* = *R*_m_ − *R*_e_ is uniform across the entire area (and constant in time due to equal movement rates of the external and internal oxide boundaries in the steady-state). Therefore, the electric field inhomogeneity in this model is mainly related to the non-planarity of the barrier layer rather than thickness variations. Parkhutik and Shershulsky have concluded that the cell size should linearly depend on the applied voltage, and the dependency of the pore size on the pH value should be quasi-linear. These theoretical conclusions were supported by the experimental data obtained a decade earlier by Ebihara et al. [138]. The main criticism of Parkhutik–Shershulsky theory has been that this model explains neither how the hemispherical shape of a pore bottom starts nor why the pores become hexagonally arranged [91]. Although the hemispherical pore geometry used in that idealized model (see Figure 20, right) is fairly consistent with empirical (microscopic) observations, it appears in the theory as a strictly set parameter (Parkhutik and Shershulsky have initially noted in their work that the inner oxide boundary can be also modeled as a plane, and they assumed that the real cell bottom geometry is probably intermediate between a plane and a hemisphere).

An attempt to explain the hexagonal pore arrangement by the equalization of interpore distances was made in the “equifield strength model”, which also rests on the field strength inhomogeneity [139,140]. However, the authors reinforced their hypothesis by conducting only typical experiments using 0.3 M oxalic acid at 40 V and 15 °C, and no predictions on new self-ordering conditions have been made using this model yet. Hexagonal pore arrangement has been described using numerical methods as well (e.g., on Voronoi tessellations), but the reason for pore regularity was also not established [141,142].

In some cases, the same authors proposed several different options regarding the underlying reasons for the self-ordered PAA formation. A Moscow State University research team considered the pseudo-epitaxial PAA growth on aluminum substrates with a particular crystallographic orientation as the fundamental factor fully determining the long-range hexagonal pore ordering (this work is treated in detail in Section 4.3 of this review; see Figure 10) [101]. Later, the same research group suggested that the formation of long-range ordered PAA is controlled by two limiting ion transport processes: the best self-ordering can be achieved if the anodizing rate is limited either by migration in the barrier layer or by diffusion within the pores, whereas the mixed control regime leads to disordered PAA morphology [143]. None of those proposed mechanisms are particularly convincing at the moment, because multiple experimental investigations conducted by other groups led to conflicting conclusions regarding the role of the substrate microstructure (as comprehensively discussed in Section 4), and diffusion-limited processes are usually associated with the formation of dissipative structures that differ radically from hexagonally ordered PAA (such as fractal- or dendrite-like patterns during metal electrodeposition [6]). Honeycomb-like patterns on anodized metals are conventionally ascribed either to convective processes or to Marangoni-type instabilities caused by solute concentration inhomogeneities near the anode surface resulting in local variations of the film/solution interfacial tension [6]. Diffusion is driven by concentration gradients and should ultimately result in the equalization of concentrations at all points of the solution (thus, it makes rather a destroying effect and was correctly viewed by the Moscow group only as a limiting factor impacting the electrochemical reaction kinetics). Under certain conditions, diffusion may indeed have a structure-forming role and assist in the creation of a honeycomb-like pattern when properly combined with other (opposing) factors [16,17], but in order to clarify such a role in the proposed mechanism, the model may need further theoretical or phenomenological substantiation.

**Figure 21:**
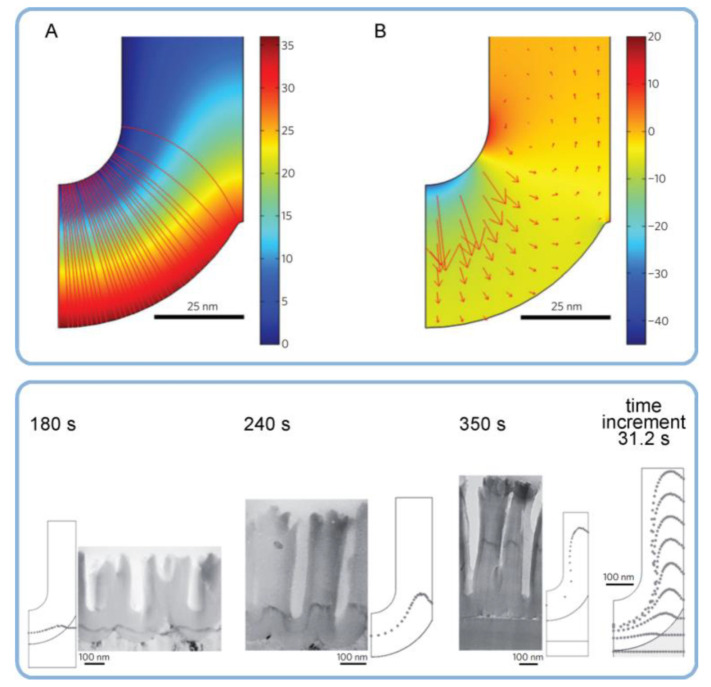
**Top**: the simulation results based on the viscous oxide flow model proposed by Houser and Hebert showing current lines and potential distribution (**A**) and velocity vectors and mean stress (**B**) during steady-state PAA growth. **Bottom**: a comparison of simulated tracer profiles from the work of Houser and Hebert and experimental ones taken from the earlier work by Skeldon et al. (Ref. [145]). Reproduced with permission from [144]. Copyright 2009 Nature.

In a model for the steady-state growth of PAA films developed by Houser and Hebert, the leading role in self-ordering was assigned to the tensile stress distribution in alumina and quasi-convective viscous oxide flow (Figure 21) [144]. This model was supported by an experiment demonstrating the diffusion of tracer elements in PAA from the pore base to the pore walls reported earlier by Skeldon et al. (see the images at the bottom of Figure 21) [145]. It should be noted that such observation is also fundamentally consistent with the electroconvective mechanism proposed elsewhere [16,17] and thus may lend experimental support to more than one theoretical model that have no severe contradictions between them (convection is usually defined as a collective flow of particles in fluids and gases, but not in solids, and viscous oxide should therefore presumably exist as a pseudo-fluidized medium; in the model presented in Ref [144], the authors have assumed that the oxide can be approximated as a Newtonian fluid, i.e., its viscosity *η* relates the local strain rate linearly to the viscous stress). The theoretical simulation performed by Houser and Hebert rests on a model where PAA represents a homogeneous Al_2_O_3_ continuum without anionic impurity. Thus, it does not address such characteristics of PAA composition as the colloidal nature of the amorphous films (which was reflected in some earlier concepts of colloidal self-organization, e.g., proposed by Murphy and Michelson or Thompson et al.), structural defects and flaws, bound water (up to 5.7%), and the electrolyte species content up to approximately 10 wt % (for more information; see Section 5 in this review) [55,111,112,136]. It is well known that the presence of grain boundaries and structural defects can significantly change the mechanisms and velocity of ion diffusion (the fast grain-boundary diffusion). However, it should be also noted that practically any mathematical model unavoidably implies some simplification and idealization, which therefore cannot be considered a weakness. 

Later, Hebert et al. explained pore initiation by the oxide dissolution chemistry and linked the conditions for hexagonal pattern emergence to such parameter as oxide formation efficiency (that is, the fraction of oxidized metal ions retained in the film: efficiencies for PAA growth were determined between 0.65 and 0.70, whereas compact “barrier” anodic films should be formed beyond this range) [108]. The authors assessed the previously suggested mechanisms based on nonlinear interface reaction kinetics (e.g., Parkhutik–Shershulsky theory [137]) as unrealistic and proposed to consider the nonlinearity of ion migration under the high-field conditions instead. The conditions for porous pattern formation in the new model by Hebert et al. were derived from the dynamics of the film/solution interface. The impact of oxide dissolution and ion migration on morphological stability was theoretically explored. According to this model, viscous flow considered in the preceding model by Houser and Hebert [144] is not important (damped) at the initial stage of anodizing, and pores initiate due to morphological instability, which becomes evident at an oxide thickness of a few nanometers. The critical film thickness above which viscous flow becomes important was related by the authors to the diffusion length scale, which constitutes about 10 nm for the anodic film. It was emphasized that this model neither addresses self-ordering during extended anodizing periods (as well as the related phenomena prevalent in thick films) nor considers point defects and incorporated electrolyte species in anodic layers. Given the well-known and precisely determined PAA growth rates (e.g., ≥33.3 nm min^−1^ for the experiments conducted in 0.3 M oxalic acid at 40 V [3,24]), it strikes the attention that the mechanism proposed by Hebert et al. appears to be valid only for the first 15–20 s of anodizing (for comparison, the steady-state regime of PAA growth is reached in 0.3 M oxalic acid only after approximately 6.5 min [91]).

### 6.2. The Theories Explaining PAA Formation by Electrohydrodynamic Convective Flows

Another class of theories that have continually evolved and matured over nearly a decade and a half and serve today as viable alternatives for the field-enhanced dissolution models is the theories explaining the growth of self-organized PAA structures by convective transport processes.

**Figure 22:**
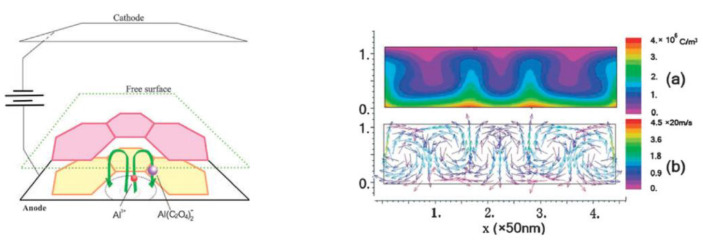
Schematic representation of the nano-convection process driven by electrostatic forces in the vicinity of the oxide/electrolyte interface assumed in the model proposed by Lu et al. (left image), and a numerical solution showing the density distribution of cations Al^3+^ and H^+^ and a vector plot of velocities ((**a**) and (**b**) in the right image, respectively). Reproduced with permission from [146]. Copyright 2009 The Royal Society of Chemistry.

Lu et al. have microscopically investigated very thin PAA layers obtained after 2 min of DC anodizing in 0.3 M H_2_C_2_O_4_ at 40 V and noticed that even shallow embryo pores exhibit some partial hexagonal ordering [146]. The authors have agreed with the previous explanations of self-ordering based either on the repulsive forces between neighboring cylindrical pores (due to aluminum/alumina volume expansion during oxidation) or on the “equifield strength model” [139,140,147]. However, such mechanisms seemed plausible only for long-term anodizing processes in which pores can grow deeper, whereas any possible pore–pore interaction at the very early stage of PAA formation must be very weak and thus could not adequately explain the hexagonal arrangement. Lu et al. have noticed that an array of hexagonal cells is one of the typical spatial patterns of convective origin and proposed their “ionic nano-convection model” for the early stage of aluminum anodizing. In that model, the initial ordering of nucleating pores was explained by an ordered pattern of charge distribution near the oxide/electrolyte interface formed due to electro-hydrodynamic convective flows. These flows are driven by electric force acting on the local excess charge in the portion of the fluid confined by the thickness of the diffusion layer (Figure 22, left). The ionic convection in the vicinity of the oxide/electrolyte interface was supposed to be induced due to the ejection of Al^3+^ ions into the electrolyte solution. However, polarization effects from such ions as Al(C_2_O_4_)^+^ or Al(C_2_O_4_)_2_^−^ were also considered in the model. The numerical solution of the model equations has shown alternating areas above the oxide surface where concentrations of cations and anions were periodically changed (Figure 22, right). The areas where cations (Al^3+^, H^+^) migrate away from the oxide/electrolyte interface under the influence of the electric field were termed “flow-up areas”, whereas anion migration in the opposite direction occurred in the “flow-down areas”. The pore formation takes place in the flow-up areas due to oxide growth inhibition combined with faster local dissolution. A higher concentration of ejected cations and a lower concentration of attracted anions at the flow-up sites result in a lower rate of oxide formation (it was supposed that anions migrate through the oxide layer to the metal/oxide interface, contributing to the formation of Al_2_O_3_). At the same time, lower pH values at the flow-up sites (due to the transfer of H^+^ into the solution) increase the oxide dissolution rate. Due to the complexity of the nonlinear governing equations in the model, the authors could not perform the conventional analysis based on the selection of proper Rayleigh numbers. Instead, the diffusion layer thickness was employed as a variable parameter to find solutions for a stable convective pattern. By analogy with the macroscopic Bénard cells whose characteristic sizes correlate with the thickness of a fluid layer in which thermal convection experiments are conducted [49], the PAA cell size was associated in the electroconvection model with the diffusion layer thickness. Lu et al. computed convection cell sizes for different applied voltages (in every case, the obtained solution corresponded to the best approximation for the diffusion layer thickness) and found a linear relationship between the PAA cell size and ∆*U* with a proportionality factor that closely matched the experimentally acquired value from the literature (≈2.5). Thus, the consideration of nano-convection enabled the authors to elucidate the relationship between experimental parameters that remained unexplained for a few decades.

In fact, the model reported by Lu et al. was built upon the earlier concept of the field-assisted oxide dissolution at the oxide/electrolyte interface. Nevertheless, it was an important turning point in efforts to understand the PAA formation mechanism. This study clearly demonstrated the exhausted explanatory ability of the earlier high-field conduction models (to which some scholars of that time still had an almost dogmatic adherence) and indicated the critical need for fundamental rethinking of the previous theories and consideration of new causative factors. It was an indirect admission that the classical models fail to explain one of their own basis elements—namely, the initial formation of the hexagonally patterned field inhomogeneity on the oxide surface, which leads to the pore etching at some selected regularly spaced points.

**Figure 23:**
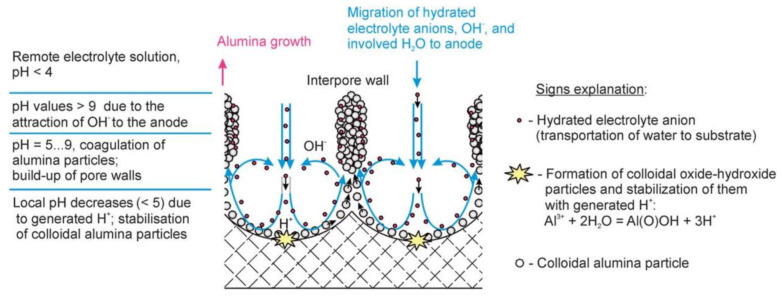
Schematic representation of the PAA formation mechanism according to the electroconvection-assisted colloidal self-organization model. Coherent motion of electrolyte ions and charge-stabilized colloidal alumina particles is responsible for the formation of equal-sized cells and the long-continued growth of PAA in the direction perpendicular to the metal surface. Different pH regions serve for stabilization of the colloidal suspension against aggregation very near the Al substrate and for the further coagulation and formation of polycrystalline interpore walls at a certain distance from the pore bottoms. Reproduced with permission from [16]. Copyright 2011 The Royal Society of Chemistry.

A decade ago, the author of the present review proposed the theory of PAA formation, which is fundamentally different from all previous models based on simultaneous dense film formation and its local field-assisted dissolution (including the model by Lu et al. discussed above) [16,17]. This theoretical model also differs from those models, in which an imaginary interface divides PAA into an upper porous layer and a lower compact, or “barrier-type”, layer (i.e., from the models that incorporate Hoar–Mott theory). In fact, this theory posits that no compact alumina layer exists below the pore base during PAA electrosynthesis. Instead, it considers all successive stages of alumina formation in an acidic environment from hydrated Al^3+^ cations to a stable solid mineral phase (see Section 5 and Figure 15 for details) and represents the “barrier layer” in situ as a slurry or a suspension containing charge-stabilized colloidal alumina particles that can participate in coherent motions responsible for the self-organization process. This fluidized medium is transported in the required directions by the ionic currents arising due to electroconvection. It flows from the middle of a PAA cell where aluminum is predominantly oxidized to the peripheral area where it forms the interpore walls and then gets solidified. The possible reasons for the colloidal suspension stabilization that facilitates such cooperative motion of “building blocks” followed by their rapid coagulation once pore walls are formed are thoroughly analyzed in Section 5 of this work—namely, these can be:

- The gradual evolution of hydrated alumina composition and structure (as it is transported from the pore middle to the pore walls) from complex polymeric cations to an electrocratic nano-colloid which is prone to rapid coagulation;
- The temperature gradient near the anode surface (due to Joule heating), which can cause variation in repulsion between colloidal alumina particles;
- The gradient in stabilizing electrolyte ions concentration (acid residues are attracted to the anode by Coulomb forces but are not consumed in RedOx reactions);
- A pH gradient due to generated H^+^ in close proximity of the anode surface (see chemical Equation (1)), on the one hand, and attracted OH^−^ from the direction of the bulk electrolyte, on the other hand (the concept proposed in the original paper where this theory was first reported; see Figure 23);

Thus, in this model, growing PAA is represented not as an initially compact oxide in which pores are etched at selected locations from above, but, rather, as a perforated (i.e., permeable for migrating electrolyte ions together with the entrained flow of surrounding fluids) honeycomb layer that practically “levitates” above the metal surface while being continuously supplied by new alumina “building blocks” arriving from below. Once such “building blocks” reach the base of an interpore wall, they can agglomerate into a new portion of solid alumina. Hence, the long-continued “vertical” PAA growth is supported (Figure 23). Once the applied voltage is switched off and the favorable conditions for a dynamic self-organization process accompanied by electrode reactions cease to exist, the formed PAA layer strongly adheres to the metallic substrate surface, and the remaining colloidal suspension rapidly sediments onto pore bottoms and coagulates into what we usually identify as a sealing “barrier layer” during ex situ microscopic investigations.

The fundamental difference between such model relying on the electroconvection-assisted colloidal self-organization and the earlier suggested dissolution-based mechanisms can be better formulated if we borrow the terminology commonly used in nanotechnology: whereas etching belongs to the category of so-called “top–down” methods in making nanostructured materials, the approaches based on the self-ordering of individual building blocks (e.g., atoms, molecules, or dispersed colloidal particles) into desired material are usually termed “bottom–up”. Therefore, the earlier theories propose a hybrid PAA formation mechanism in which equally essential bottom–up (the growth of compact alumina) and top–down (the field-enhanced dissolution) processes are combined. By contrast, in the electroconvection model, all basic steps (such as the nucleation and enlargement of colloidal alumina particles or their agglomeration to form pore walls) can be viewed solely as bottom–up, and the role of chemical dissolution in final pore shaping is considered to be only of secondary importance (e.g., for the formation of trumpet-shaped pore openings). Of course, drawing a complete analogy between the processes included into the convection-assisted PAA formation model and the bottom–up manufacturing approach in nanotechnology would be not entirely correct because the latter one is usually considered to be driven by the reduction of Gibbs free energy and movement of a system toward a state closer to a thermodynamic equilibrium state [96], whereas the spontaneous formation of dissipative structures such as PAA results from other causes. However, such comparative description still helps to better reflect the distinction between the basic ideas underlying different suggested mechanisms (although, it moves slightly away from the precise meaning of traditional terminology).

**Figure 24:**
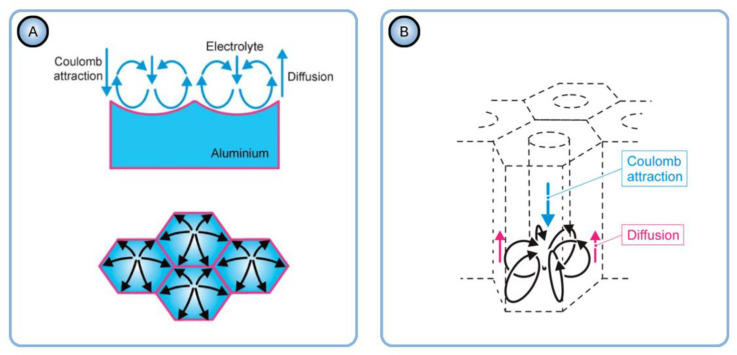
The principle of emergence of paired counter-rotating vortices above the Al surface and formation of a honeycomb-like pattern (**A**); the transport processes responsible for the continuous supply of water from hydrated electrolyte anions (the reactant) through the nanochannels, and for a stable “vertical” growth of interpore walls with regular spacing (**B**). Adapted with permissions from [16,17]. Copyright 2011 and 2013 The Royal Society of Chemistry.

The spatial distribution of cells formed on an anodized surface is determined by the self-organized pattern of electroconvective fluid flows. These flows originate from the complex dynamics of electrolyte anions under the influence of competing factors—namely, electromagnetic forces (Coulomb attraction) and the oppositely directed diffusion upon reaching a critical concentration gradient near the anode surface (Figure 24). As mobile electrolyte anions and entrained fluids migrate toward the anode, they can feel repulsion from the earlier accumulated negative charge near the solid surface and participate in complex viscous interactions. Thus, some recirculatory ionic and fluid flows (i.e., directed opposite to the main flow) can presumably occur already in the very beginning of the process. Under certain optimized conditions, counter-propagating ionic currents evolve into a system of ordered ring-like vortices, which can assist the formation of individual PAA cells (Figure 24A). Electrolyte anions are not consumed in electrode reactions but only lose water from their coordination shells (Equation (1)). Thus, they can act in the transport of water (the reactant) toward the Al anode continuously due to the cyclic convective transport mechanism and maintain a stable anodizing process (Figure 24B).

The network of temporarily existing vortex rings in the electrolyte solution is considered in such mechanism as the primary self-organizing structures. The remaining solid anodic structures on aluminum are only regarded as the fingerprints of these vortices that visibly appear upon the successful combination of some additional chemical factors.

**Figure 25:**
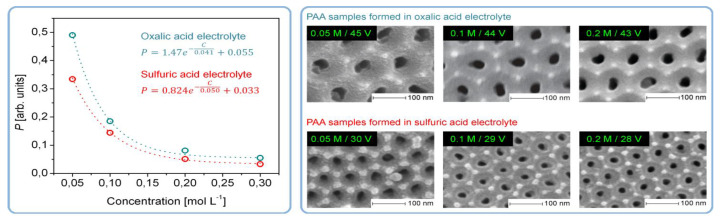
Left: exponential decrease in the critical values of the criteria *P* with increasing concentrations of H_2_C_2_O_4_ and H_2_SO_4_ anodizing electrolytes. Right: SEM micrographs of PAA layers obtained from both investigated types of electrolytes under the optimized anodizing conditions corresponding to the *P* values from the plot on the left (*P* and Δ*U* are related via Equation (4)). Adapted with permissions from [65,68]. Copyright 2016 The Royal Society of Chemistry and American Chemical Society.

In Pashchanka theory, the required anodizing voltage for self-ordering can be estimated using the empirically found electrochemical analogue of Rayleigh number (the criterion for transition from dense or disordered anodic alumina to well-ordered PAA):

(4) $$P=\frac{q_{\mathrm{av}}\Delta U}{\eta \sigma }$$

where Δ*U* is the voltage, *η* is the dynamic viscosity (cP), *σ* is the specific electric conductivity (mS/cm), and *q*_av_ is the average charge of supporting electrolyte anions (absolute value, *q*_av_ = 10^−pH^/*C*, where *C* is the known acid concentration). Thus, the criterion *P* is expressed in Equation (4) in terms of measurable macroscopic electrolyte solution parameters. The numerical value of *P* is non-constant for different electrolyte compositions and strongly depends, among other factors, on the concentration of charge carriers (or concentration and strength of a supporting electrolyte) [65,68]. In this regard, self-organized structures on anodized aluminum behave similarly to other known cellular patterns that arise due to electroconvection. For instance, such convective cells can be observed when an electric field is applied to a thin layer of non-reactive dielectric fluid [6,148]. For the calculation of a critical applied potential ∆*U* that is required for the occurrence of electroconvection in such systems, an equation very similar to the semi-empirical Equation (4) has been theoretically derived and experimentally confirmed [148] (that formula is not provided herein, but readers may consult previous work for a more detailed analysis of the similarities and differences between the two equations [65]). After the addition of even small amounts of conducting species to a dielectric organic fluid, the required voltage Δ*U* for the appearance of electroconvective patterns can drop from tens of kilovolts to a few volts. The corresponding critical value of the electrical Rayleigh number *Ra*_e_ (analogous to *P* from Equation (4)) also decreased by more than a factor of 2 (the measurements were performed for the systems with symmetrical or “bipolar” charge carriers injection) [6]. A similar situation exists with respect to the critical conditions for the self-ordering of PAA in aqueous electrolyte solutions. Two parallel studies of H_2_SO_4_ and H_2_C_2_O_4_ electrolytes whose concentrations varied between 0.05 and 0.3 M have confirmed that the critical value of the criterion *P* also systematically decreases upon the increase in acid concentration [65,68]. A drop in *P* from 0.334 to 0.033 for sulfuric acid solutions (under MA conditions at ≈0 °C), and from 0.490 to 0.055 for oxalic acid solutions was determined. As can be seen from the comparison in Figure 25, *P* values obtained for H_2_SO_4_ solutions lie lower than for H_2_C_2_O_4_ solutions with the same concentrations. Thus, it can be assumed that the strength of an electrolyte plays an important role, and the experimentally determined values of the criterion *P* should be related to the concentrations of charge carriers (ions) upon dissociation rather than the nominal acid concentrations. It should be noted that the overall concentration of charge carriers can be also affected by electrode reactions and the decomposition of water under given conditions. Due to such complex processes associated with the electrochemical synthesis in aqueous solutions, it is more difficult to isolate and separately investigate the major factors contributing to the onset of convective instability than in purely physical (chemically inert) model systems. At this early stage of theory development, the best way to make a reliable prediction of *P*-factor for a new unexplored electrolyte composition is to accumulate empirical data for already optimized similar systems (chemically related but differing by quantitative parameters) and to employ the interpolation/extrapolation method.

**Figure 26:**
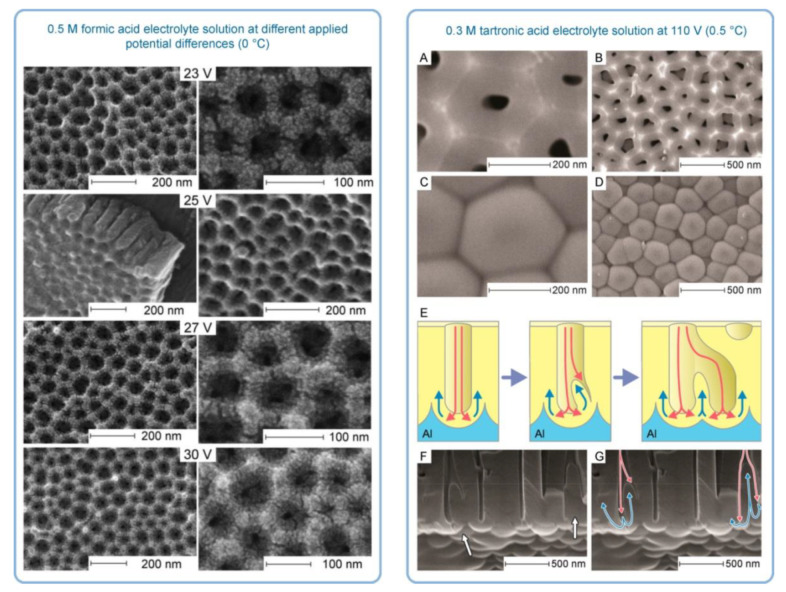
Left: PAA laminas formed in 0.5 M formic acid (HCOOH) solution under different applied voltages at 0 °C. Right: the porous side (**A**,**B**) and the barrier layer (**C**,**D**) of unsupported PAA laminas obtained from 0.3 M tartronic acid (HOOC−CH(OH)−COOH) solution at 110 V; schematic representation (**E**) and experimental observation in SEM (**F**,**G**) of meander-shaped protuberances leading to pore branching. The emergence of such protuberances and the formation of satellite channels is caused by the change from laminar to transitional flow mode upon small flow perturbations. Adapted with permissions from [16,62]. Copyright 2011 The Royal Society of Chemistry and 2017 American Chemical Society.

Despite the difficulties in the a priori determination of *P* values addressed above, Pashchanka theory has already successfully predicted PAA formation in two entirely new types of electrolytes—namely, formic and tartronic acid solutions, thereby proving its viability [16,17,62]. For this purpose, *P*-factors for the previously reported anodizing conditions that produce almost perfect PAA honeycomb structures were analyzed. Their values were found to lie at 0.057 ± 0.024, which was an efficient starting point in the prediction of the critical voltages above which self-ordering should also occur in novel and previously unexplored electrolyte compositions [16]. For each new acid, the suitability of the proposed anodizing conditions was confirmed by experiments. Both of these theoretically predicted new electrolytes were of considerable importance for further exploration of self-organized PAA. Formic acid (HCOOH) was the first monoprotic acid successfully employed for honeycomb-like pattern formation (Figure 26, left) [16]. Tartronic acid provided an opportunity to explore and determine the causes of pore branching and the formation of multiple pore openings within a single cell (Figure 26, right) [62]. This case study allowed connecting these two different types of defects to the occurrence of so-called “transient electroconvective turbulence” during PAA growth.

Moreover, an experimental validation of Pashchanka theory was performed by Stępniowski et al., who studied aluminum anodizing in aqueous oxalic acid solutions with added glycerol in different proportions (1–100 vol %) [81]. The authors have found that the average value of *P* estimated from the classical works on anodizing also suggested the best self-ordering conditions for such non-trivial experiments.

**Figure 27:**
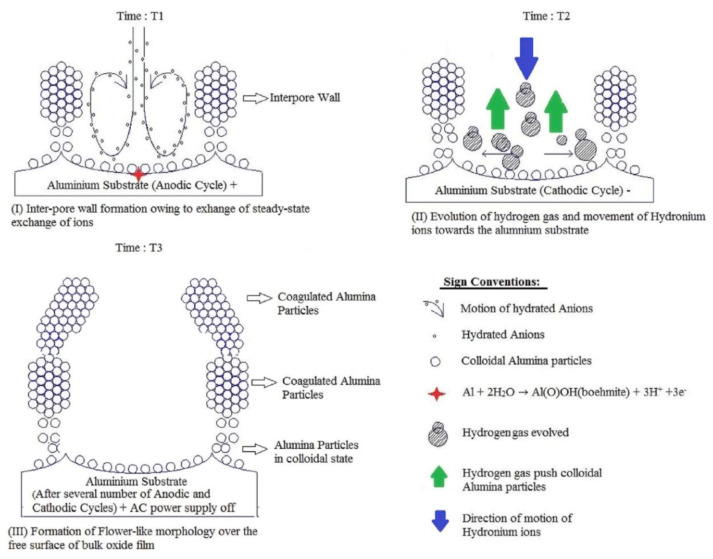
An extended model proposed by Chelliah et al. (based on the existing theory of electroconvection-assisted PAA formation) in order to include consideration of the processes occurring during AC anodizing. The additional mechanisms were suggested to explain the formation of flower-like structures microscopically observed on the surface of AC-generated PAA laminas. Reproduced with permission from [149]. Copyright 2017 Elsevier.

Later, Chelliah et al. compared morphologies of PAA laminas obtained in single-step DC and AC anodizing processes and discovered the evolution of localized flower-like structures on top of AC-generated PAA laminas, which could be also successfully explained by mechanism proposed by Pashchanka [149]. Chelliah et al. have suggested an extension to the convective model in order to include into consideration the processes happening during the interruption of steady-state anodizing by polarity reversal (see Figure 27). Once electrical current starts to pass in the opposite direction, the anode begins to act as a cathode and attracts H_3_O^+^ cations from the remote electrolyte solution into the cylindrical pores. Due to the local increase in the concentration of hydronium ions, colloidal alumina particles (that were formed in the previous anodic cycle) are stabilized against aggregation and maintain their mobility along the entire length of PAA nanochannels. Hydrogen gas bubbles formed at the pore base during the cathodic cycle (see Equation (5) later on in this text) can push these mobile stabilized alumina particles through the nanochannels and move them out of the bulk PAA layer. Chelliah et al. have assumed that these particles remain suspended just outside PAA during several anodic and cathodic cycles, and favorable conditions for their sedimentation are created upon turning off an AC power supply. Hence, flower-like structures are formed. It could be also envisaged that sedimentation periodically occurs during anodic cycles due to the attraction of OH^−^ anions and local increase in pH value at the top surface of PAA, leading to a gradual evolution of flower morphology. Chelliah et al. have also noticed that anodizing in 0.3 M H_3_PO_4_ at 100–130 V resulted in disordered PAA layers, while computations from Pashchanka theory predicted self-ordering at voltages exceeding 160 V [16,149]. Thus, the electroconvective model was consistent with the experimental evidence and provided theoretical support in a non-trivial situation (for alternating or pulsed current anodizing) where other models were limited in their explanatory abilities.

**Figure 28:**
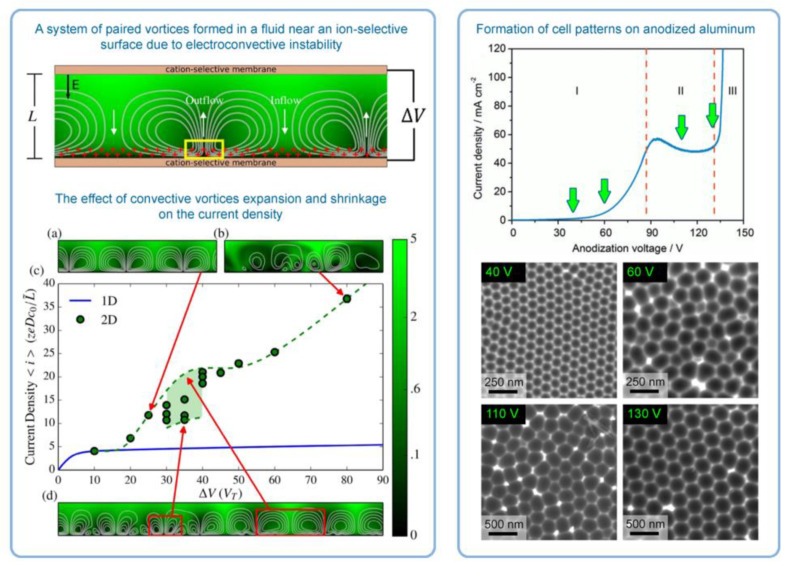
*j*–∆*U* curves and visualizations of cellular structures displaying similarities in morphology and behavior between different types of self-ordered electroconvective systems. Left: paired cyclic currents emerging in an aqueous electrolyte solution between two cation-selective surfaces when a certain critical voltage is applied (in this case ∆*U* ≥ 19 V); ∆*U* = 25 V corresponds to a stable regime with equal-sized vortices (**a**), ∆*U* ≈ 35 V corresponds to the beginning of chaotic mode where multiple vortex sizes coexist (shown by red frames in (**d**)), the plateau region between ∆*U* ≈ 35 V and ∆*U* ≈ 50 V in the *j*–∆*U* curve (the green dashed line in (**c**)) is a distinctive feature of such chaotic regime. Right: a *j*–∆*U* curve and SEM micrographs obtained upon Al anodizing in 0.3 M H_2_C_2_O_4_ solution (light green arrows show some selected voltages at which potentiostatic anodizing tests were performed); as follows from electron microscopy data, ∆*U* = 40 V corresponds to the self-ordering regime where uniform ring-like vortices assist the etching of a regular pattern (“mild anodizing”, MA); the system enters a disordered regime at ∆*U* ≈ 60 V, which further extends over the plateau region at 90–130 V (see the micrograph of a sample obtained at 110 V); electroconvection becomes more regularized and forms a new properly sized pattern at ∆*U* = 130 V (“hard anodizing” regime, HA); a slightly increased noise level on the *j*–∆*U* curve at intermediate voltages (≈ 100–130 V) is due to the enhanced turbulence of chaotic electroconvection (increased randomness of ionic flows causes observable electric current fluctuations). Adapted with permissions from [46] and [143]. Copyright 2016 Nature and 2017 Elsevier.

Knowing that PAA is a product of electroconvection helps to explain the nature of narrow voltage windows where the best self-ordering can be achieved. Such voltage ranges are related to anodizing regimes termed “mild” and “hard” (frequently abbreviated as MA and HA). The electroconvection theory provides a full and simple explanation of the intervals on *j*–∆*U* curves corresponding to the formation of well-ordered and disordered PAA layers (Figure 28). Common features of such curves covering the whole range of MA and HA anodizing potentials are briefly discussed in the introductory section of this review where the issues of evolution and nonlinearity of self-organizing systems such as PAA are addressed (see Figure 1). As demonstrated by Vega et al., the MA and the HA self-ordering intervals are separated by a current plateau region on the *j*–∆*U* curves within which no hexagonally ordered domains can be microscopically observed due to the rearrangement of pores and their readjustment for the HA conditions [5]. It has to be noted that the *j*–∆*U* curves strikingly similar to those reported by Vega et al. are common for many electrochemical systems in which the electroconvection helps to overcome the diffusive transport limitations [46]. Such systems also show characteristic current plateau regions where *j* does not increase with ∆*U* due to having achieved transport saturation. However, upon further increase in the voltage above a certain critical value, the so-called “overlimiting current” is observed, and *j* continues to increase owing to the electroconvective transport [46]. Understanding that PAA is a specific case of self-ordered structures of convective origin explains why this system enters a disordered regime with pore rearrangement between two self-ordering modes: while the large electroconvective vortices (corresponding to the larger HA cells in PAA) grow with the increased voltage, the initial MA nanoconvective cells shrink and become ineffective in charge transport. According to Pashchanka theory, controlling transport mechanisms under MA and HA regimes are qualitatively the same, but the current densities during hard anodizing have to increase dramatically because large-scale electroconvective flows can drive much larger currents. A comparison between two examples of self-organizing electroconvective systems (PAA and another type of cells that arise above ion-selective polarized surfaces and whose sizes can be analogously controlled by the applied voltage [46]) is presented in Figure 28 for a better demonstration of their common evolutionary patterns. As can be seen from Figure 28, both compared *j*–∆*U* curves exhibit characteristic transitional plateau regions due to the saturation of transport to a limiting current. This transitional regime can be characterized as “disordered” because small convective cells disrupt the formation of large ones. However, these larger cells grow in size as the applied voltage increases and ultimately form a new homogeneous pattern. At the same time, the initial small cells shrink under such conditions and gradually cease to exist.

**Figure 29:**
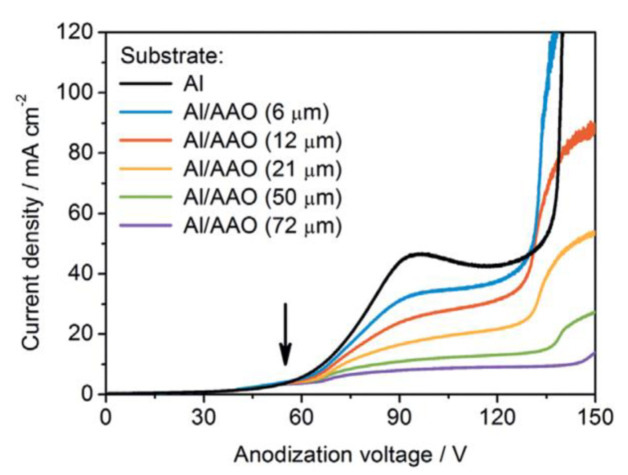
*j*–∆*U* curves obtained using two different types of Al anodes: a bare metallic foil near which the electroconvective vortical structures can freely change their sizes with progressively increased anodizing voltages (shown by the black curve), and preliminarily anodized Al foils (under MA conditions) near which the expansion of such vortices after passing the “mild” voltage range is limited by rigid pre-existing porous structures of different thicknesses (the corresponding curves are shown in different colors). The black arrow indicates the end of the MA range at ∆*U* ≈ 55 V. Reproduced with permission from [143]. Copyright 2017 Elsevier.

Such theoretical explanation of the transition between the MA and the HA modes has been confirmed by a superb experiment conducted by Roslyakov et al. (although the interpretation of results initially provided by the authors was different) [143]. In that work, *j*–∆*U* curves were recorded using different types of Al substrates: bare (not pre-anodized) and coated with PAA layers of different thicknesses (6–72 µm see Figure 29). These layers were grown prior to the analytical measurements using the MA approach (0.3 M oxalic acid, 40 V). Whereas pure Al foils demonstrated absolutely standard *j*–∆*U* curves within the entire range of tested MA and HA voltages (earlier reported by Vega et al [5]), the curves obtained using pre-anodized substrates coincided with the standard ones only under mild conditions below 55 V and diverged at higher voltages (the values of *j* were substantially decreased). A longer preliminary mild anodizing (i.e., a thicker PAA coating) resulted in a larger drop of current density above ∆*U* = 55 V during subsequent voltammetric measurements. In electroconvection theory, such behavior means that there must be factors suppressing the development of larger convective cells on aluminum substrates prepared under MA conditions. It is obvious that electroconvective cyclic currents confined within the rigid PAA structure can freely expand only up to sizes fitting the pore diameters. Thus, large convective vortices have no freedom for expansion due to such confinement, and electric currents remain restricted at higher voltages until an appropriate readjustment of propagating pore tip diameters is complete.

A brief comment should be also made as to the effect of the PAA layer thickness on the subsequent quenching of current densities above the range of “mild” voltages (see the different colored curves in Figure 29) [143]. The observed *j* values are related to the electrode reaction rates, which are directly associated with concentrations of reactants at pore bottoms. Therefore, the stagnation of measured *j* values indicates difficulties in the mass transfer of reactants to the metallic substrate (which also means hampered transport of the associated charge). Moreover, reduced Al oxidation rates extend the periods of time needed for the resizing of growing channels and, consequently, delay the process of PAA evolution driven by higher applied voltages. Thus, both the lower observed *j* values and broader and more flat plateau regions can be explained by retarded ion motion within PAA channels. This effect can be more pronounced for thicker MA-generated layers (as experimentally demonstrated by Roslyakov et al. [143]; see Figure 29) because they are characterized by larger distances over which the motion of ions is slowed down. That is to say, thicker films have longer nanochannel segments whose conductivity is considerably lower than in the bulk electrolyte. Such differences in conductivity can arise due to increased concentrations of different types of ions within pores, leading to the compression of the average separation of ions and stronger ion–ion interactions. For example, smaller distances between oppositely charged hydrated ions (acid residues, Al^3+^ etc.) enhance the viscous drag and decrease their drift velocity and therefore conductivity. Ions also tend to attract other ions of opposite charge into their “ionic atmosphere”, which is known to retard their motion (the phenomenon known as the “relaxation effect”) [89].

It is logical to raise a question about how the resumption of the nanoconvection process at the pore base (after passing through the current plateau region) can improve the overall conductivity of an entire high-aspect ratio nanochannel (up to several tens of micrometers in length) and make the “electrical breakdown” at higher voltages possible (see, for instance, the blue curve in Figure 29). The nonlinearity of *j*–∆*U* curves suggests that the synergy between electrode reactions and charge transport in the solution can be more complicated than it seems at first sight. It is known that a larger fraction of the total electric current is carried by the ions that have higher mobilities [89]. From this perspective, the largest contribution to the current must be from H^+^ ions due to their abnormally high ionic mobility. Protons are generated at the anode according to the half-reaction expressed by Equation (1) (or also Equation (8) later on in this text) and are rapidly transferred into the electrolyte solution to compensate their consumption at the cathode:

2H^+^ + 2e^−^ → H_2_↑. (5)

Thus, there is very likely a mutual dependency between the rates of electrode reactions and the electric conductivity along the entire length of PAA channels: while moving hydrated anions support continuing water oxidation at the pore base, the highly mobile charge carriers produced in such process make a significant contribution to the measured electric currents. Interestingly, OH^−^ anions are also characterized by rather high ionic mobility in aqueous solutions (this is why the reactions with their participation are also rapid, e.g., neutralization of acids and bases). Although the concentration of hydroxyls in acidic solution is very low, they can possibly also contribute to the replenishment of water (the reactant consumed in the anodic oxidation process) by fast migration through the pores and combination with protons.

## 7. New Directions for Theoretical Investigation and Flexible Design of PAA Structures Offered by the Electroconvective Model

In the previous chapter, the evolution of theories from field-assisted dissolution to electroconvection-based models was discussed. This chapter will be particularly focused on the recently proposed electroconvection-assisted colloidal self-organization model—namely, on the new avenues that it creates for further theoretical and experimental exploration of PAA formation [16,17]. Theoretical and experimental approaches frequently require different mindsets and different abilities that can be rarely found in one person (a vivid example of this is the exploration of thermal convection cells: the systematic study of convective flows was started by B.H. Bénard in 1900, but the first theoretical investigation was performed by Lord Rayleigh only in 1916). The main criticism of the electroconvective model of PAA formation currently concerns the semi-empirical character of the criterion *P*, which defines the critical conditions for porous structure appearance. Our updated knowledge about this critical number still strongly depends on the available measured data for different electrolyte compositions and on the experiments seeking information about related phenomena (as it happened with the recent discovery of the non-constant character of *P*: it appears that higher factors need to be used for more diluted electrolytes) [65,68]. Of course, even fundamental relationships do not necessarily need to be derived theoretically (for example, gas laws were first obtained experimentally and only later they were deduced by using the kinetic theory of gases, which confirmed the validity of this theory’s assumptions). However, formal modeling still can provide a valuable “sanity check” for the semi-qualitative theory. That is, it can test the correctness of the theoretical interpretation of empirical results by considering specific underlying phenomena. New results may reinforce the existing theoretical framework or extend it, but they may also suggest the necessity of a fundamental rethinking or can even disprove the model (in other words, if our knowledge about the studied process was not changed after the investigation, such investigation was pointless).

In this section, the first attempt of mathematical analysis of the nanoconvective model conducted very recently by Heinschke and Schneider will be thoroughly discussed, and an assessment of the validity of the obtained results will be provided. In addition, some possible future prospects of the experimental development and outlook for the fabrication of self-organized PAA layers with non-trivial cell morphologies will be provided.

### 7.1. First Attempts of Mathematical Investigation: The Heinschke–Schneider Model

**Figure 30:**
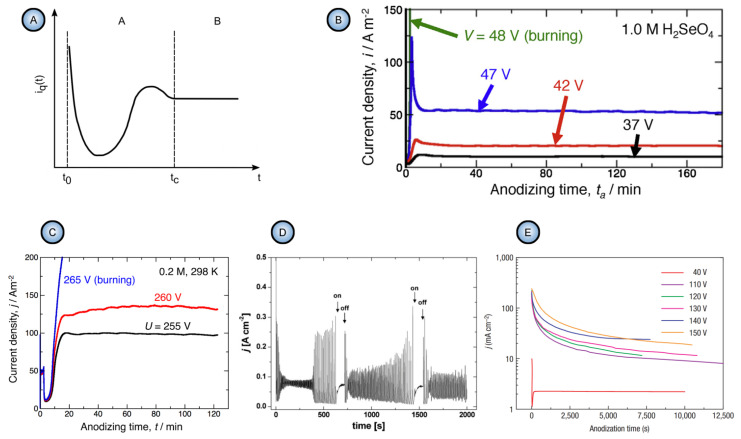
Schematic representation of a *j*–*t* curve adopted in the Heinschke–Schneider mathematical model as a basis for describing the general case of PAA formation (**A**), and various shapes of *j*–*t* curves which indicate the formation of highly ordered PAA structures in actual experimental situations (**B**–**E**). Although the curves obtained using a 1.0 M H_2_SeO_4_ solution are generally consistent with the model, electric current oscillations are visible at ∆*U* = 47 V (**B**). Nonlinear oscillating behavior becomes even more prominent when using 0.2 M etidronic acid electrolyte at ∆*U* = 260 V (**C**). The amplitude of oscillations increases during HA in unstirred 0.3 M H_2_C_2_O_4_ electrolyte (**D**), making it impossible to define the point *t*_c_. If HA is performed in a stirred 0.3 M H_2_C_2_O_4_ solution (**E**), the discussed mathematical model is not applicable due to the constant exponential decrease in *j* with time. Reproduced with permissions from [3,12,66,72,150]. Copyright 2020 American Chemical Society, 2014 and 2016 Elsevier, 2010 Wiley, and 2006 Nature.

**Figure 31:**
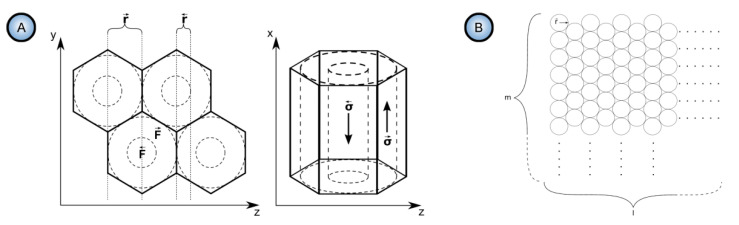
The geometry of an individual PAA cell (**A**), and the hexagonal PAA lattice resulting from the densest packing of ideal equal-sized circles on the anode surface (**B**), which appear in the model from Ref. [150] as initially set parameters. Reproduced with permission. Copyright 2020 American Chemical society.

**Figure 32:**
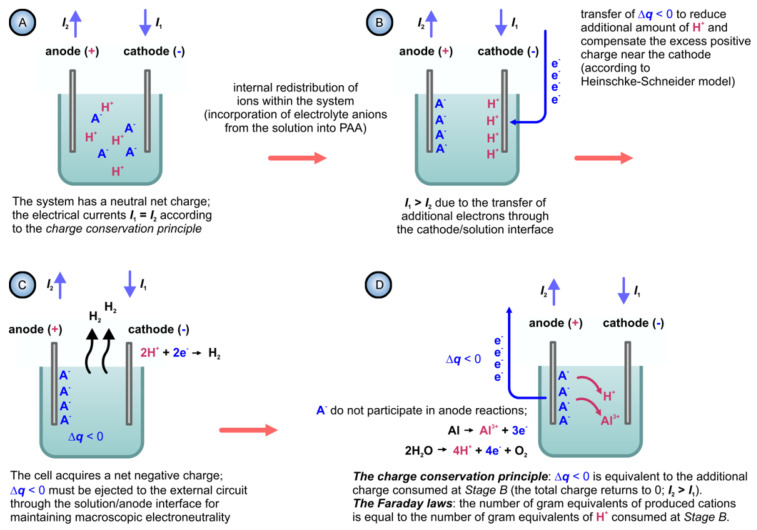
A set of the electrochemical processes that should occur in an anodizing cell (**A**) on the assumption that the incorporation of electrolyte anions into PAA leads to the reduction of an additional amount of H^+^ at the cathode (the excess positive charge compensation mechanism suggested in the Heinschke–Schneider model; see stages (**B**,**C**)). The supporting electrolyte is taken in this schematic diagram to be monovalent. It is evident that the complete sequence of all necessary RedOx reactions can only lead to the increase in Al^3+^ concentration and in pH (see (**D**)), but it cannot reduce the total positive charge carried by the different types of cations in the solution (if the process stops at stage (**C**), the electro-neutrality condition will be violated). Taking into account the macroscopic electro-neutrality of the entire system, the mechanism suggested by the authors of the mathematical model from Ref. [150] does not seem reasonable due to contradiction to the charge conservation principle and two Faraday laws (see (**D**)).

As already mentioned above, there are still many potential improvements that can be made to the electroconvection model. Although this theory is not purely phenomenological (because it does not simply establish relationships between empirical observations but also answers the questions “why?” and “how?”), the critical number *P* is not dimensionless in its current form and still awaits competently performed derivation from the fundamental physical relationships. Such mathematical justification could help verify that there is no hidden contradiction to the laws of nature, and that the theory is in agreement with the current scientific picture of the world. At the moment of writing this review, an attempt of formal analysis of the electroconvective model has been undertaken by Heinschke and Schneider [150]. Using some elements of Glansdorff–Prigogine thermodynamic theory (e.g., the modified “general evolution criterion”) [48], the authors have constructed a mathematical model in which the growth of PAA turns out to be migration-controlled, whereas solving the integrals for both the diffusion and the convection terms of the Nernst–Planck equation (which describes the transport of charged species in the absence of chemical reactions [105]) yielded zero. By introducing an abstract concept of entropy production from a single growing PAA cell, Heinschke and Schneider aimed to establish a theoretical connection between the macroscopic parameters of bulk electrolyte and the evolution of pores. The authors have also proposed an alternative expression for the criterion *P* that should better reflect its non-constant character, which was earlier established by experimental means [65,68]:

(6) $$P=2\frac{\Delta U}{\eta \sigma }\frac{I}{C}$$

where *σ* is the conductivity of the bulk electrolyte solution, *η* is the dynamic viscosity, *C* is the electrolyte concentration, and *I* is the ionic strength (thus, Equation (6) differs from Equation (4) by the replacement of 10^−pH^/*C* with 2*I*/*C*). The authors claim the universality and general validity of their model, and they also assert that it provides an accurate description of the experimental reality. However, a detailed examination unfortunately reveals that the Heinschke–Schneider model is rather fragmented and based on selective assumptions. Some of the criticisms can be listed as follows:

1. In the beginning of their theorizing, the authors state that: “The electrolytic system consists of two equal-sized rectangular aluminum electrodes (area *A*_r_). These are localized in an acidic electrolyte with a defined constant temperature *T*. Thus, the system is isolated against heat exchange with the environment.” Thus, this model contains an apparent confusion between *isothermal* and *adiabatic* conditions. Of course, nothing forbids heat exchange between an open system kept at a constant temperature and its surroundings (this is how, for example, different substances melt or evaporate), and the change of entropy in such cases equals to *Q*/*T*, where *Q* is the amount of heat (either gained or lost) and *T* is thermodynamic temperature. Moreover, since the Heinschke–Schneider model relies on Glansdorff–Prigogine theory, the words of Prigogine himself are relevant here: “The entropy produced by the irreversible heat flow leads to an ordering process, which would be impossible if taken independently from the heat flow.” [47]. Despite this known principle, Heinschke and Schneider wrote in their work: “The entropy production is characterized by the intrinsic entropy changes *d*_in_*S*/*dt* and the entropy gain by heat exchange with the surrounding environment *d*_en_*S*/*dt*. As the system is isolated with a bath that is held at constant temperature, *d*_en_*S*/*dt* is equal to 0.” [150]. By setting this condition, the authors have eliminated from their modeled system the critical factor that enables the formation of dissipative structures (i.e., the self-organization) as such.
2. In the introductory section of this review, the importance of the properly operating feedback loops as the necessary kinetic condition for spontaneous self-organization was discussed, and temporal oscillations were mentioned as a typical manifestation of the existence of such feedback loops [6]. The work by Lee et al. was also cited as an example of spontaneous electrical current oscillations observed during PAA synthesis under a potentiostatic regime (see Figure 2C,D) [12]. However, under some anodizing conditions, the amplitude of such oscillations and fluctuations can be decreased with time, so that they are no more easily recognizable on the *I*-*t* curves due to the limited sensitivity of standard laboratory equipment [91,92]. Such conditions are typically “mild”, but exceptions are also possible. In general, the oscillations are the result of the forces acting on ions, and the chances to observe them using the same electrolyte should increase with increasing applied voltage [66,72]. One of the cornerstones of the Heinschke–Schneider model is the existence of a critical moment in time *t*_c_ after which a stationary state is established and the electrical current (denoted in the work as *i*_q_(*t*_c_)) becomes constant in time. Thus, although the authors claim the general nature of their assumptions, their work in fact considers just one particular type of PAA formation processes in which temporal oscillations and fluctuations of the electrical current are not readily observable due to some reasons. Under experimental conditions similar to those reported by Lee et al. [12], when the amplitude of spontaneous current oscillations can even increase with time instead of decreasing, and self-organized PAA structures are still formed (actually, they even demonstrate a higher complexity in three dimensions), it would be absolutely impossible to determine the location of the point *t*_c_ dividing the whole process into the nonstationary and stationary phases in the interpretation of Heinschke and Schneider. Of course, the position of *t*_c_ could be simply chosen arbitrarily in such cases. In this connection, it would be again appropriate to cite here the words of Prigogine whose “general evolution criterion” was also used in the Heinschke–Schneider model: “Contrary to what happens at equilibrium, or near equilibrium, systems far from equilibrium do not conform to any minimum principle that is valid for functions of free energy or entropy production. As a consequence, there is no guarantee that fluctuations are damped. We can only achieve a formulation of sufficient conditions for stability, which we call the “general evolution criterion”. [47]. Thus, the flaw of the Heinschke–Schneider model seems to be fundamental because it relies on the very specific parameter *t*_c_, which in fact does not exist in a generic experimental situation, and it also does not formulate the sufficient conditions for stable PAA formation in a broader theoretical sense. Since Pashchanka theory served as a starting point for the authors’ theorizing, the reason for such a too-idealized representation of a typical *I*-*t* (or *j*-*t*) curve is not entirely clear. It is worth remarking here that it was mentioned in the original theoretical paper in 2011 that: “The competition of two driving forces (electromagnetic attraction against the diffusion) creates concentration fluctuations near the anode which lead to fluctuations of current with time… This results in damped oscillations of the *I*–*t* curve until the equilibrium is established. Only one local maximum in this oscillation has been observed for a long time (presumably due to the restricted sensitivity of measuring equipment). However, damped oscillations are already reported …” [16]. Thus, the oscillatory behavior of *I*-*t* curves (amply documented by now under different experimental conditions) cannot be considered as non-relevant, but they could be envisaged using the originally proposed convection-based mechanism. In light of contemporary evidence (including the advances made since 2011), the using of constant electric current as the basis for a new model does not seem well founded and can be even considered a step backward. It should also be taken into consideration that the shapes of anodizing curves recorded in some experimental situations are very different from the example demonstrated in the Heinschke–Schneider model (irrespective of whether or not oscillations and fluctuations have been detected; see Figure 30). For instance, current–time transients for HA using 0.3 M oxalic acid solution show a continuous decrease in current density with time, i.e., defining the position of a critical moment *t*_c_ for such curves is evidently impossible (Figure 30E) [3].
3. The Heinschke–Schneider model considers only two half-reactions within the entire system: reduction of protons at the cathode expressed by Equation (5) and oxidation of aluminum at the anode:
  Al → Al^3+^ + 3e^−^. (7)
  The authors write that such two reactions “ensure the electron transfer between the electrodes” and that “no other reactions related to the described irreversible process are considered.” [150]. It is not clear why the reaction of water oxidation, which is essential to the PAA formation process and whose contribution to the ionic current between two electrodes is much more significant than that one from Al^3+^ ions, was not included into this model. Water oxidation is incorporated into Equation (1), and it can be separately written as:
  2H_2_O → 4e^−^ + 4H^+^ + O_2_. (8)
  Disregarding the water oxidation process further leads to the next incorrect assumption in the Heinschke–Schneider model: near the anode (denoted as the position *x*_0_ in their one-dimensional coordinate system), the concentration of H^+^ is minimized because of repulsion forces.
4. The authors of the model considered the criterion *P* to be directly related to the entropy production and also “associated with the charge resulting from the Al oxidation reaction.” They made the following assumptions: “the majority of ions flowing toward the bulk are assumed to be Al^3+^ ions and anions. Here, only the former ones represent a local excess charge… Thus, only Al^3+^ ions are mentioned further for the determination of ion flow toward the bulk and the entropy production related thereof.” At this point, it is useful to clarify that hydrated ions ejected into an aqueous solution can contribute to the entropy production not because they are formed or because they are charged but because they are moving. The entropy of formation of hydrated Al^3+^(aq) ions is negative (relative to hydrogen ions): ∆_for_*S*° = −321.7 J∙K^−1^∙mol^−1^ [151]. However, from general considerations, the motion of hydrated ions through the solution should increase the entropy due to frictional forces and viscous dissipation (the production of entropy in this case equals to the Rayleigh dissipation function *R* divided by absolute temperature *T*). From this standpoint, the motion of hydrated electrolyte anions (acid residues) should make a much larger contribution to the production of entropy, whereas the effect of small concentrations of Al^3+^ ions should be only minor. It should be mentioned here that the formation of hexagonal patterns due to charge carrier currents coupled with fluid flows can be also observed in purely physical systems without any chemical reactions. Thus, the Al oxidation reaction can be only of secondary importance for the emergence of self-organized flows in this case.
5. Heinschke and Schneider are using idealized models of a primitive PAA cell (two coaxial cylinders of diameters corresponding to the pore and cell sizes; see Figure 31A) and of the hexagonal lattice (composed of ideal circles; see Figure 31B). Since these authors have eliminated the diffusion and convection parts of the ion flux from the model and left only the migration component, it is not clear which mechanism is responsible now for the formation of such ideal pore morphology and arrangement. The theory of self-organization requires specifying the mechanism of irreversible processes because the laws of nature in a state far from equilibrium become mechanism dependent [47]. Thus, proposing a new hexagonal pattern formation mechanism after the migration transport has been assigned a leading role in the pore formation would be highly desirable. By employing hexagonally arranged cylindrical pores as the initially set condition without any explanation of their emergence, the Heinschke–Schneider model practically maintains the same approach as was used in the Parkhutik–Shershulsky model in 1992 and later criticized by some other authors as unfounded [91,137].
6. In the introduction section of this review, special attention was paid to the nonlinearity of self-organizing systems. Ohm’s law is not met in a cell for PAA synthesis, and the electrical current does not linearly scale with an applied voltage (i.e., the electrical resistivity or conductivity are not constant, according to the character of *j*–∆*U* curves and possible oscillations of *j*–*t* curves) [5]. Nevertheless, Heinschke and Schneider have used Ohm’s law in their model to calculate the specific conductivity of a small volume of electrolyte directly at the cathode (denoted in that work as *σ_λc_*). No special reservations as to why this should be possible in a nonlinear system were made.
7. Heinschke and Schneider have also addressed the issue of incorporation of electrolyte anions from the bulk solution into PAA. The authors state that: “To compensate the excess positive charge which evolves by the loss of anions in the bulk, an additional amount of H^+^ have to be reduced at the cathode.” [150]. It can easily be demonstrated that such statement is unfortunately misleading. Let us consider a system consisting of two electrodes immersed into an electrolyte solution. Since such a system obviously stays macroscopically electro-neutral during the entire anodizing process, the electrical current passing through the cathode/solution interface must be equal to the current passing through the anode/solution interface (see Figure 32A). This is dictated by the charge conservation principle: if the total charge within the cell remains equal to 0, the amount of charge flowing into the cell must be equal to the amount of charge flowing out of the cell. Now, let us follow the considerations of Heinschke and Schneider and suppose that some internal redistribution of ions within such cell (e.g., transfer of anions from the solution into the solid film on the anode) indeed results in the reduction of an additional amount of H^+^ at the cathode. This would require an additional amount of electrons transferred from the external circuit into the cell through the cathode (Figure 32B). Due to the charge conservation principle, an equivalent amount of charge must leave the cell through the anode/solution interface (Figure 32C). This is only possible through electrode reactions summarized by the half-equations 7 and 8 (see Figure 32D): according to Faraday laws, the amounts of additionally produced H^+^ and Al^3+^ ions at the anode will be proportional to this additional charge leaving the system to maintain its electro-neutrality (both Faraday laws are summarized as *n* = *Q*/*F*, where *n* is the number of gram equivalents of a reacted substance, *Q* is the charge that passed through the interface, and *F* is the Faraday constant). To sum up, the consumption of an additional positive charge at the cathode (see Figure 32B) will result in the production of an equivalent amount of the same charge at the anode (see Figure 32D), and the mechanism of charge reduction proposed by Heinschke and Schneider is invalid because it violates the charge conservation principle and two Faraday laws at the same time. In addition, if the transfer of anions into PAA is supposed to create excess positive charge within the solution, the PAA layers must be charged negatively for the sake of electro-neutrality of the entire system. No mechanism for the negative charge compensation in PAA was proposed in the Heinschke–Schneider model. As was discussed in Section 5.3 of this review, the actual mechanism of anionic impurity incorporation into PAA is very likely akin to ion exchange when electrolyte anions simply replace OH^−^ ions within PAA (see Figure 16). Thus, no excess charge is created within the solution, and therefore, no electrode processes need to be additionally involved.

To sum up, there are numerous convincing arguments that the Heinschke–Schneider model requires a fundamental rethinking (such as missing half reactions leading to an imbalanced RedOx equation of the entire process, over-idealized representation of an *I*-*t* curve obtained in a generic MA or HA potentiostatic anodizing process, contradiction to the charge conservation principle and two Faraday laws, or an apparent misconception regarding the nature of self-organizing systems). Unfortunately, the authors were not thorough and just stressed significantly on secondary factors without due considering the primary factors that have already been previously reported. Once primary factors are considered to their existing mathematical model, the resulting conclusions become rather arbitrary. Thus, further theoretical efforts toward addressing this issue are needed.

However, it should be also mentioned that the Heinschke–Schneider model already had some resonance and was rather well-received by Ruiz-Clavijo et al. (Madrid research group) and mentioned in the very recent comprehensive review paper about alumina templates [45]. Thus, it will be still interesting to follow the progress along this new line of investigation.

### 7.2. Possible Prospects for Experimental Study of PAA Formation: Toward the Smallest and the Largest Achievable Cell Sizes and Non-Trivial Morphologies of Anodic Alumina Nanostructures

The electroconvection-based theory opens numerous prospects for further study of PAA morphological tuning. It casts a new light on such intriguing issues as the smallest and largest PAA cell sizes achievable through a spontaneous self-organization process, as well as new types of periodic anodic structures that can be possibly generated upon some radical changes of anodizing conditions. Some considerations on those issues are presented below.

#### 7.2.1. The Smallest Achievable Cell Size in Self-Organized PAA and its Relationship to the Debye–Hückel Length

There is sufficient reason to think that the smallest achievable cell sizes (or interpore distances) in well-ordered PAA correlate with the Debye–Hückel screening length *λ*_D_, which is a specific property of a particular electrolyte solution:

(9) $$\lambda_{D}=\sqrt{\frac{\varepsilon RT}{2F^{2}I_{c}}}$$

where *ε* is the electrical permittivity; *R* is the universal gas constant; *T* is the thermodynamic temperature; *F* is the Faraday constant; and *I*_c_ is the ionic strength of the bulk solution. The electrostatic attraction between the oppositely charged “upstream” and “downstream” ionic flows that assist the formation of an individual PAA cell is prevented due to the response of dipole H_2_O molecules and dissolved ions to the local electric fields, or, in other words, due to the Debye–Hückel screening effect. Therefore, the counter-propagating flows are separated by a characteristic length scale comparable to the Debye–Hückel screening length *λ*_D_, which is typically between 1 and 100 nm for most of the electrolyte solutions and correlates well with the commonly obtained PAA cell dimensions [105]. The screening effect prevents the intermingling of the ionic and fluid flows and ensures their spatial separation, which is necessary for the appearance of well-defined nanoconvective cells and their periodic arrangement. According to the reviewed literature, the smallest periodicity of nanostructures on anodized aluminum which can be achieved via spontaneous self-organization is about 25–30 nm [59]. An artificial pre-patterning method (using a 2D array of *γ*-Fe_2_O_3_ nanoparticles) allowed compressing the spacing between pore nucleation sites down to 13 nm, but high-aspect-ratio nanopores are difficult to obtain by this approach without the loss of their hexagonal arrangement [94]. Since particular attention in nanotechnology is paid to size effects, it would be interesting to explore further opportunities for decreasing cell dimensions in self-organized PAA. Now, it is worthwhile to analyze and simplify Equation (9) and to search for a possible valid type of correlation between the PAA cell dimensions and controllable variables in an electrolyte composition. If the temperature *T* is fixed (the cell is thermostatically controlled), the constants *RT* and 2*F*^2^ can be moved outside the radical and included into the proportionality constant between the interpore distance in PAA (*D*_int_) and λ_D_. Thus, the smallest possible *D*_int_ should increase with the electrical permittivity *ε* and decrease with the ionic strength *I*_c_:

(10) $$D_{\mathrm{int}}\propto \sqrt{\frac{\varepsilon }{I_{c}}}$$

**Figure 33:**
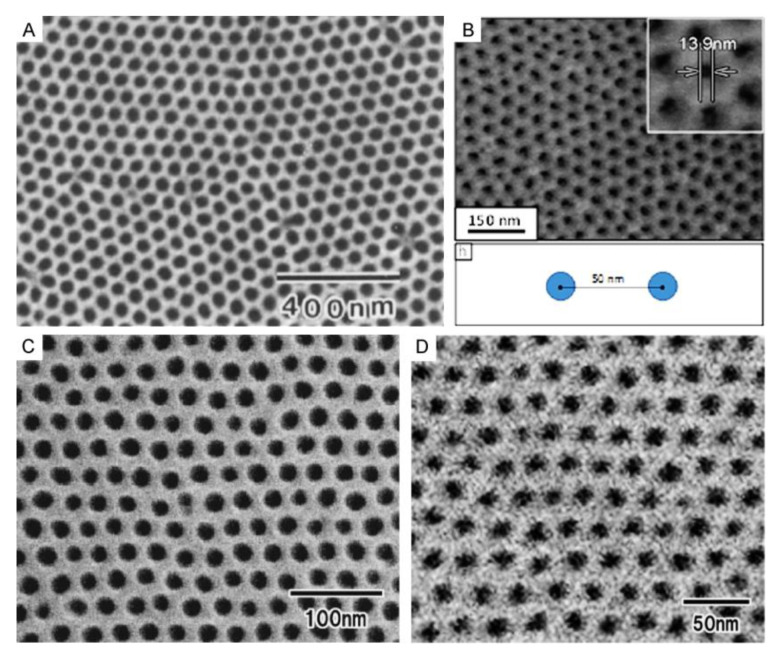
(**A**) A self-ordered PAA layer with ≈63 nm periodicity formed in 0.3 M H_2_SO_4_ solution at 0 °C and ∆*U* = 27 V (see Refs. [2,69]); (**B**) Interpore distances reduced down to ≈50 nm by modifying the electrical permittivity of the electrolyte solution (10 wt % H_2_SO_4_ with 50 wt % of added ethylene glycol, 0 °C, ∆*U* = 19 V; see Martín et al., Ref. [83]); (**C**,**D**) A further decrease in the spacing between pore centers down to 30–25 nm can be achieved by using extremely high 8 M and 9.4 M H_2_SO_4_ concentrations at 40–60 °C (Masuda et al., Ref. [59]). Reproduced with permissions. Copyrights 1997 The Electrochemical Society, 2012 American Chemical Society, and 2006 The Japan Society of Applied Physics.

For example, if we increase the concentration of the bulk electrolyte, the electrical permittivity *ε* will drop (for concentrations below 2 M, the following dependency is known: *ε*_solution_ = *ε*_water_ − 2*δC*, where *δ* > 0 is the temperature-dependent parameter unique for every electrolyte, and *C* is concentration [152]), and *I*_c_ will rise (because equilibrium concentrations of anions will increase and their valences stay unchanged). These considerations explain why the synthesis of PAA with smaller interpore distances requires larger concentrations of an electrolyte (e.g., Masuda et al. were able to achieve *D*_int_ values of 25–30 nm upon increasing H_2_SO_4_ concentration for pre-anodizing up to 8–9.4 M; see Figure 33C,D) [59]. This universal regularity is confirmed by a number of experimental facts: for example, self-ordering conditions optimized for 0.3 M and 1.07 M (10 wt %) H_3_PO_4_ solutions produce *D*_int_ equal to 500 and 420 nm, respectively (see Table 1) [8,50]. In addition, self-ordering in 0.3 M H_2_SO_4_ yields *D*_int_ = 63 nm (Figure 33A), while the reduction of the electrical permittivity by using more concentrated 10 wt % solution with added ethylene glycol (*ε* = 41.1 at 20 °C) makes it possible to decrease the interpore distance down to 50 nm (Figure 33B) [2,69,83,84]. However, there has not yet been any dedicated experimental study clarifying common patterns (including possible nonlinear effects) upon increasing concentrations of different types of electrolytes. Extremely concentrated acids can be the source of numerous experimental difficulties (very large electric currents and required powers, enhanced chemical dissolution, a strong possibility of “aluminum burning”, etc.). Thus, such an approach to PAA with smaller *D*_int_ can be considered only with a number of serious reservations.

#### 7.2.2. The Largest Possible Cell Sizes on the Anodized Aluminum Surface: The Multiscale Nature of Electroconvection

**Figure 34:**
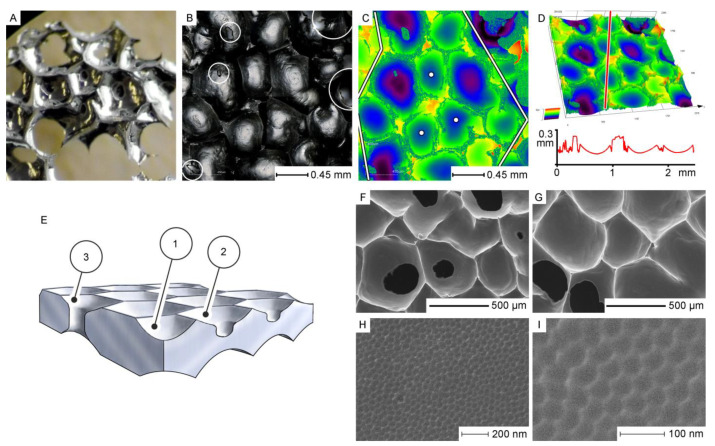
Formation of hierarchical anodic textures on aluminum (in one step using a 0.3 M H_2_SO_4_ solution at 40 °C) demonstrates a complex and multiscale nature of chaotic electroconvection. Polygonal sub-millimeter pores on the upper hierarchy level (**A**–**G**) have either hemispherical (labeled as **1** in the schematic diagram (**E**)) or funnel-shaped (**2** and **3** in (**E**)) bottoms, and a tendency to hexagonal coordination (see the optical microscopy image in (**B**) and the corresponding pore topography measurements obtained by the non-contact 3D visualization method in (**C**,**D**)). The cells inside the boundary in (**C**) are hexagonally packed (every pore surrounded by six nearest neighbors is marked with a white dot). High-resolution SEM images (**F**–**I**) reveal a hierarchical structure in which two honeycombs with cell sizes differing by a factor of 10^4^ are superimposed. Reproduced with permission from [54]. Copyright 2020 The Royal Society of Chemistry.

By definition, convection is a macroscopic collective flow of particles in a fluid. The coherent movement of such particles within an electrolyte solution is possible at various length scales, and the etching of larger pores is connected to the enlargement of turbulent vortices (counter-rotating convection rolls). Thus, the achievable pore dimensions should be mainly restricted by the energy flowing through the dissipative system (by means of application of voltage and elimination of heat) and the size of an electrochemical cell. This assumption has been recently justified by experiments conducted in a 0.3 M H_2_SO_4_ solution maintained at the temperature of 40 °C (Figure 34) [54]. The polygonal cells formed on an Al surface under such conditions were morphologically similar to the conventional nanoscale honeycomb textures, but their dimensions increased to 0.45–0.90 mm (i.e., by a factor of 10^4^ in comparison with pores generated in a cooled solution; see Figure 34A–G). Although these first experiments still require much optimization to improve sub-millimeter pore uniformity, they showed that the etching of pores beyond the habitual nanometer and sub-micrometer scale ranges is possible.

#### 7.2.3. Anodic Alumina Nano- and Microstructures of Unusual and Complex Shapes

**Figure 35:**
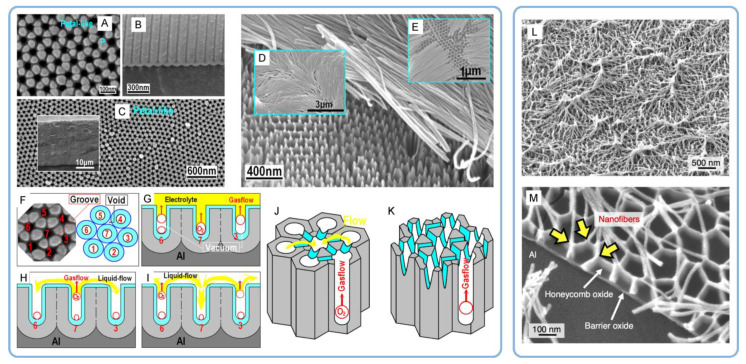
SEM images of PAA layers obtained from a 3 wt % H_2_C_2_O_4_ solution at 4 °C and 40 V which show a petal-like micro-pattern on the porous side (**A**–**C**), and anodic alumina nanofibers synthesized using 6 wt % H_3_PO_4_ at 20 °C and 80 V (**D**,**E**). A formation mechanism based on the “plastic flow” and the “oxygen bubble mold” models (**F**–**K**) has been proposed for these two new types of structures by Fan et al. (Ref. [154]). Kikuchi et al. (Ref. [11]) have reported the use of concentrated pyrophosphoric acid solution at 20 °C and 75 V for the synthesis of a similar type of anodic alumina nanofibers (**L**–**M**). The results presented in Refs. [11,154] suggest that the range of suitable anodizing conditions for the formation of alumina in such nanofibrillar form can be possibly further expanded. Reproduced with permissions. Copyrights 2014 Nature and 2017 Elsevier.

Although nanoscopic cells in well-ordered PAA usually tend to have the shape of a hexagon, there is a variety of other configurations of convection cells that can be potentially achieved under different experimental conditions (this refers especially to the thermal Rayleigh–Bénard convection but can be generally valid for pattern-forming systems differing in their nature) [153]. The following types of patterns can be mentioned as most frequently observed in real experimental situations: parallel rolls (e.g., nearly straight or S-shaped), hexagons of *l*- or *g*-type (that is, either with upward or with downward fluid flows in their centers), square cells, or an array of various polygons formed under non-optimized (transitional) convection regimes. Transformations between such different patterns are, in certain circumstances, also possible. For instance, conventional hexagonal cells can be considered as a product of evolution of parallel convective rolls when three systems of such rolls are superimposed at an angle of 120 degrees. Whether one type of formed patterns is preferable to another depends on a number of factors. For example, fluids that have different viscosity behavior (that is, dependence on temperature, inhomogeneity along the “vertical” axis, etc.) can demonstrate different convective structures under similar experimental conditions. The presence of suspended solid particles is another important parameter that can significantly alter the mechanical properties of a fluid: while polygonal Bénard cells are preferably formed in highly concentrated suspensions, rolls are formed in fluids with low concentrations of such particles, other factors being equal [153]. Since anodic alumina originates as slurry [16], and because properties of an electrolyte solution within nanochannels are inhomogeneous, it can be assumed that these considerations are also valid for the electroconvection-assisted PAA formation mechanism. It is noteworthy that different PAA cell geometries are potentially beneficial in many areas (e.g., square and triangular lattices may find application in photonics or template-based synthesis), and corresponding tools for the artificial laying-out of pore initiation sites have already been developed [142]. However, the spontaneous formation of diverse non-trivial alumina architectures still remains rather unpredictable and poorly understood. Although the emergence of new morphologies due to radical changes in the structure of convective flows (which can result from even very small changes in physical settings) look fairly possible in theory, their experimental confirmation seems to be rather elusive. Recently, Fan et al. have reported the formation of alumina petal-like structures and nanofibers upon two-step anodizing of electropolished Al sheets in 3 wt % H_2_C_2_O_4_ and 6 wt % H_3_PO_4_, respectively (Figure 35A–E) [154]. The authors have mentioned that these morphologies could be explained neither by the field-assisted dissolution nor by the plastic flow model (even the physical meaning of such terms as “field-assisted” and “dissolution current” were called into question). The explanation proposed by Fan et al. was based on a combination of the plastic flow model in which oxide is supposed to be displaced upwards to form pore walls and the “oxygen bubble mold” model that implies the presence of a spherical gas bubble at the pore base around which oxide should flow (that is, the movement akin to electroconvective vortices was modeled in a more elaborate way) [155,156]. The formation of the new structures was rationalized as follows: if oxygen pressure within a pore exceeds the ambient pressure, the gas will move “upwards” from the pore base and entrain a “micro-liquid-flow” (Figure 35G). Once such transient upward flow comes out of a pore opening, it can intersect and merge with the downward flows directed into the neighboring pores (Figure 35H–I). Hence, short bridging flows appear between the neighboring pores and assist with the etching of grooves on alumina (Figure 35J,K). Of course, such explanations of complex dynamic processes on nanoscale are frequently based on guessing and cannot be sufficiently supported by analytical methods (Fan et al. have mentioned in their work that oxygen bubbles in nanopores are hidden from direct in situ observation) [154]. From that standpoint, if such transient upward flows within nanopores indeed exist, the short-term convection of *l*-type at pore bases during an unstable PAA formation process can be equally considered to be a viable explanation. For example, in the hypothetical case that all convective cells are of *l*-type, an array of separated alumina columns (or high-aspect-ratio wires) would be formed instead of a porous monolithic layer (interest in such structures is also reflected in the literature [157]). That means that reversals in nanoconvective ionic flows could probably result in the inverted PAA structure. It should be also mentioned that fiber structures similar to those reported by Fan et al. have been observed by Kikuchi et al. during the potentiostatic anodizing (∆*U* = 75 V) of Al sheets performed in pyrophosphoric acid (H_4_P_2_O_7_) solution after galvanostatic pretreatment (Figure 35L–M) [11]. Kikuchi et al. have demonstrated that alumina nanofibers originate at points where three neighboring PAA cells are adjacent (i.e., where three upstream flows are superposed and can make their contribution to the accelerated vertical growth of alumina structures, according to the electroconvective model). Thus, we can speak of a new class of anodic materials that are achievable using different electrolytes, but we must also bear in mind that their formation can be assisted by yet unknown mechanisms.

**Figure 36:**
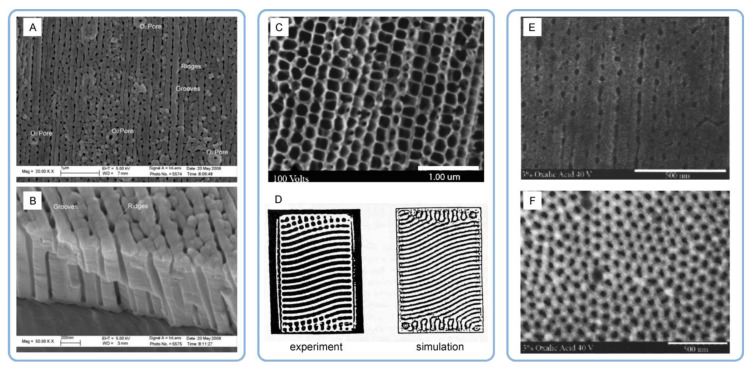
(**A**,**B**) Pores in PAA organized in parallel rows instead of the habitual hexagonal arrangement (the synthesis was performed in a 6 wt % H_3_PO_4_ solution in ethylene glycol under galvanostatic conditions; see Ref. [156]); (**C**) Nearly square PAA cells packed in rows obtained from 5% H_3_PO_4_ at 100 V (Ref. [158]); (**D**) A similar macroscopic pattern observed during a thermal Rayleigh–Bénard convection experiment performed in a container with unequal side lengths (two images correspond to a photograph and a numerical simulation; see Ref. [153]); (**E**,**F**) The difference in pore arrangement obtained after a single-step (**E**) and after a two-step (**F**) anodizing process using 3% H_2_C_2_O_4_ solution at 40 V (Ref. [161]). Reproduced with permissions. Copyrights 2008 Springer, 2007 AIP Publishing, 1998 World Scientific Publishing.

Another very interesting type of PAA laminas frequently reported in the literature has pores arranged in rows and not in honeycombs (Figure 36) [156,158,159,160]. On the one hand, such parallel lines of pores could be assigned to roll patterns crossed by perpendicular convective rolls of smaller amplitude (so that individual pores can be separated but remain organized in rows; see Figure 36D) [153]. On the other hand, such PAA structures are practically always observed on rough aluminum substrates without prior electropolishing, and the impact of the grooves on the mechanically flattened or polished (rolled) metal surface on the development of PAA lattice morphology can be very pronounced. If so, the next question would be about the ratio of sizes of PAA cells and the grooves on polished aluminum: can such mechanically created depressions on the metal surface serve as relatively large nanocontainers whose sidewalls dictate a particular regime of convective flows, which causes the formation of such unusual arrangements of pores? [153]. It can also be observed that pores become rearranged into a more typical pattern upon a two-step anodizing procedure, which leads to an even pre-structured surface of Al substrates (Figure 36E,F) [161].

In summary, the correct interpretation of experimental findings is often complicated by a very large number of possible contributing factors, which gives rise to the need of constant improvement of our understanding of PAA formation mechanism and prompts the search for new plausible explanations. However, rapid progress in solving such puzzles is not always realistic to expect due to the frequent lack of direct in situ observation.

## 8. Conclusions

Theories, especially those based on building a mathematical model, always imply some simplification, approximation, and idealization. Such an approach is entirely justified because the ultimate aim is usually to elaborate a simple explanation of physical or chemical phenomena (some scientists share the opinion that “if a theory is not yet simple, then it is not yet right”, i.e., unfinished and imperfect). As reflected in this work, the process of self-organization on anodized aluminum is rather complex and multifaceted, and its numerous unique aspects still need to be clarified. The formation of PAA must be viewed from both the physical and the chemical standpoints. While physics considers different transport processes (ionic, fluid etc.) and is thus the key to understanding the self-ordering dynamics, chemistry describes the conversion processes occurring in parallel (electrode reactions, chemical dissolution, surface phenomena etc.) and is thus crucial for determining the properties of the medium in which the corresponding physical processes occur. Therefore, careful consideration of the chemical reactions involved in PAA synthesis would help identify the essential mechanisms contributing to hexagonal pore formation and to isolate side processes, which are of secondary importance. When looking at such multi-component dynamic systems with a complex interplay of different parameters (temperature, types, and concentrations of dissolved electrolytes, pH, applied potential difference), a chemist starts to think about the possible electrochemical reactions, ionic and phase equilibria, local pH values in different parts of a system, isoelectric points, and coagulation or dissolution of particles, and tries to estimate the velocities of these processes from a kinetic standpoint and directions of spontaneous chemical changes under given conditions using thermodynamic criteria. In addition, there may be a number of factors affecting PAA formation that are not readily observable and measurable in situ due to the limited abilities of analytical methods under anodizing conditions, and only ex situ examination of morphological features of PAA can indirectly point to their existence. For instance, a continuous (not discrete) change of compositional and physical properties upon the transition across a phase separation can be mentioned. This can be exemplified by the gradual change of viscosity at the electrode/solution interface assumed in the electroconvective model of PAA formation (which is extensively discussed in this review work). This model represents the barrier layer during the electro-synthesis as slurry whose rheological properties may deviate from the Newtonian model (a “shear-thinning” fluid whose viscosity decreases upon the increase in the strain rate) [16,105]. This concept has been used fruitfully for the explanation of the fluid flow direction fluctuations leading to spontaneous pore branching under a steady-state anodizing regime (the Reynolds number *Re* which determines the transition between laminar and turbulent flow regimes is a function of the dynamic viscosity) [62]. After considering such various arguments, there is a feeling of confidence that the collective effort of researchers from various disciplines will bear fruit in reconciling different currently existing (and sometimes also conflicting) views regarding the PAA formation mechanism and bring a unified theory in the observable future.

In his book *The End of Certainty: Time, Chaos and the New Laws of Nature*, Ilya Prigogine (Nobel laureate, the creator of chaos and self-organization theory) wrote, “The dream of my youth was to contribute to the unification of science and philosophy.” [47]. He saw human creativity as a tool for “the amplification of laws of nature” and systematically reinforced this idea by numerous examples from physics, chemistry, and other sciences. Of course, the new scientific domain was not supposed to be self-isolated. It provided a framework for solving numerous applied problems and aimed to bring the advantages of self-organization processes into different areas of technology. In this regard, it is particularly interesting to analyze the conformity of the electroconvective model of PAA formation (which is based on elements of Prigogine’s theory) with some general philosophical principles of evolution in a dynamically changing environment. The electroconvective model, which is aimed to address a very specific problem of preparative electrochemistry, conforms well to the framework of dialectics, which can be traced back to the works of Plato and Socrates and was finally formulated by Hegel. Hegelian dialectics, which addresses the issues of being and becoming, is based on three main principles: the unity of opposites, the transition from quantity to quality, and the negation of the negation. Whereas the meanings of the former two principles are self-explanatory, the latter means continuity, the repetition of some properties at different stages of development, the connection between old and new. That is, upon transition between different states, a system in its new condition inherits some basic properties from previous conditions (imagine an upward spiral with an increasing diameter of the loops at every next level). Let us now return to the convection processes responsible for the hexagonal pattern formation upon Al anodizing. When counter-propagating ion flows arising due to Coulomb forces and the oppositely directed diffusion start to cooperate near the anode surface and form a three-dimensional network of vortices (hexagonal convective cells), this is nothing other than a demonstration of the principle of unity of opposites. When a transition between disordered and ordered regimes occurs upon the increase in the applied voltage to a certain critical value (i.e., a bifurcation point is reached and a new path to self-organization is offered to the system upon quantitative changes of one parameter), this is a clear manifestation of the principle of the transition from quantity to quality. Finally, when smaller hexagonal cells generated under mild anodizing (MA) conditions cease to exist, and larger hexagonal cells of the same convective origin emerge instead upon the transition to the hard anodizing (HA) regime, this absolutely corresponds to the principle of the negation of the negation. Thus, it can be concluded that there is no fundamental contradiction between the basic theses of the convective theory of PAA formation (or the ways in which it explains observable changes upon the variation of anodizing parameters) and the natural course of an abstract dynamic system evolution governed by the basic laws of dialectics. However, of course, there is no “absolute truth”, and the development of new theories that provide better explanations of experimental facts is a continuing process (which is the underlying principle of the scientific method). Hopefully, this review work will be able to stimulate further creative and fruitful research of this exciting direction in the interdisciplinary science which integrates electrochemistry, hydrodynamics, and elements of chaos and self-organization theory.

## Figures and Tables

**Table 1:** Suitable electrolyte compositions, corresponding anodizing regimes, and resulting PAA pore diameters *d*_p_ and cell sizes (or interpore distances *D*_int_) from selected references.

Electrolyte	Voltage ∆*U* (V)	Temperature (°C)	Pore Diameter *d*_p_ (nm)	Cell Size *D*_int_ (nm)	Reference
0.3 M Oxalic acid (H_2_C_2_O_4_)	40	0	67 ± 6 (after widening by etching in 5% H_3_PO_4_ at 30 °C for 90 min.)	99 ± 8	Masuda and Fukuda [1]
1.67 M Malonic acid (CH_2_(COOH)_2_)	110–140	0	—	the *D*_int_/∆*U* ratio varied from 2.0 to 1.8 nm V^−1^ upon increased ∆*U*	Lee et al. [74]
2–5 M Malonic acid (CH_2_(COOH)_2_)	120	5	—	300	Ono et al. [75]
1–10 vol % Glycolic acid (HOCH_2_COOH)	50–150	10–20	≈35	150 (50 V, 10 vol %, 10 °C); 320 (150 V, 1 vol %, 10 °C);	Chu et al. [76]
2–4 wt % Malic acid (C_2_H_2_OH(COOH)_2_)	220–450	10–20	—	550 (4 wt %, 220 V, 10 °C); 950 (2 wt %, 450 V, 10 °C);	Chu et al. [76]
0.3 M Acetylenedicarboxylic acid (HO_2_C−C≡C−CO_2_H)	87.5–97.5	0–60 °C	100–110	250	Kikuchi et al. [61]
0.3 M Tartronic acid (HOOC(CHOH)COOH)	110	0	48 ± 13	222 ± 24	Pashchanka and Schneider [62]
0.125 M Citric acid (C_3_H_5_O(COOH)_3_)	260–450	10–30	230	1100	Mozalev et al. [9]
3–5 M Tartaric acid (HOOC(CHOH)_2_COOH)	195	5	—	500	Ono et al. [75]
0.3 M Sulfuric acid (H_2_SO_4_)	27	0	15–40	63	Masuda et al. [2] Asoh et al. [69]
1:1 mixture of 0.3 M Sulfuric acid (H_2_SO_4_) and 0.3 M Oxalic acid (H_2_C_2_O_4_)	36	3	—	73	Shingubara et al. [77]
0.1–3.0 M Selenic acid (H_2_SeO_4_)	37–51	0	≈40	95–110	Kikuchi et al. [66]
10 wt % Phosphoric acid (H_3_PO_4_)	160	3	267	420	Li et al. [8]
0.3 M Phosphoric acid (H_3_PO_4_)	195	0	—	500	Masuda et al. [50]
0.5–2.0 M Phosphonic acid (H_3_PO_3_)	150–180	0–20	132 (under the following optimized conditions: 1.0 M, 150 V, and 20 °C)	370–440	Akiya et al. [70]
0.3 M Chromic acid (H_2_CrO_4_)	20–50	20–50	37.5–87.0 (*d*_p_ variation was achieved by changing both ∆*U* and temperature)	66.0–179.5	Stępniowski et al. [78]

## Data Availability

Data sharing is not applicable to this article as no new data were created or analyzed in this study.

## Supplementary Materials

The following are available online at https://www.mdpi.com/article/10.3390/nano11092271/s1, Figure S1: Schematic representation of the morphology and inhomogeneous chemical composition of PAA laminas, Figure S2: Examples of the experimental setups employed for the electrochemical synthesis of PAA.
