# Supplementary material for: Conceptual Progress for Explaining and Predicting Self-Organization on Anodized Aluminum Surfaces
Source: Nanomaterials (Basel). 2021 Aug 31;11(9):2271. doi: 10.3390/nano11092271 (PMC8468298; doi:10.3390/nano11092271)
Supplement: Supplementary file 1 [file nanomaterials-11-02271-s001.zip › Pashchanka_Supplementary_Information.pdf]

Supplementary Information

Conceptual Progress for Explaining and Predicting Self-Organization on Anodized Aluminum Surfaces

Mikhail Pashchanka

Department of Chemistry, Eduard-Zintl-Institute, Technical University of Darmstadt, Alarich-Weiss-Straße 12, 64287 Darmstadt, Germany; mikhail.pashchanka@gmail.com

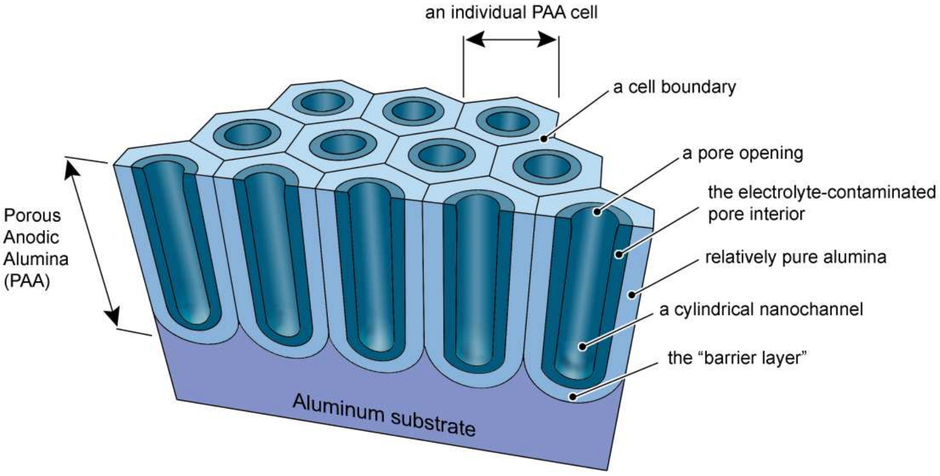

Figure S1. Schematic representation of the morphology and inhomogeneous chemical composition of PAA. A higher anionic contamination level in the cell regions directly around the pores is attributable to the anion exchange mechanism (*i.e.* the replacement of OH^- ions within PAA by acid anions from the electrolyte solution), which is discussed in detail in section 5.3 of the review. The pore openings in PAA may acquire a trumpet-like shape due to the moderate chemical dissolution of alumina in the aggressive electrolyte medium. A metallic aluminum substrate can be separated from the prepared PAA either by using the "polarity reversal" technique or by chemical dissolution (*e.g.* in a saturated $CuCl_2$ or $HgCl_2$ solution with added HCl). In order to use unsupported PAA laminas as templates for nanotechnology, the removal of the "barrier layer" may be also required. This can be performed by various dry etching (*e.g.* plasma etching) or wet etching (*e.g.* in an aqueous solution of $K_2Cr_2O_7$ and H_3PO_4) methods.

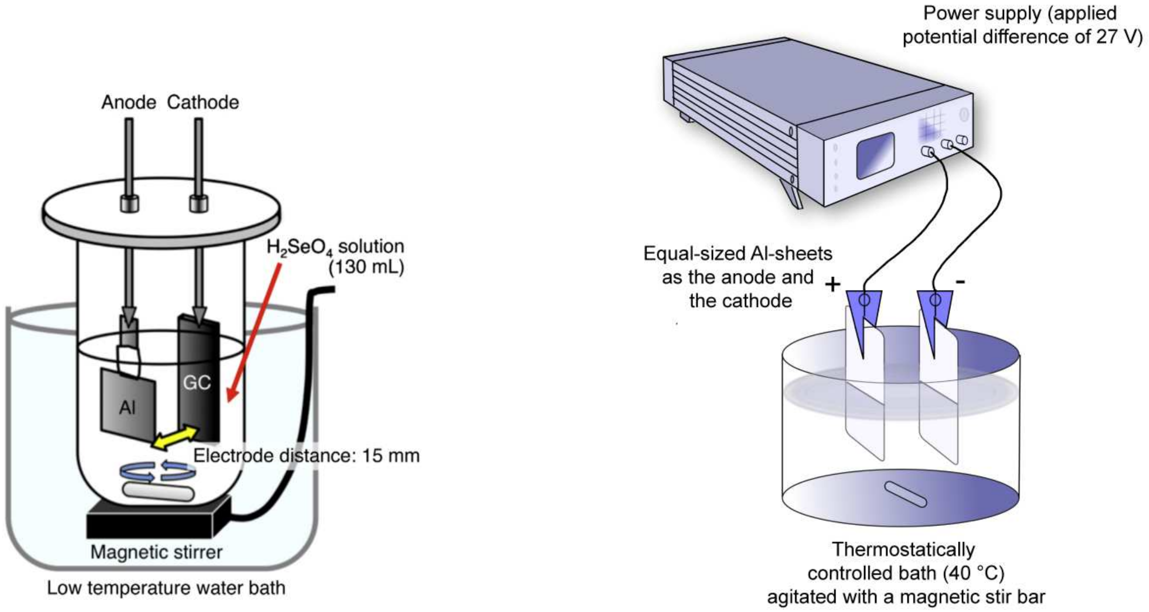

Figure S2. Examples of the experimental setups employed for the preparation of PAA laminas *via* anodic oxidation of aluminum. Left: a thermostatically controlled electrochemical cell for anodizing in 0.1-3.0 M selenic acid electrolytes at ≈ 0 °C (273 K); the contained solution is vigorously stirred for the effective heat removal from the working electrode. Right: a thermostatically controlled open bath for anodizing using a 0.3 M sulfuric acid solution; the constant temperature of 40 °C is maintained with the help of a heating circulator, the heat dissipation is facilitated by the temperature gradient between the open electrochemical cell and its surroundings. Figures are reproduced with permissions from Refs. [54] and [66] cited in the review.
